# Emotion Recognition Using Electroencephalography Signals of Older People for Reminiscence Therapy

**DOI:** 10.3389/fphys.2021.823013

**Published:** 2022-01-07

**Authors:** Lei Jiang, Panote Siriaraya, Dongeun Choi, Noriaki Kuwahara

**Affiliations:** ^1^Graduate School of Science and Technology, Kyoto Institute of Technology, Kyoto, Japan; ^2^Faculty of Informatics, The University of Fukuchiyama, Kyoto, Japan

**Keywords:** emotion recognition, EEG signals, Bi-LSTM, older people, reminiscence therapy

## Abstract

**Objective:** Numerous communication support systems based on reminiscence therapy have been developed. However, when using communication support systems, the emotional assessment of older people is generally conducted using verbal feedback or questionnaires. The purpose of this study is to investigate the feasibility of using Electroencephalography (EEG) signals for automatic emotion recognition during RT for older people.

**Participants:** Eleven older people (mean 71.25, SD 4.66) and seven young people (mean 22.4, SD 1.51) participated in the experiment.

**Methods:** Old public photographs were used as material for reminiscence therapy. The EEG signals of the older people were collected while the older people and young people were talking about the contents of the photos. Since emotions change slowly and responses are characterized by delayed effects in EEG, the depth models LSTM and Bi-LSTM were selected to extract complex emotional features from EEG signals for automatic recognition of emotions.

**Results:** The EEG data of 8 channels were inputted into the LSTM and Bi-LSTM models to classify positive and negative emotions. The recognition highest accuracy rate of the two models were 90.8% and 95.8% respectively. The four-channel EEG data based Bi-LSTM also reached 94.4%.

**Conclusion:** Since the Bi-LSTM model could tap into the influence of “past” and “future” emotional states on the current emotional state in the EEG signal, we found that it can help improve the ability to recognize positive and negative emotions in older people. In particular, it is feasible to use EEG signals without the necessity of multimodal physiological signals for emotion recognition in the communication support systems for reminiscence therapy when using this model.

## Introduction

### Background

Reminiscence therapy (RT) for older people with or without dementia is a rehabilitation method that activates the brain and has the potential to slow down the functional decline of the brain and progression of dementia (Yamagami et al., 2007). And previous research has found that RT can improve the QoL (experiencing a good quality of life), communication, cognition and emotion for older people (Woods et al., 2018). Since RT can improve the emotion of older people, an effective emotion assessment/recognition system can be used to evaluate their emotion in therapy as well as to inform activities and interventions to improve their mental health. Therefore, already a number of communication support systems based on reminiscence therapy have been applied (Sarne-Fleischmann et al., 2011; Welsh et al., 2018; Carós et al., 2020). However, when using the communication support systems, emotional assessment of the older people was usually conducted in the form of verbal feedback or questionnaires. This type of assessment is discontinuous and sometimes suffers because of the inability of individuals to describe their feelings. Automatic emotion recognition technology provides a continuous, non-invasive assessment of an individual’s emotional state. Automatic emotion recognition technology is generally implemented through facial expressions, audio technology, and physiological signal recognition. Compared to the other two technologies which are not sufficiently detailed to recognize emotions, physiological signals are more responsive to real emotions (Kim, 2007). Among the various physiological signals, Electroencephalography (EEG) signals are widely used in emotion recognition because devices for detecting the signals are non-invasive, low-cost, and wearable (Sawangjai et al., 2019). However, though EEG systems have been used in sleep, brain disease diagnosis, and cognitive training, they have not been applied much in automatic emotion recognition systems for older people (Marlats et al., 2020; Pineda et al., 2020; Choi et al., 2021; Dimitriadis et al., 2021). Current publicly available physiological signal datasets (Koelstra et al., 2011; Liu and Sourina, 2013; Zheng and Lu, 2015; Zheng et al., 2018; Lakhan et al., 2019; Bhattacharyya et al., 2020; Sangnark et al., 2021; Sharma and Bhattacharyya, 2021) for emotion computation almost always use external methods and based on young people such as IAPS image libraries, video, and audio (the mentioned datasets were summarized in Table 1) to evoke emotional changes. Few studies have implemented active methods to evoke emotions in older people. There will be differences between these passive emotional changes and those actively generated by individuals in realistic scenarios, lowering the emotional recognition rate in realistic scenarios. Thus, it is necessary to detect the EEG signals actively generated by older people during reminiscence therapy to be used for emotion calculation. Furthermore, with the advancement of deep learning technology, it is currently possible to automatically learn crucial features from a lot of data. This provides the possibility of emotion computation from dynamically collected EEG signals in realistic scenarios. Therefore, the purpose of this work is to collect EEG signals from older people during RT, and to explore the feasibility of applying deep learning models to automatically learn the EEG signal features of different emotions in order to recognize the emotions of older people.

**TABLE 1:** Emotion recognition based on different stimulus materials summary.

Signals	Subjects (Age)	Stimuli /source	Algorithm	Emotion model /features	Highest accuracy	References
EEG PER MCA	32 (26.9)	DEAP: Video (60 s)	Multimodal + Naive Bayes classifier	PAD Liking Familiarity	F1-score 60.7%	Koelstra et al., 2011
EEG	14 (-)	Audio (6 s) Picture (10 s) IADS/IAPS	SVM	PAD	Accu. 87.02%	Liu and Sourina, 2013
EEG PER	43 (16∼34)	Film clip (1 min)	K-means + multimodal + SVM classifier	Valence Arousal	Accu. V:72.35% A:68.47%	Lakhan et al., 2019
EEG	15 (23.27)	SEED: Film clip (4 min)	MFBSE-EWT + ARF classifier	Negative, positive, neutral	94.4%	Bhattacharyya et al., 2020
EEG	20 (25.75)	Music (19–66 s)	SVM RF KNN	Favored/non Familiarity Response rate	Accu. 84.64	Sangnark et al., 2021
ECG EEG	23 (26.6) 40 (28.3)	DREAMER AMIGOS (movie clip the last 30 s)	SM-SSA + RC (IP/CEC) + SVM/KNN classifier	PAD	Accu. 92.38%	Sharma and Bhattacharyya, 2021

*PER, peripheral physiological signals; MCA, multimedia content analysis; PAD, valence + arousal + dominance dimension; MFBSE-EWT, MHMS (multivariate Hilbert marginal spectrum) + FBSE (Fourier-Bessel series expansion) + EWT (empirical wavelet transform); ARF, the sparse autoencoder based random forest classifier.*

### Related Works

There are already various available emotion recognition systems. Here, the main focus is on systems that have been applied to older people. Lopes et al. (2018) extracted facial features of different emotions from older people and used support vector machines for multi-categorization of emotions. It was found that aging has a negative impact on facial expression recognition, with only a 66.6% recognition rate of sad expressions, whereas the rate of the younger age group reached 80%. Later, a speech emotion recognition system was proposed (Boateng and Kowatsch, 2020). Recordings of 87 older people were input into SBERT (sentence bidirectional encoder representations from transformers) to extract the features of the sentences to be put directly into a SVM classifier. The highest accuracy of emotion recognition in valence dimension was 57.8%. Therefore, in a later study (Dou et al., 2020), an emotion recognition model combining features of expression recognition and speech recognition was proposed to recognize four categories of emotions: happy, neutral, sad, and angry. This had an accuracy rate of more than 90%. From the above-mentioned research on contactless emotion recognition, it is obvious that firstly, as studies in Freudenberg et al. (2015) and Lopes et al. (2018) stated, facial expressions become less distinguishable due to changes in facial features from aging, such as some wrinkles and folds, which reduce the clarity of facial expressions in the elderly. Secondly, the speech signal conveys massive amounts of information and the features that best reflect emotions are difficult to find accurately and needs to be combined with other techniques to improve recognition rates. On the contrary, contact emotion recognition seems to present better results. Hakim et al. (2018) combined the oxygen saturation (SpO2) and pulse rate (PR) of older people and input the data into a SVM classifier to recognize three emotions, happy, sad and angry. Here they obtained an accuracy of 72.86%. In a later study, Awais et al. (2020) proposed an IoT-supported emotion recognition system. By inputting the EMG signals into the LSTM model, recognition of four emotions, amused, bored, relaxed and scared, had an average accuracy of 70%. However, by inputting the physiological signals ECG, BVP, GSR, RSP, SKT, EMG into the LSTM model, the recognition accuracy of the four emotions was over 95%. In another study (Meza-Kubo et al., 2016), the IAPS photo library was used as material to stimulate the emotions of older people. Alpha, beta and theta waves were extracted from the EEG signals of older people as input into a neural network to train an emotion classification model. The trained model was then used to recognize the emotions of older people while they were using a cognitive wellness system. The accuracy of two-emotion recognition, pleasant or unpleasant, ranged from 60.87 to 82.61%. Because this accuracy range is consistent with other emotion recognition techniques and less costly than the others, the authors proposed that it is feasible to recognize emotions for the older people using EEG signals. Later studies have even combined physiological signals (blood pressure, sweat, respiratory rate), facial expressions (camera) and sound (microphone) to recognize emotions (Castillo et al., 2014). The emotional state is then transmitted to actuators to achieve a real-time and continuous monitoring system. The emotions of older people are then regulated by adjusting music and lights. From previous works (a summary is shown in Table 2), it was found that the effect of contact emotion recognition is better than non-contact emotion recognition and that multimodal physiological signals were usually better than unimodal physiological signals to recognize emotion. However, for older people, too many acquisition devices easily becomes discomforting. With the development of deep learning, widely used in physiological signals analysis, the accuracy of emotion recognition has been greatly improved. This due to the fact that deep learning networks have better emotion computation capabilities compared to traditional methods of machine learning (Zheng and Lu, 2015; Thammasan et al., 2016). Deep learning models can automatically compute complex features of physiological signals that are easily ignored by manual feature extraction techniques. Overall, emotional expression is a multi-component complex process that consists of three components: internal experience, external expression, and physiological activation (Keltner and Gross, 1999). For the emotion recognition system of older people, external expressions are unreliable due to changes in the morphological features from facial aging. In contrast, physiological signals are unable to be suppressed or hidden, providing a reliable response to psychological feelings. If the EEG signals are collected from older people as an objective response to their emotions and supplemented with a completed subjective self-assessment form, it is possible to reflect emotions from internal experiences and physiological stimuli, providing richer data for further emotion calculation.

**TABLE 2:** Emotion recognition systems applied to older people summary.

Signals (source/stimuli)		Subjects (Age)	Algorithm	Emotion model	Highest accuracy	References
Contact	Non-contact					
	Expression (Lifespan database)	778 18–59 60–93	Viola-Jones-Haar + Gabor + SVM	Neutral Happiness Sadness	95.24% 90.32% 88.57%; 84.61% 80%; 66.6%	Lopes et al., 2018
	Speech (USOMS-e database)	87 (71.01)	Pretrained CNN /BERT/SBERT + SVM	Valence Arousal	57.8% 47.5%	Boateng and Kowatsch, 2020
	Speech, Expression (HCI)	5 (50–60)	Bi-LSTM Xception Fusion	Negative Positive	Speech: 91% Expression: 90% Fusion: 94%	Dou et al., 2020
EEG (IAPS)		8 (72.3)	Trained neural network	Pleasant Unpleasure	82.61%	Meza-Kubo et al., 2016
SpO2, PR (Video)		31 (63.8)	Statistical feature + KNN/SVM	Happy; Sad; Angry	SVM:72.86% KNN: 71.93%	Hakim et al., 2018
EMG, ECG, BVP, GSR, RSP, SKT (Video/IoT)		30 (-)	LSTM	Amused Bored Relaxed Scared	EMG: F1-score 70% Fusion: F1-score 95%	Awais et al., 2020

*SpO2, oxygen saturation; PR, pulse rate; HCI, Human-computer interaction; IoT, Internet of Things; about the highest accuracy in the table unless otherwise noted, all are Accuracy.*

### Present Work

The main work and contributions to this paper are as following:

(1) Based on realistic scenarios in the implementation of reminiscence therapy, the EEG signals actively generated by the emotional response in older people were collected. This can subsequently be used as an emotional computational dataset for a conversation support system.

(2) An emotional self-assessment scale was designed for application to older people. In a conversation system, the effect of classifying emotions using the ratings of pleasure and stress was found to be more suitable for classifying emotions in older people compared to the Valence-Arousal model.

(3) It was found that using EEG signals without the necessity of multimodal physiological signals for emotion recognition in the communication support systems for older people during RT is feasible. At the same time the four EEG signal channels (F7, F8, T7, T8) near the ear (corresponding to the position of eyewear) based on Bi-LSTM model resulted in 94.4% accuracy in emotion recognition. This design proposal improves the possibility of acquisition of high-quality signals (due to the avoidance of the hair) and it is possible to reduce the weight of the device which is beneficial to reduce the discomfort of old people when having to wear such sensing devices for a long time.

## Materials and Methods

### Materials and Participants

For older people, old public photographs can stimulate autobiographical memories to achieve the effect of reminiscence therapy (Wu et al., 2020). Therefore, four themes of old photographs were collected: Showa era, landscape, food and festival. Because these four themes of photos are known by most Japanese older people which can awaken their previous memories. In order to control the effect of the different photo types on each older person, all photos were organized in the form of a questionnaire (details of the photos can be found in the Supplementary Material), and the older people who participated in the experiment were asked to answer the questionnaire 1 week beforehand. The question under each photo was: Would you like to talk to young people when looking at this photo? (1) want to, (2) don’t want to (or no feeling). The photos were then divided into two categories based on the answers (personal preference) to the questionnaire. Eighteen photos were randomly selected from each of the two categories corresponding to each older person to make up the individual reminiscence therapy materials. And then the older people and young people participating in the experiment were randomly grouped into pairs. They will talk around each RT material for 1 min and collect EEG signals from older people. In addition, in the same period of work (Jiang et al., 2021), we found that no statistically significant difference in the personal preference (liking and familiarity) of photos for the older people on the pleasantness of the conversation. Therefore the personal preference on photos was not taken into account as EEG features when evaluating conversation emotion. The Japanese version of the GDS depression quantification form (Sugishita and Asada, 2009) was filled out by elderly people before the experiment. The GDS indicates depressive tendencies when the score is greater than 6. None of the older people in this experiment showed depressive tendencies (mean 2.0, SD 1.79; range 0–5). Details of the information about the materials and participants on Table 3.

**TABLE 3:** Experiment summary.

Photo conversation stimuli	
Number of photos	36
Photo content	Showa era, landscape, food and festival
Photo Conversation	1 min
Experiment information	
Number of participants	11 (O) and 7 (Y)
Number of males	6 (O) and 5 (Y)
Number of females	5 (O) and 2 (Y)
Age of participants	O: 66–82 (M = 71.25, SD = 4.66); Y: 20–24 (M = 22.4, SD = 1.51)
Rating scales	Arousal, Valence, Stress
Rating values	−4–4, −4–4, 1–7
Recorded signals	8-channel 256 Hz EEG

*The old people (O) from Silver Human Resources Center, Kyoto, Japan; The young people (Y) from Kyoto Institute of Technology, Kyoto, Japan.*

### Experiment Procedure

**FIGURE 1:**
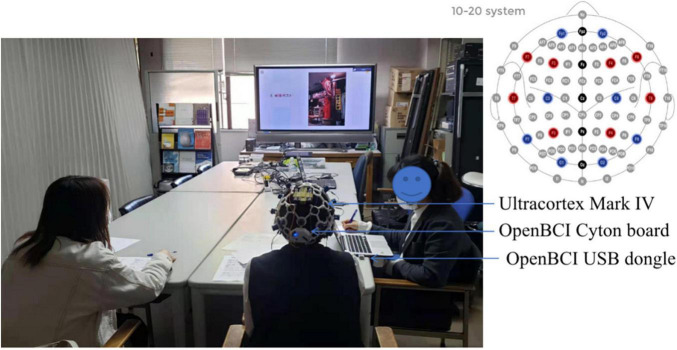
Two participants during the experiment.

**FIGURE 2:**
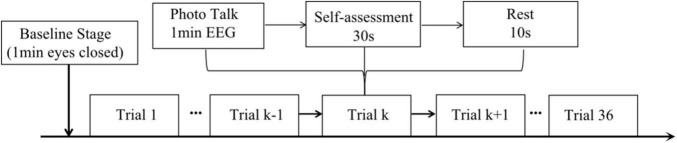
Detail of the experiment procedure.

The experiments related to this study were approved by the 116th and 122nd Kyoto Institute of Technology Ethics Committee for Scientific Research Involving Human Subjects (No. 2020-18, and 2021-03). All participants agreed and provided their written informed consent to participate in this study. The experiment was conducted in a separate meeting room with a comfortable environment and soft light. It also has good soundproofing to minimize the influence of the external environment. Prior to the conversation, the student explained to the older adult the entire experiment procedure. Then the older adult was fitted with an Ultracortex Mark IV electrode cap (PLA, 3d printing, head circumference 48–58 cm, 10–20 system electrode positions), the electrodes adjusted to the correct position and the ground electrodes attached to both ears of the subject. The older adult and the student were seated next to each other approximately 2 m from the screen. The conversation started with the older adult’s eyes closed for 1 min for a baseline measurement and then the two participants talked about the photo displayed on the screen for 1 min (Figure 1 shows the participants in the experiment). At this time, the EEG signals from the Mark IV electrode cap were received by an OpenBCI Cyton biosensor board, and the signals were transmitted to the computer for real-time display and recording *via* the OpenBCI USB dongle. After the 1-min conversation, the older adult used 30 s to fill out the self-assessment form (shown in Appendix I and the details can be found in Supplementary Material), followed by a 10-second break. After that, the participants talked about the next photo, for a total of 36 photos. The details of procedure are shown in Figure 2.

As the OpenBCI Cyton board, which collects physiological signals, can receive 8 channels of signals with a sampling frequency of 250 HZ, 8 electrodes were installed on the electrode cap (the positions marked in red in the upper right corner of Figure 1, 10–20 system). The corresponding brain region and names of the electrodes are frontal, F3 and F4; left frontal, F7; right frontal, F8; left temporal, T7; right temporal, T8; parietal occipital, P3 and P4. These electrodes were chosen due to a previous study (Martinez et al., 2013) where the authors demonstrated through extensive experiments that the eight channels most suitable for emotion recognition are AF3, AF4, F3, F4, F7, F8, T7 and T8. Since AF3 and AF4 are not available in Mark IV and these two positions are more influenced by eye movements, they were replaced with P3 and P4. These positions correspond to brain areas related to the function of short-time verbal logic and short-time mathematical logic which affect conversation. At the end of the experiment, each subject was asked to evaluate the comfort of the Ultracortex Mark IV in terms of weight, electrode pressure, wear time, and device improvement (shown in Appendix II and the details can be found in Supplementary Material).

### Emotion Classification Model and Subjects Self-Assessment

In order to distinguish the emotional states of older people by the emotion recognition system, the EEG signals and the emotional states must first be corresponded one by one by in the emotion classification model. Therefore, it is necessary to design an emotion self-assessment form based on the emotion classification models. All existing emotion classification models can be divided into the following two main categories: (1) Discrete emotion model, such as Ekman’s model (anger, disgust, fear, happiness, sadness, and surprise) which is very useful for the application of facial emotion detection; (2) Dimensional emotion model, such as Hourglass model which consists of 20 categories (half positive and half negative) in four independent dimensions, each emotion is presented in a pair of words displaying the distinct similarities and the differences of emotion (Zad et al., 2021). It can be found that the dimensional emotion model will express more emotions than the discrete emotion model and different emotion models are applicable in different emotion tasks. Therefore, it is necessary to design the self-assessment form by choosing a suitable emotion classification model based on task requirements. Among the already designed self-assessment forms related to our task, the SAM scale [Self-Assessment Manikin (Lang, 1980)] is a self-emotion assessment scale using cartoon villain images and it has been proven to be highly correlated with physiological signals. It designed based on the PAD model, it has a total of three dimensions: pleasure, arousal, and dominance. Individuals need to choose the most suitable picture for their current state in each dimension. Through a preliminary survey, we found that SAM scale is too complicated for older people to understand. They had difficulty understanding the dominance dimension and did not know how to choose. They tended to choose one of the cartoon images, overlooking the fact that the position between the two cartoon images was also an option. Therefore, in this experiment, we removed the dominance dimension from the SAM and kept only the pleasure and arousal dimensions (Russell studied the PAD model and found that these two dimensions can represent most of the different emotions, which is also called the Valence-Arousal (VA) model (Russell et al., 1989). We then converted the cartoon character into a nine-value scale (values ranging from −4 to 4, representing “not at all” to “extremely”). It was proved in related studies that whether or not the older people felt stress in the conversation was also an important factor that affects emotions during reminiscence therapy (Iwamoto et al., 2017). Therefore, we added the stress level rating (values from 1 to 7, indicating “no stress at all” to “extremely”) to the self-assessment form (shown in Appendix I and the details can be found in Supplementary Material).

### EEG Dataset Classification

**FIGURE 3:**
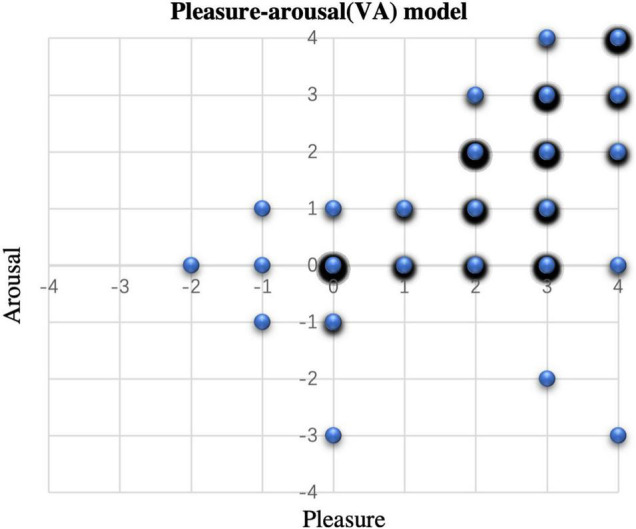
Distribution of the conversation emotions on the VA model.

To distinguish the emotional states of the older people by the emotion recognition system, the EEG signals were first corresponded to the emotion space of the VA model one by one. Therefore the 396 samples (11 subjects × 36 conversations) were collected, corresponding to the four quadrants of the VA model and represented the four emotion categories: (1) Samples in high valance (pleasure) dimension and high arousal (excited) dimension represent the happy emotion; (2) Samples in high valence dimension and medium arousal dimension represent the relaxed emotion; (3) Samples in low valance (unpleasant) dimension and high arousal dimension represent the frightened emotion; (4) Samples in low valance dimension and medium arousal dimension represent the boredom emotion. However, as shown in Figure 3 (larger circles and more black shading represent a larger number of samples), the VA model does not classify the emotions of older people well. Almost all of them are distributed in the first quadrant and on the number axis (241 and 121 samples respectively). Therefore, we replaced the arousal dimension with the degree of stress. In this case 195 out of 396 samples were divided into 2 categories as shown in Table 4. Samples with Pleasure value greater than 0 and Stress value less than or equal to 1 were considered as positive emotions. While samples with Pleasure value less than 0 or a Stress value greater than 2 were considered as negative emotions.

**TABLE 4:** Distribution of the conversation emotions on PS values.

Classify	Conditions	Total
Positive	Pleasure value > 0 and Stress value ≤ 1	126
Negative	Pleasure value < 0 or Stress value > 2	69

### Emotion Classifiers Based on LSTM and Bi-LSTM

**FIGURE 4:**
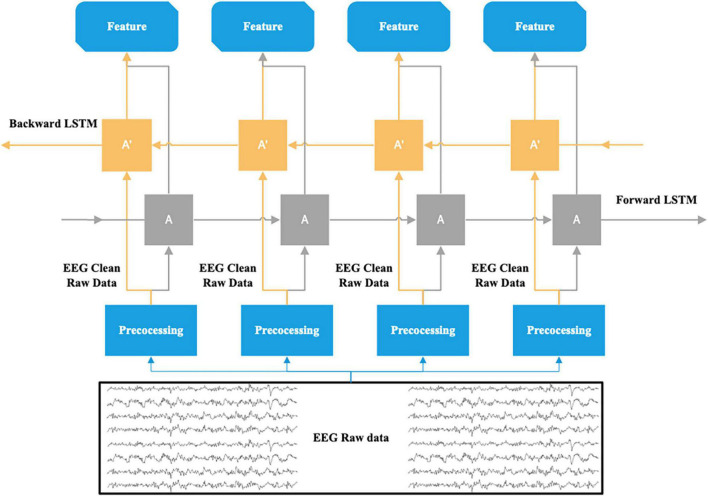
The Emotion classification of EEG raw data based on Bi-LSTM.

For the depth model selection, we used LSTM (Long Short-Term Memory) and Bi-LSTM (Bidirectional LSTM) models to automatically extract features for sentiment computation on the binary sentiment data. The reason is that the emotional change during a conversation is a long-period emotional change and the current emotional state will be influenced by the previous emotional state. EEG can reflect the emotional state, thus there is also a correlation between the EEG responding to the previous emotion and the EEG of the current emotion. According to related studies (Li et al., 2016), the contextual information of EEG can be used in emotion recognition. Therefore, the EEG data containing emotional changes can be regarded as a feature sequence containing contextual correlations. First, we chose to apply the LSTM model to compute the relevance of the contextual information of the EEG feature sequence to implement sentiment classification. This is because it has an advantage over the traditional RNN in the processing and classification of temporally correlated signals (Hochreiter and Schmidhuber, 1997). There are three gates in the basic memory unit of the LSTM model: the input gate (which decides which parts of the new input information are updated), the forgotten gate (which decides which parts of the previously stored information are discarded), and the output gate (which decides the final update and output). This makes it possible to maintain and transfer the critical features in the temporal data effectively throughout the long-term computation of the classification network. However, both RNN and LSTM are only able to predict the output of the next moment based on the temporary information of the previous moment. Emotional changes are generally not dramatic but rather calm and stable, and it takes time for the potential changes resulting from emotional changes to be transmitted to the cerebral cortex. Sometimes there is a lag in the emotional response in the EEG data, so the emotional state at that moment may also be related to the “future” state. In order to simultaneously compute the sequence of the “past” and “future” features of the EEG data acting on the present emotion, we chose to apply the Bi-LSTM model to compare with the LSTM model. The Bi-LSTM is composed of two superimposed LSTM layers, which can be divided into a forward (“past” state) and a backward (“future” state) LSTM in the direction of time. The new information input for each moment is computed independently in the two LSTM layers, and then jointly input into the output layer for computation, determining the output result and then updating (Siami-Namini et al., 2019). The EEG data in the process of collecting may include noise which must be pre-processed (described in the next section) and then input into the model. The basic structural unit of the Bi-LSTM applied in this paper is shown in Figure 4.

## Results

### Dataset and Pre-processing

According to the results of emotion classification based on the self-assessment forms, the corresponding EEG signals (60 s × 250 Hz × 8 Channels) were also divided into two categories as shown in Table 4. Thus, the whole EEG dataset includes 126 positive emotion samples and 69 negative emotion samples with 2 arrays named dataset 1 (Table 5). There is one more aspect to consider: the dry electrodes embedded in the cap may cause head pain after wearing the cap for a long time. This problem should be alleviated if it is improved in the following three ways: reducing the weight of the wearable device, reducing the number of electrodes, and making the system using a soft textile electrode. Therefore, in order to explore the possibility of later embedding only 4 electrodes in an easy-to-wear, lighter portable EEG device to achieve emotion recognition, we extracted the data from the channels corresponding to F7, F8, T7, and T8 (corresponding to the position of eyewear) to make as dataset 2 and the data from the channels corresponding to F3, F4, F7, and F8 (corresponding to the position of a headband) to make as dataset 3.

**TABLE 5:** Data arrays for subjects.

Array name	Array shape	Array contents
Dataset 1	195 × 8 × 15,000	Trials × Channels × Data
Labels	195 × 2	Trials × Label (positive and negative)

The whole pre-processing process is carried out using the SciPy library in python. First, because the format of EEG signals data collected by OpenBCI GUI in real time was TXT format, all data first was converted to the CSV format for subsequent processing. Then the butterworth bandpass (1–45 Hz) was used to remove major noise and artifacts from the EEG signal. In addition, the I.F. interference in Kansai, Japan, is 60 HZ, which is not considered here because the previous step has been performed. Finally, the EEG signals were detrended with a Chebyshev I high-pass filter to remove baseline drift.

### LSTM and Bi-LSTM Model Results

**FIGURE 5:**
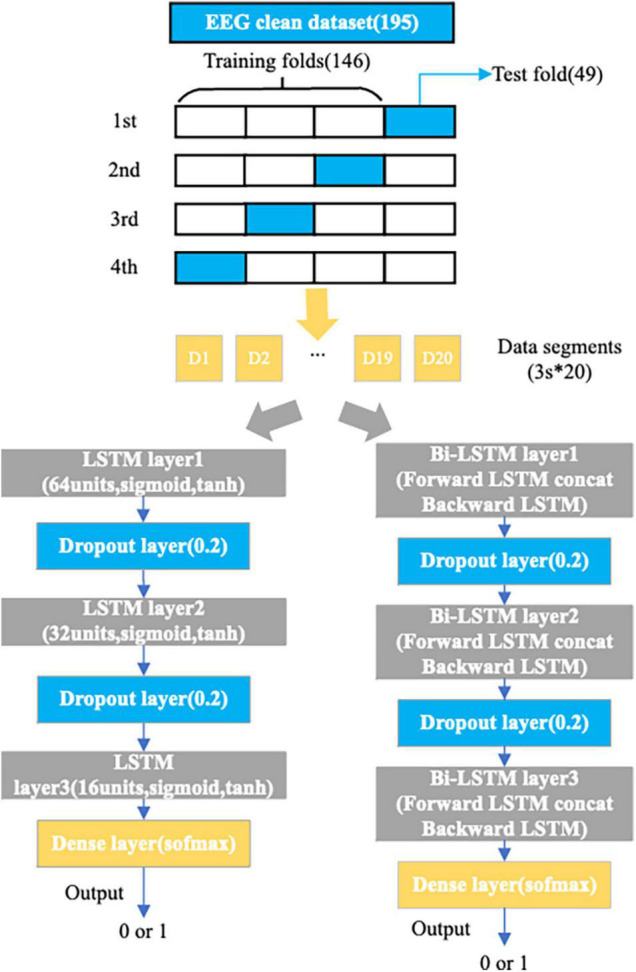
Details of the LSTM and Bi-LSTM models.

The training and learning process of the model were done by the python libraries of keras, sklearn and numpy. The structure of the LSTM model designed here for EEG data consists of 3 LSTM layers (64, 32, and 16 units) for mining contextual relevance in the input EEG feature sequence: 2 dropout layers with probability 0.2 to avoid overfitting and a dense layer for integrating information for binary classification. The structure of the Bi-LSTM model designed here for EEG data consists of three bidirectional LSTM layers, each including outputs in the forward LSTM direction and backward LSTM direction fed in series to the next structure for mining the influence of “past” and “future” states of the present state in the EEG feature sequence. The other structures are consistent with the above LSTM model. All the LSTM layers in both models have two activation functions, sigmoid is the activation function for the three gate mechanisms (input gate, forgotten gate, output gate) and tanh is the activation function for the input of information and the current hidden state. As the amount of our available data is small, if we directly divide data into different sets (training, validation and the test sets), it will severely damage the data density and the performance of the model. In this case, cross-validation can be used to solve this problem (Kale et al., 2011). Therefore, for our dataset, both models use fourfold cross-validation. All samples (126 positive samples and 69 negative samples) were randomly divided into four equal subsets, and one of the four subsets was used as the validation set (49 samples). The remaining three subsets were used as the training set (146 samples). This means that the subsamples in the training and the validation sets were totally independent but subject-dependent. The cross-validation is repeated four times to average the results to ensure accuracy. The structure of the two designed models is shown in Figure 5.

Dataset 1 has a total of 195 samples, each of which consists of 15,000 sample points (60 s × 250 Hz) from 8 EEG channels and a label. Each sample is divided into 20 segments (3-s), so that the structure of the dataset 1 became 195 samples, labels, 20 segments and 3 s × 250 Hz × 8 channels. The Datasets 2 and 3, we also split each sample of each channel EEG data into 20 3-s segments, so the structure of the datasets became 195 samples, labels, 20 segments and 3 s × 250 Hz × 4 channels.

The hyperparameters of the two models for datasets were set as follows: (1) Loss function: Categorical-cross-entropy; (2) Optimizer: SGD, RMSprop and Adam optimizer; (3) Learning rate of optimizer: 0.01, 0.001, 0.000 (it has important impact on the optimizer); (4) Batch size: 10; (5) Epoch: Epoch = Max early-stopping epoch (T1, T2, T3, T4). About the early-stopping method, it is used for the model to obtain the best generalization performance based on the epoch (Bisong, 2019). Specifically the method is used to calculate the model’s performance on the validation set during training and stop training when the model’s performance on the validation set starts to decline. It was set up through the karas.callbacks module: EarlyStopping method (monitor = val_loss, mode = min, mim_delta = 0, patience = 2) which means during the model training, the validation set has two consecutive epochs when the loss value does not drop, and then the training process is stopped. In addition, as four-crossover validation is used, each time there are four early-stopping epoch values (T1, T2, T3, T4), the maximum of the four epoch values is chosen as Epoch.

After comparing the accuracy of validation set with optimizers having different learning rate and integrating the generalization ability of the models, the optimal parameters suitable for the models on dataset 1 are obtained: LSTM: SGD optimizer with the learning rate of 0.001, 30 epochs. The mean accuracy for dataset 1 is 90.8% in positive and negative emotions; Bi-LSTM: SGD optimizer with the learning rate of 0.001, 60 epochs (too big value of the Ir and too small value of the epoch may cross the local minimum and result in no convergence). The mean accuracy for dataset 1 is 95.8% in positive and negative emotions. As the result of the Bi-LSTM model for dataset 1 was better than the LSTM model, Bi-LSTM model was used for the other datasets. Dataset 2 was inputted into the Bi-LSTM model (RMSprop optimizer (Ir = 0.0001), batch size = 20, epochs = 40) with the highest accuracy of 94.4% in positive and negative emotions. And for dateset3, the Bi-LSTM model (RMSprop optimizer (Ir = 0.0001), batch size = 20, epochs = 30) reached the highest accuracy rate of 69.2%. The comparison of the results between datasets and the parameters of models were shown in Table 6.

**TABLE 6:** Comparison of accuracy results between datasets and parameters of models.

Dataset	Channels	Models	Optimizer	Accuracy (%) (Epoch)		
				Ir = 0.01	Ir = 0.001	Ir = 0.0001
1	F3, F4, F7, F8, T7, T8, P3, P4	LSTM	SGD	98.3%(10)	90.8%(30)	73.3%(120)
			RMSprop	–	–	81.0%(30)
			Adam	–	–	84.8%(40)
1	F3, F4, F7, F8, T7, T8, P3, P4	Bi-LSTM	SGD	98.5%(35)	95.8%(60)	86.7%(180)
			RMSprop	–	–	81.5%(30)
			Adam	–	–	85.6%(20)
2	F7, F8, T7, T8	Bi-LSTM	SGD	–	93.5%(40)	86.4%(160)
			RMSprop	–	–	94.4%(40)
			Adam	–	–	87.1%(30)
3	F3, F4, F7, F8	Bi-LSTM	SGD	64.1 (10)	65.1%(30)	64.6%(100)
			RMSprop	–	–	69.20%(30)
			Adam	–	–	66.1%(15)

*“–” represents the loss value of validation set is greater than 1 or accuracy equal to 100% with very few epochs (overfitting). The underlined values, represent the optimal parameters chosen for the models and the corresponding accuracy of emotion recognition.*

### Ultracortex Mark IV Electrode Cap Comfort Evaluation Results

The results of Q1 (mean, 1.54, SD, 0.69) indicated that the weight of the Mark IV electrode cap is bearable and not too heavy. The results of Q2 (mean, 2.18, SD, 0.60) indicated that the dry electrode is a bit painful and not suitable for prolonged use. The results of Q3 (mean, 2.73, SD, 0.65) indicated that the subjects can generally wear Mark IV electrode cap for 30 min to an hour. The results of Q4 (mean, 2.09, SD, 0.70) suggested that if painless electrodes are embedded in a more portable wearable device, the subjects would be willing to wear it for a long time and will not reject collecting EEG data for emotion recognition. The specific Q1-Q4 questions and options are shown in Appendix II.

## Discussion

### Principal Results and Limitations

Emotions generated by the subjects for each of the four quadrants of the VA model out of 396 samples: 241 samples in the first quadrant (happy), 1 sample in the second quadrant (panic), 1 sample in the third quadrant (boredom), 2 samples in the fourth quadrant (relaxed) and 151 samples on the axis, which indicate that they could not effectively be distinguish as specific emotions. By removing the arousal dimension and adding the stress levels, emotions were divided into positive and negative emotions, 126 and 69 valid samples respectively. This is because for older people, when seeing old photos, they are more inclined to recount unforgettable and pleasant memories of the past, and most of them will generate or amplify positive emotions (Cappeliez et al., 2008). Meanwhile older people’s mood changes are not as pronounced and their emotional state shifts to the negative when they are under pressure that the conversation is ending early. So, for the conversation support system with emotion recognition, what needs to be considered is the level of pleasure brought by the memories and the stress felt during the conversation.

Dataset 1 (8 channels EEG signals) achieved the highest recognition accuracy of 95.8% in positive and negative emotions through the Bi-LSTM model (SGD, Ir = 0.001, batch size = 10, epoch = 60). And through the LSTM model (SGD, Ir = 0.001, batch size = 10, epoch = 30) got the accuracy of 90.8%. The results showed that use of additional layer (backward LSTM) of training would improve the accuracy of emotion recognition. At the same time, a phenomenon can be found that the training speed of Bi-LSTM was slower than LSTM, it means Bi-LSTM model requires access to more epochs of data to reach the equilibrium. This also proves from the side that Bi-LSTM model may be acquiring additional features but LSTM model is unable to obtain. In addition, by comparing the model in this work with other models used in prior studies (shown in Table 7), we found that compared to approaches which used EEG feature extraction and selection and then inputting the results into the classifier for emotion classification (Li et al., 2018; Bhattacharyya et al., 2020), using deep neural networks (Sheykhivand et al., 2020; Zeng et al., 2020) for automatic feature extraction and classification seems to perform better; The second, the multimodal physiological signals are not always better than unimodal signals for emotion recognition. The study Sheykhivand et al. (2020) and the proposed method in this work performed better than studies based on EEG and ECG signals for emotion recognition. However, it is undeniable that in small subjects, emotion recognition by multimodal physiological signals seems to be more advantageous, as shown in previous study Zeng et al. (2020) the accuracy of emotion prediction reached 95% with only 5 subjects based on EEG and ECG signals. Meanwhile, it can be noticed in Table 7 that, the emotion recognition accuracy of the Bi-LSTM model in this work is less than the CNN-LSTM model. The main reasons probably are firstly there are more subjects than this work and secondly the CNN-LSTM model consists of 10 convolutional layers and 3 LSTM layers, while in this work it consists of 3 bi-directional LSTM layers. Therefore the trainable parameters in this work are much lower than the CNN-LSTM. Therefore, the proposed Bi-LSTM model perform better for emotion recognition in older people than most model. This indicates that the Bi-LSTM model is better at recognizing emotions by taking into account the “past” and “future” emotional states to discern the present emotional state. Therefore, we can improve the accuracy of the Bi-LSTM model by further collecting EEG emotion data to learn about it. Moreover, compared to other physiological signal acquisition sensors, EEG acquisition devices are very low cost and can be used to continuously and dynamically collect signals that reflect the emotions of the elderly for the purpose of monitoring their emotional changes. Therefore, it is feasible to use EEG signals without the necessity of using multimodal physiological signals for automatic emotion recognition of older people in communication support systems.

**TABLE 7:** Comparisons with prior work in emotion recognition.

Network model	Physiological signals	Subjects (dataset)	Accuracy (%)
SVM (Li et al., 2018)	EEG	15 (SEED)	90.40%
MFBSE-EWT + ARF (Bhattacharyya et al., 2020)	EEG	15 (SEED)	94.4%
CNN-LSTM (Sheykhivand et al., 2020)	EEG	14 subjects	97.42%
SM-SSA + RCs (IP/CEC) + SVM/KNN (Sharma and Bhattacharyya, 2021)	EEG, ECG	23 (DREAMER) 40 (AMIGOS)	92.38%
LSTM (Zeng et al., 2020)	EEG, ECG	5 subjects	95.00%
Bi-LSTM (proposed method)	EEG	11 subjects	95.80%

*MFBSE-EWT, MHMS (multivariate Hilbert marginal spectrum) + FBSE (Fourier-Bessel series expansion) + EWT (empirical wavelet transform); ARF, the sparse autoencoder based random forest classifier; SM-SSA, sliding mode singular spectrum analysis; RCs, reconstructed components; IP, information potential; CEC, centered correntropy.*

According to the results of the Mark IV electrode cap comfort evaluation, it was found that the current dry electrodes embedded in the cap cause pain after wearing the cap for a long time. This problem should be alleviated if it is improved in the following three ways: reduce the weight of the wearable device, reduce the number of electrodes, and make it using a soft textile electrode. The accuracy of dataset 2 (F7, F8, T7, T8) and dataset 3 (F3, F4, F7, F8) is 94.4 and 69.2% respectively. The reason that the results of dataset 3 are not as good as dataset 2 is that there are more hairs at the electrode locations of F3 and F4 and they cannot easily be in direct contact with the scalp. Therefore, the collected signals were not as high quality as at the electrode locations of T7 and T8. Although the highest accuracy of 4-channels (94.4%) were not better than other studies, it is very close to them. In future research, we can make these four electrodes into soft textile electrodes embedded in lightweight eyewear/headband. We can then collect a large amount of EEG data or combine them with other easily/comfortably acquirable physiological signals such as heartrate through a smart watch to improve the accuracy of emotion recognition.

However, it should be noted that this study is only a preliminary feasibility study and there are some limitations. Only the usual pre-processing of EEG signals was done to remove the major artifacts and noise. In fact, in the conversation, some movements above the neck may have an effect on the EEG signal. Initially, we also tried applying independent component analysis (ICA) to remove the EEG artifacts (motion, EOG and facial muscle) in the preprocessing stage. However, after pre-processing the EEG signals with ICA and using them in the models, the accuracy was found to be around 90%. This is slightly lower than the EEG signals without ICA. The reason may be the face muscle activity during the conversation continuously and inevitably has some effect on the EEG signals. After removing them all, it was found that some emotional information was also removed. As such, we decided not to apply ICA to remove the artifacts, and thus it was not reported in the remaining sections. In fact, similar result were found in the study Becker et al. (2017), in order to assess the important of removing EOG artifacts before feature extraction and classification, the author compared the valence recognition performance for EEG data with ICA preprocessing and without ICA. It was found that with ICA preprocessing has little impact on the classification result. In addition, as the pre-processed EEG signals were directly input into the LSTM and BI-LSTM models, there is a possibility that the emotion features of the conversation may be mixed with other features generated by brain activity. It is not clear whether the model learns only the emotional information features in EEG or other EEG features as well. Considerably more work will need to be done to determine it.

### Conclusion and Future Work

Due to the current prevalent use of subjective emotion evaluation in communication support systems based on reminiscence therapy, the feasibility of using EEG for automatic emotion recognition of old people was explored. It was found that the VA emotion classification model is not suitable for old people communication support systems with emotion recognition. However, using of the pleasure level of RT and the stress level of the conversation is more conducive to the emotion classification of older people in the communication support systems. In addition, the Bi-LSTM model was found to perform better than most previous work in classification of positive and negative emotions with an accuracy rate of 95.8% in the subject-dependent condition. It achieves this by tapping into the influence of “past” and “future” emotional states in the EEG signal on the present emotional state. Therefore, considering the demand for comfort of older people during physiological signals acquisition for emotion recognition, it is feasible to use EEG signals without the necessity of multimodal physiological signals in communication support system for reminiscence therapy. Especially, the four EEG channels (F7, F8, T7, T8) near the ear (corresponding to the position of eyewear) based on the Bi-LSTM model resulted in a decent accuracy rate reaching 94.4% in emotion recognition. This design proposal improves the possibility of acquiring of high-quality signals (due to the avoidance of the hair) and allows us reduce the weight of the device. To further evaluate and improve the model proposed in this work, in our later studies, we will recruit new subjects to create a subject-independent validation datasets to evaluate the model. Afterwards, we would further analyze the EEG dataset in the time and frequency domains to find out which features can better express the emotional changes of older people, and then use the Bi-LSTM model to explore the information with more specific emotional features to improve the emotional recognition performance of the model.

## Data Availability Statement

The raw data supporting the conclusions of this article will be made available by the authors, without undue reservation.

## Ethics Statement

The studies involving human participants were reviewed and approved by the 116th and 122nd Kyoto Institute of Technology Ethics Committee for Scientific Research Involving Human Subjects (Nos. 2020-18 and 2021-03). The patients/participants provided their written informed consent to participate in this study.

## Author Contributions

LJ and NK designed the experiments. LJ carried out the experiments. LJ and PS analyzed the results. LJ and DC prepared the manuscript. All authors contributed to the article and approved the submitted version.

## Conflict of Interest

The authors declare that the research was conducted in the absence of any commercial or financial relationships that could be construed as a potential conflict of interest.

## Publisher’s Note

All claims expressed in this article are solely those of the authors and do not necessarily represent those of their affiliated organizations, or those of the publisher, the editors and the reviewers. Any product that may be evaluated in this article, or claim that may be made by its manufacturer, is not guaranteed or endorsed by the publisher.

## Appendix

**I. Self-assessment Form**

(1) Did you have fun during the conversation you just had?

−4 to 4, nine ratings, from “not at all” to “extremely”

(2) Did you get excited during the conversation?

−4 to 4, nine ratings, from “not at all” to “extremely”

(3) Did you feel burdened (stressed) by the conversation?

1 to 7, seven ratings, from “not at all” to “extremely”

**II. Ultracortex Mark IV Wearable Comfort Evaluation**

(1) Do you think the electrode cap is heavy?

A. Not at all-1 B. A little-2 C. Heavy-3 D. Extremely-4

(2) At this point, do you feel any pain where your scalp touches the electrodes?

A. Not at all-1 B. A little-2 C. Painful-3 D. Extremely-4

(3) How long do you think you can wear the Ultracortex Mark IV?

A. No more than 10 min B. 10–30 min C. 31–60 min D. Over 1 h

(4) If soft textile electrodes were embedded in wearable glasses or headbands, would you be willing to wear them for long periods of time during emotion recognition?

A. Extremely B. A little C. Reluctant D. Totally reluctant

Options A, B, C, D are recorded as 1, 2, 3, 4 points respectively.
