# Supplementary material for: Emotion Recognition Using Electroencephalography Signals of Older People for Reminiscence Therapy
Source: Front Physiol. 2022 Jan 7;12:823013. doi: 10.3389/fphys.2021.823013 (PMC8777059; doi:10.3389/fphys.2021.823013)
Supplement: Supplementary file 1 [file Data_Sheet_1.PDF]

# Supplementary materials

# Emotion Recognition Using EEG Signals of Older People for Reminiscence Therapy

**Lei JIANG[1], Panote Siriaraya[1], Dongeun Choi[2], Noriaki Kuwahara[1*]**

[1]1Graduate School of Science and Technology, Kyoto Institute of Technology, Kyoto, Japan

[2] Faculty of Informatics, The University of Fukuchiyama, Kyoto, Japan

*** Correspondence:**

Noriaki Kuwahara

nkuwahar@kit.ac.jp

Content

**Supplementary questionnaires:**

**Supplementary 1. Photos questionnaire**(Japanese version) for investigating the personal preferences of older people for photos Page 3-44

1.Would you like to talk about this picture?

A. Like 2.No feeling 3.Dislike

2. Do you know what this photo is about?

1. Know 2. Don't know, but interested

3.Don't know, not interested

| |
|---|
| 1. この写真のお話しをしたいですか? |
| ☐好き ☐感じない ☐嫌い |
| 2. この写真の内容を知っていますか? |
| ☐知っている ☐知らないが興味がある |
| ☐知らない、興味がない |

**Supplementary 2. 写真に関する対話後の評価アンケート**(Japanese version) older people rating for their emotion after the conversation Page 45

**Supplementary 3. Self-assessment Form**(English version) Page 45

**Supplementary 4. 脳波取得装置の快適性に関するアンケート**(Japanese version) Page 46

**Supplementary 5. Ultracortex Mark IV Wearable Comfort Evaluation**(English version) Page 46

# 写真調査

この調査は、**2** 世代間の会話に適した資料を調査することです。

年齢 ： ___________

☐男性 ☐女性

若い世代の方は、次のページからの写真で高齢者の方とお話ししたいと思いますか。
また、年齢の高い世代の方は、次のページからの写真で若い世代の方とお話ししたいと思いますか

# 昭和暮らし

**1.アルミ洗濯バサミ**

軽く、薄く、弱々しく手にとればはかない

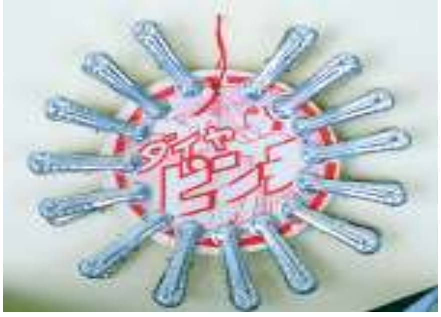

- この写真のお話しをしたいですか?

☐好き　☐感じない　☐嫌い

- この写真の内容を知っていますか?

☐知っている　☐知らないが興味がある

☐知らない、興味がない

**2.いずみ**

炊きたてご飯をおひつごと保温

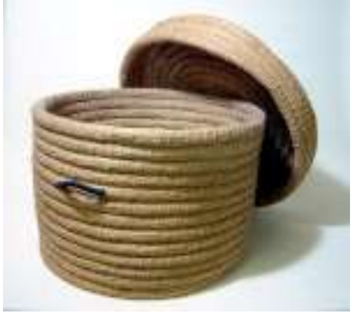

- この写真のお話しをしたいですか?

☐好き　☐感じない　☐嫌い

- この写真の内容を知っていますか?

☐知っている　☐知らないが興味がある

☐知らない、興味がない

**3.買い物かご**

財布入れ八百屋へ…懐かしい昭和の情景

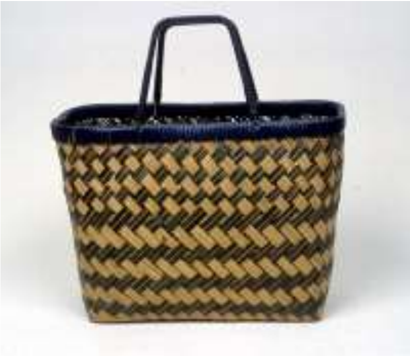

- この写真のお話しをしたいですか?

☐好き　☐感じない　☐嫌い

- この写真の内容を知っていますか?

☐知っている　☐知らないが興味がある

☐知らない、興味がない

**4.薬箱**

江戸時代から始まった 家庭に常備する置き薬

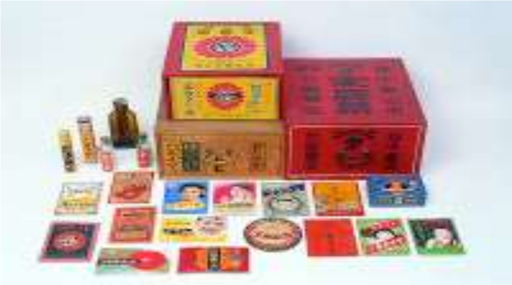

- この写真のお話しをしたいですか?

☐好き　☐感じない　☐嫌い

- この写真の内容を知っていますか?

☐知っている　☐知らないが興味がある

☐知らない、興味がない

**5.伸子**

着物を洗う作業が家庭でも日常的に

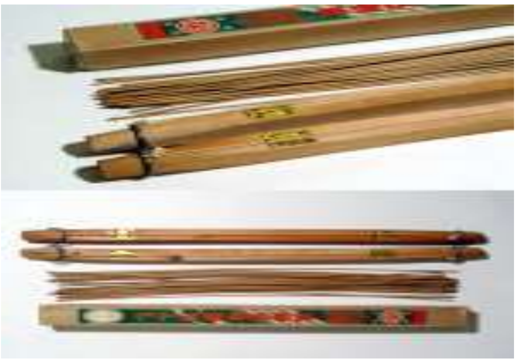

- この写真のお話しをしたいですか?

☐好き　☐感じない　☐嫌い

- この写真の内容を知っていますか?

☐知っている　☐知らないが興味がある

☐知らない、興味がない

**6.洗濯板**

今でも汚れ物に重宝

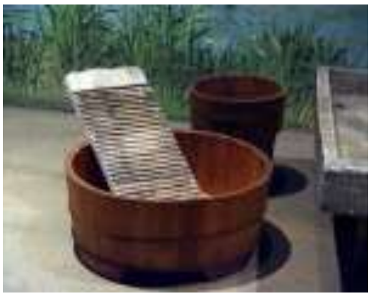

- この写真のお話しをしたいですか?

☐好き　☐感じない　☐嫌い

- この写真の内容を知っていますか?

☐知っている　☐知らないが興味がある

☐知らない、興味がない

**7.黒電話**

時間が必要『ダイヤル回す』

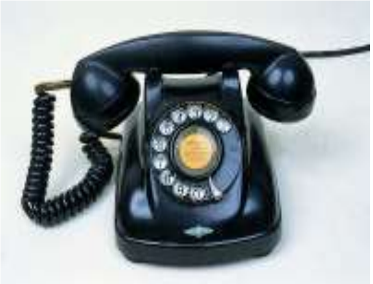

- この写真のお話しをしたいですか?

☐好き　☐感じない　☐嫌い

- この写真の内容を知っていますか?

☐知っている　☐知らないが興味がある

☐知らない、興味がない

**8.ハエたたき**

たたく部分は金網　重み利用しバシッ

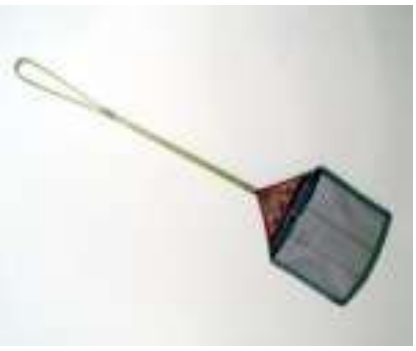

- この写真のお話しをしたいですか?

☐好き　☐感じない　☐嫌い

- この写真の内容を知っていますか?

☐知っている　☐知らないが興味がある

☐知らない、興味がない

**9.火熨斗（ひのし）**

火鉢から取り出し　炭で和式アイロン

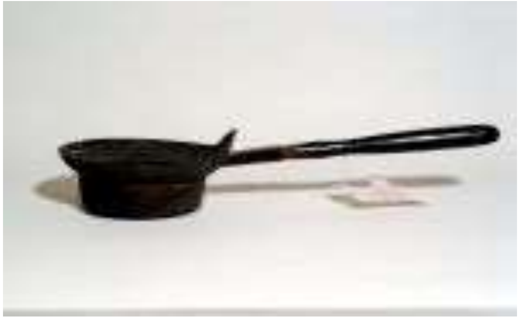

- この写真のお話しをしたいですか?

☐好き　☐感じない　☐嫌い

- この写真の内容を知っていますか?

☐知っている　☐知らないが興味がある

☐知らない、興味がない

**10.噴霧器**

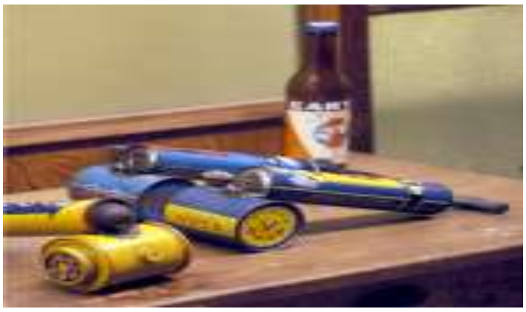

- この写真のお話しをしたいですか?

☐好き　☐感じない　☐嫌い

- この写真の内容を知っていますか?

☐知っている　☐知らないが興味がある

☐知らない、興味がない

**11.まな板**

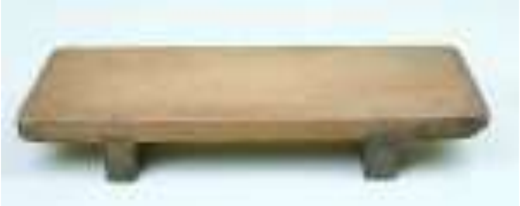

- この写真のお話しをしたいですか?

☐好き　☐感じない　☐嫌い

- この写真の内容を知っていますか?

☐知っている　☐知らないが興味がある

☐知らない、興味がない

**12.手洗い器**

“水の節約”おおいに貢献

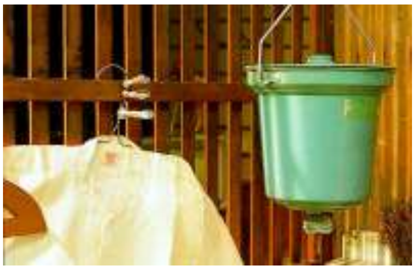

● この写真のお話しをしたいですか?

☐好き ☐感じない ☐嫌い

● この写真の内容を知っていますか?

☐知っている ☐知らないが興味がある

☐知らない、興味がない

**13. 箱ずし**

郷土の味を漬けたお祭りの日の食事

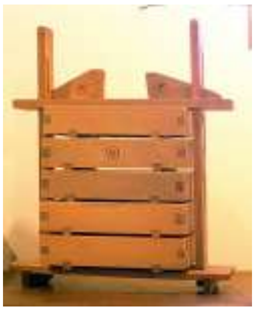

● この写真のお話しをしたいですか?

☐好き ☐感じない ☐嫌い

● この写真の内容を知っていますか?

☐知っている ☐知らないが興味がある

☐知らない、興味がない

**14.真空管**

懐かしいオレンジ光ラジオ、テレビで活躍

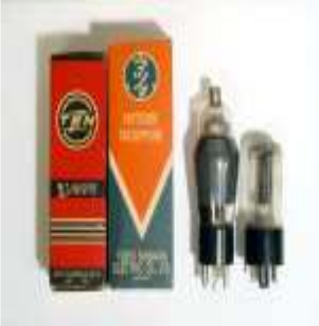

● この写真のお話しをしたいですか?

☐好き ☐感じない ☐嫌い

● この写真の内容を知っていますか?

☐知っている ☐知らないが興味がある

☐知らない、興味がない

**15.乾電池**

モーターおもちゃの陰の主役

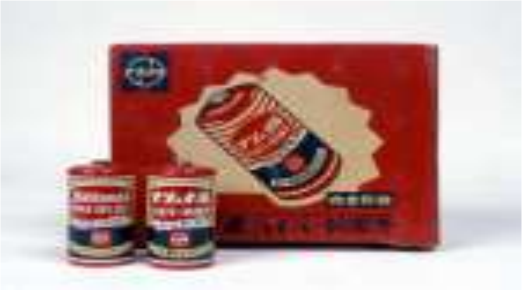

● この写真のお話しをしたいですか?

☐好き ☐感じない ☐嫌い

● この写真の内容を知っていますか?

☐知っている ☐知らないが興味がある

☐知らない、興味がない

**16.応接間**

現代の生活様式の原点

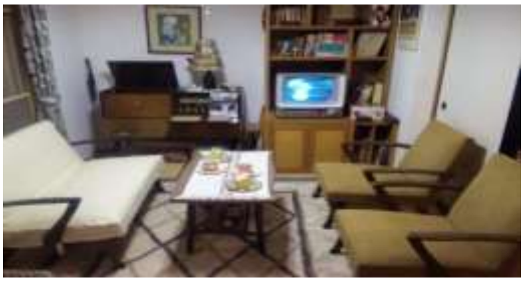

● この写真のお話しをしたいですか?

☐好き ☐感じない ☐嫌い

● この写真の内容を知っていますか?

☐知っている ☐知らないが興味がある

☐知らない、興味がない

**17. 8 ミリフィルム**

よみがえる思い出　保存の動き活発化

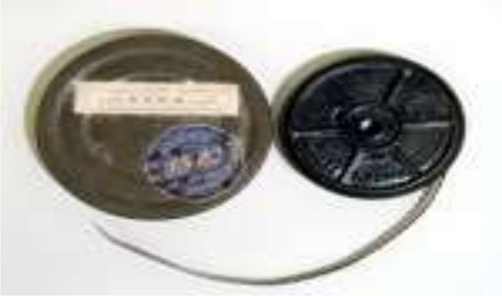

● この写真のお話しをしたいですか?
☐好き ☐感じない ☐嫌い
● この写真の内容を知っていますか?
☐知っている ☐知らないが興味がある
☐知らない、興味がない

**18. 歯磨き**

粉末や練り、瓶入り 昭和の日常を伝える

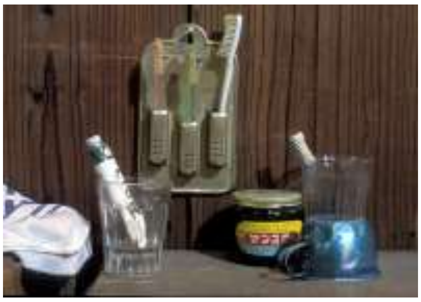

● この写真のお話しをしたいですか?
☐好き ☐感じない ☐嫌い
● この写真の内容を知っていますか?
☐知っている ☐知らないが興味がある
☐知らない、興味がない

**19.** ペナント

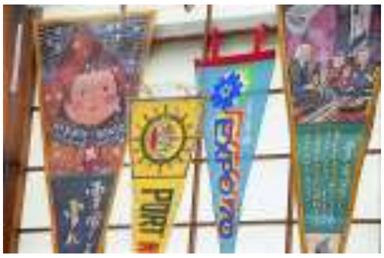

● この写真のお話しをしたいですか?
☐好き ☐感じない ☐嫌い
● この写真の内容を知っていますか?
☐知っている ☐知らないが興味がある
☐知らない、興味がない

**20.** キッチン

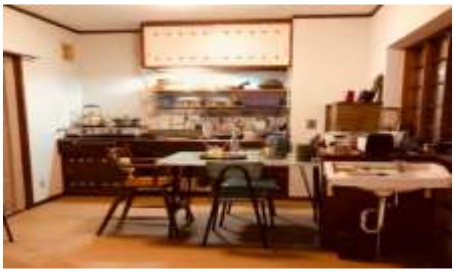

● この写真のお話しをしたいですか?
☐好き ☐感じない ☐嫌い
● この写真の内容を知っていますか?
☐知っている ☐知らないが興味がある
☐知らない、興味がない

21.マイカー時代

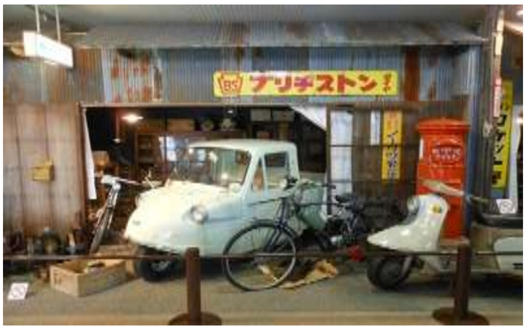

● この写真のお話しをしたいですか?
☐好き ☐感じない ☐嫌い
● この写真の内容を知っていますか?
☐知っている ☐知らないが興味がある
☐知らない、興味がない

**22.**ミカン箱

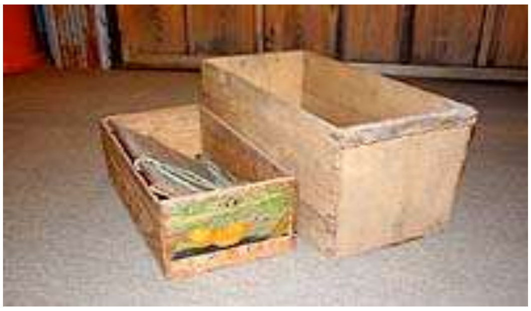

● この写真のお話しをしたいですか?
☐好き ☐感じない ☐嫌い
● この写真の内容を知っていますか?
☐知っている ☐知らないが興味がある
☐知らない、興味がない

23.保温･保冷ジャー

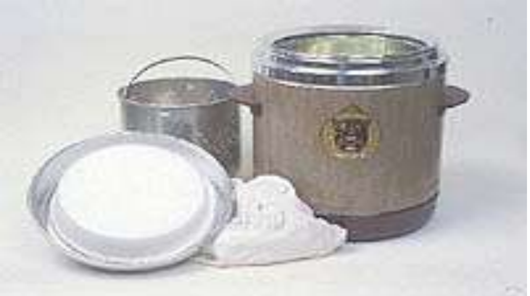

● この写真のお話しをしたいですか?

□好き　□感じない　□嫌い

● この写真の内容を知っていますか?

□知っている　□知らないが興味がある

□知らない、興味がない

**24.手回し洗濯器**

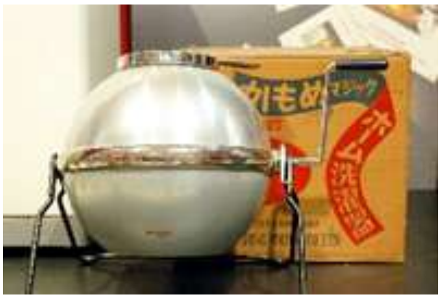

● この写真のお話しをしたいですか?

□好き　□感じない　□嫌い

● この写真の内容を知っていますか?

□知っている　□知らないが興味がある

□知らない、興味がない

**25.ほうき**

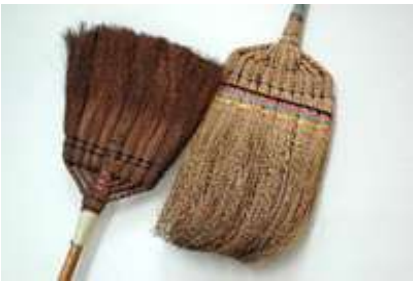

● この写真のお話しをしたいですか?

□好き　□感じない　□嫌い

● この写真の内容を知っていますか?

□知っている　□知らないが興味がある

□知らない、興味がない

**26.型紙**

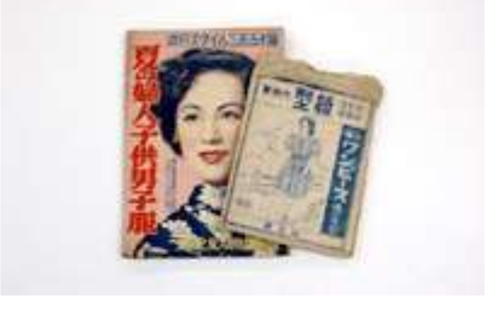

● この写真のお話しをしたいですか?

□好き　□感じない　□嫌い

● この写真の内容を知っていますか?

□知っている　□知らないが興味がある

□知らない、興味がない

**27.オブラート**

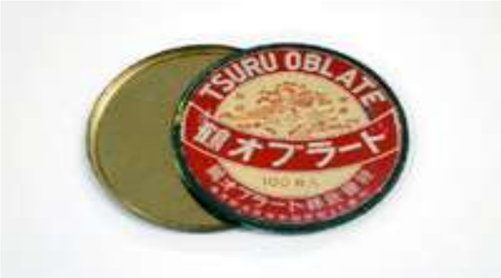


● この写真のお話しをしたいですか?

□好き　□感じない　□嫌い

● この写真の内容を知っていますか?

□知っている　□知らないが興味がある

□知らない、興味がない

**28.タワー**

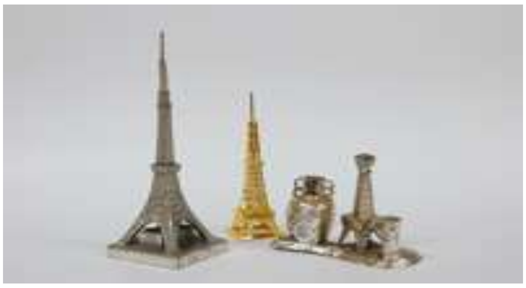

● この写真のお話しをしたいですか?

□好き　□感じない　□嫌い

● この写真の内容を知っていますか?

□知っている　□知らないが興味がある

□知らない、興味がない

**29.おしろい粉**

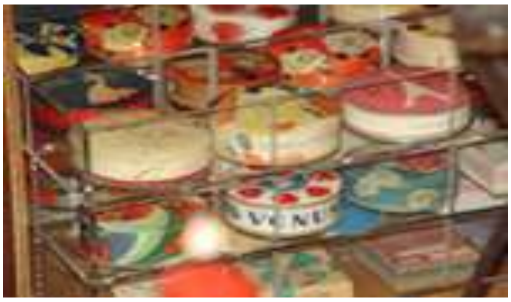

● この写真のお話しをしたいですか?

☐好き　　　☐感じない　　　☐嫌い

● この写真の内容を知っていますか?

☐知っている　☐知らないが興味がある

☐知らない、興味がない

**30.コンパクトミラー**

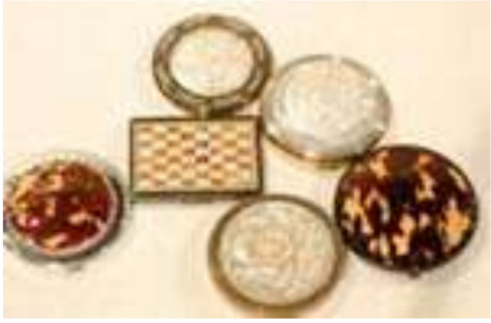

● この写真のお話しをしたいですか?

☐好き　　　☐感じない　　　☐嫌い

● この写真の内容を知っていますか?

☐知っている　☐知らないが興味がある

☐知らない、興味がない

**31.ねじしめ錠**

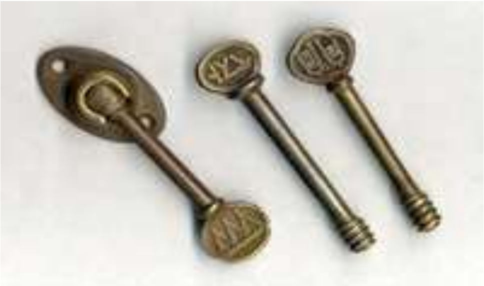

● この写真のお話しをしたいですか?

☐好き　　　☐感じない　　　☐嫌い

● この写真の内容を知っていますか?

☐知っている　☐知らないが興味がある

☐知らない、興味がない

**32.ラーメンどんぶり**

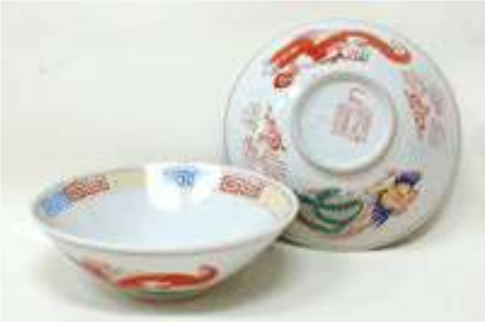

● この写真のお話しをしたいですか?

☐好き　　　☐感じない　　　☐嫌い

● この写真の内容を知っていますか?

☐知っている　☐知らないが興味がある

☐知らない、興味がない

**33.糸巻き**

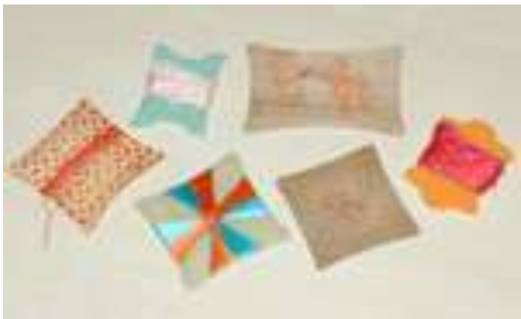

● この写真のお話しをしたいですか?

☐好き　　　☐感じない　　　☐嫌い

● この写真の内容を知っていますか?

☐知っている　☐知らないが興味がある

☐知らない、興味がない

**34.バリカン**

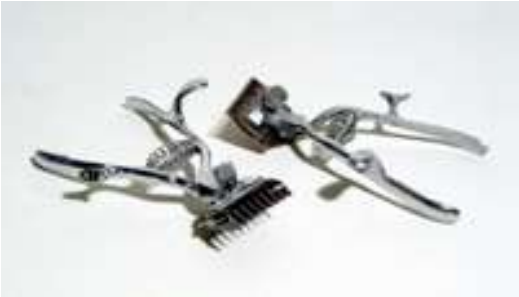

● この写真のお話しをしたいですか?

☐好き　　　☐感じない　　　☐嫌い

● この写真の内容を知っていますか?

☐知っている　☐知らないが興味がある

☐知らない、興味がない

**35.ジュラルミン**

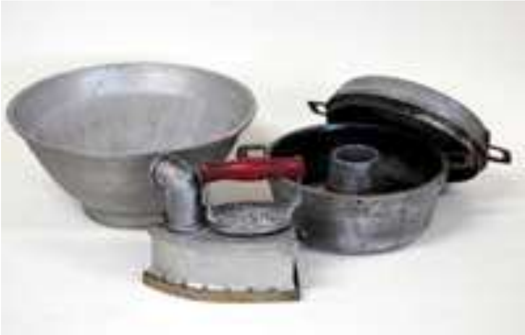

● この写真のお話しをしたいですか?

☐好き ☐感じない ☐嫌い

● この写真の内容を知っていますか?

☐知っている ☐知らないが興味がある

☐知らない、興味がない

**36.防虫剤の袋**

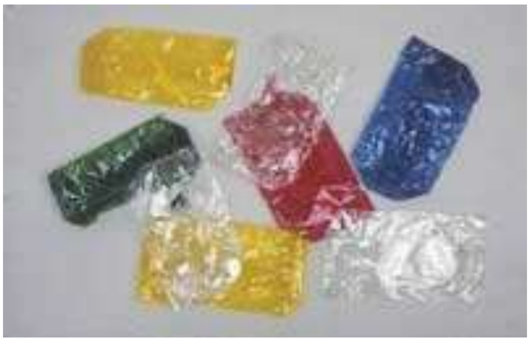

● この写真のお話しをしたいですか?

☐好き ☐感じない ☐嫌い

● この写真の内容を知っていますか?

☐知っている ☐知らないが興味がある

☐知らない、興味がない

**37.カーテンの留め金具**

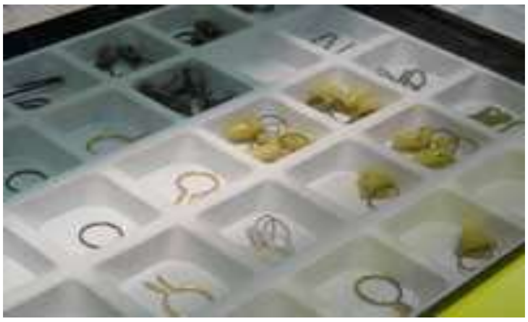

● この写真のお話しをしたいですか?

☐好き ☐感じない ☐嫌い

● この写真の内容を知っていますか?

☐知っている ☐知らないが興味がある

☐知らない、興味がない

**38.有線放送**

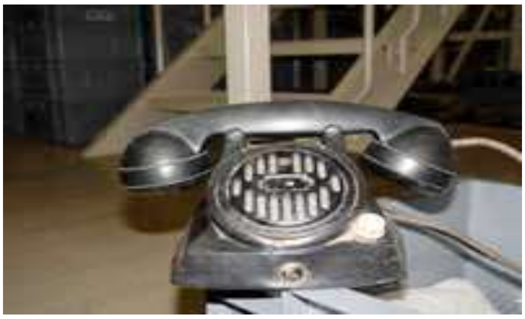

● この写真のお話しをしたいですか?

☐好き ☐感じない ☐嫌い

● この写真の内容を知っていますか?

☐知っている ☐知らないが興味がある

☐知らない、興味がない

**39.ヘチマたわし**

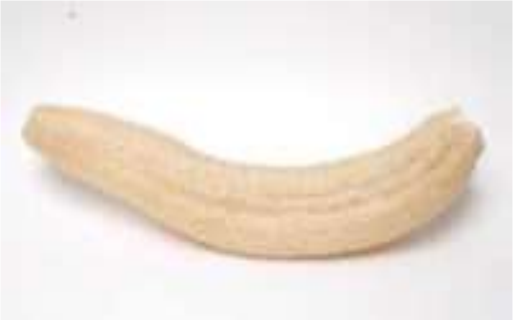

● この写真のお話しをしたいですか?

☐好き ☐感じない ☐嫌い

● この写真の内容を知っていますか?

☐知っている ☐知らないが興味がある

☐知らない、興味がない

**40.鋳掛（いかけ）**

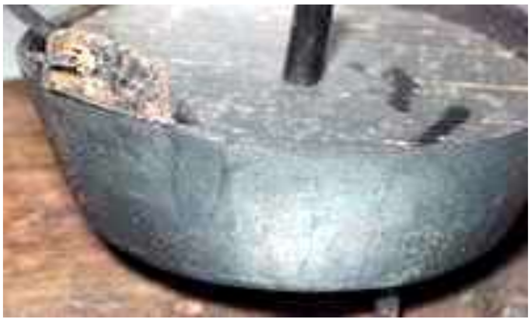

● この写真のお話しをしたいですか?

☐好き ☐感じない ☐嫌い

● この写真の内容を知っていますか?

☐知っている ☐知らないが興味がある

☐知らない、興味がない

**41.蠅帳（はいちょう）** ハエから食べ物守る 保管場所として重宝

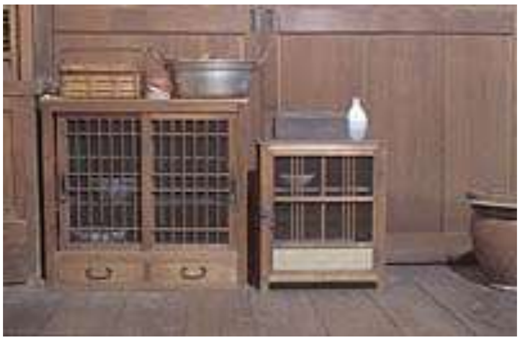

● この写真のお話しをしたいですか?
☐好き　☐感じない　☐嫌い
● この写真の内容を知っていますか?
☐知っている　☐知らないが興味がある
☐知らない、興味がない

**42.水枕**

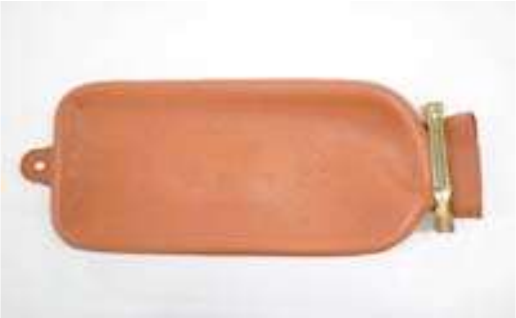

● この写真のお話しをしたいですか?
☐好き　☐感じない　☐嫌い
● この写真の内容を知っていますか?
☐知っている　☐知らないが興味がある
☐知らない、興味がない

**43.ワンピース**

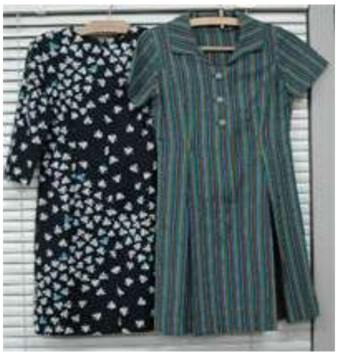

● この写真のお話しをしたいですか?
☐好き　☐感じない　☐嫌い
● この写真の内容を知っていますか?
☐知っている　☐知らないが興味がある
☐知らない、興味がない

**44.写真用三角コーナー**

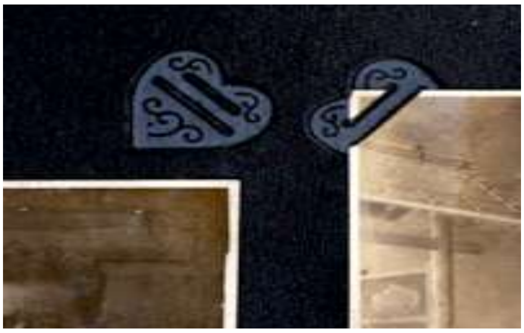

● この写真のお話しをしたいですか?
☐好き　☐感じない　☐嫌い
● この写真の内容を知っていますか?
☐知っている　☐知らないが興味がある
☐知らない、興味がない

**45.へちま**

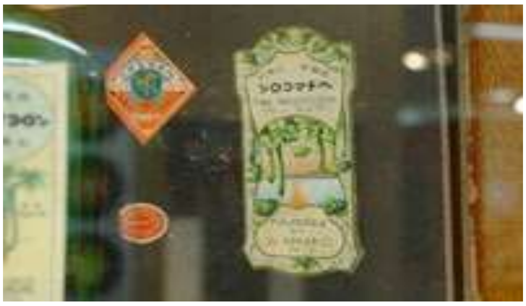

● この写真のお話しをしたいですか?
☐好き　☐感じない　☐嫌い
● この写真の内容を知っていますか?
☐知っている　☐知らないが興味がある
☐知らない、興味がない

**46.くじらのひげ**

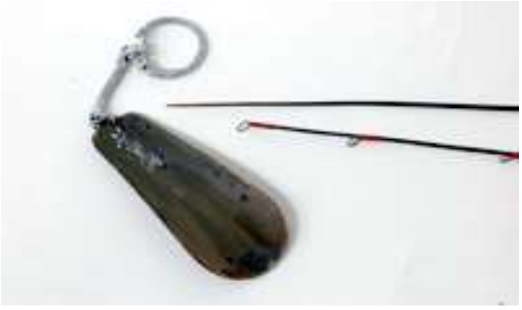

● この写真のお話しをしたいですか?
☐好き　☐感じない　☐嫌い
● この写真の内容を知っていますか?
☐知っている　☐知らないが興味がある
☐知らない、興味がない

**47.廃物利用五百種**

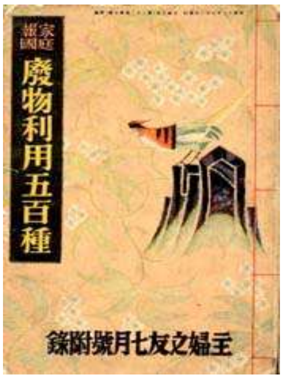


- この写真のお話しをしたいですか?

☐好き　　☐感じない　　☐嫌い

- この写真の内容を知っていますか?

☐知っている　☐知らないが興味がある

☐知らない、興味がない

**48.扇風機**

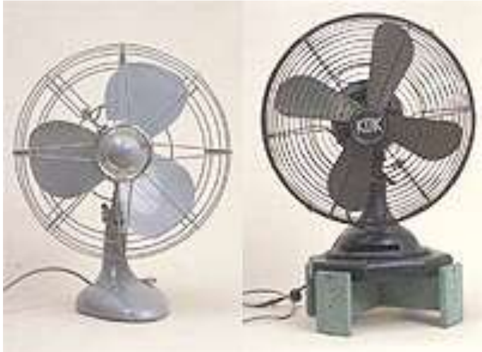

- この写真のお話しをしたいですか?

☐好き　　☐感じない　　☐嫌い

- この写真の内容を知っていますか?

☐知っている　☐知らないが興味がある

☐知らない、興味がない

**49.アイスキャンデー**

夏の暑い日にチリンチリン

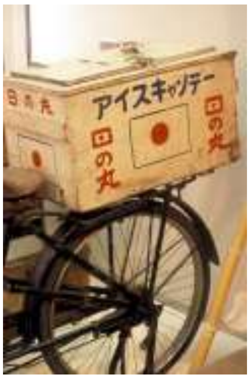


- この写真のお話しをしたいですか?

☐好き　　☐感じない　　☐嫌い

- この写真の内容を知っていますか?

☐知っている　☐知らないが興味がある

☐知らない、興味がない

**50.アイスクリーム保冷箱**

王、長島、星野… ヒーローの足跡

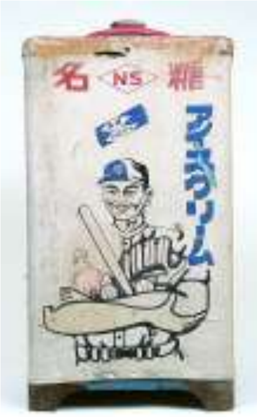


- この写真のお話しをしたいですか?

☐好き　　☐感じない　　☐嫌い

- この写真の内容を知っていますか?

☐知っている　☐知らないが興味がある

☐知らない、興味がない

**51.かき氷機**

冷たくガリガリ　夏の郷愁を誘う

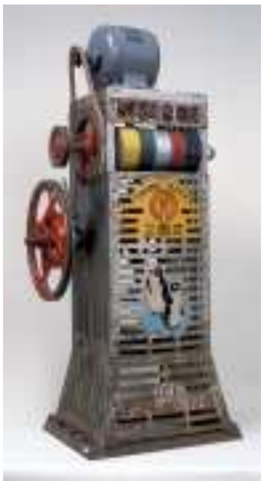

- この写真のお話しをしたいですか?

☐好き　　☐感じない　　☐嫌い

- この写真の内容を知っていますか?

☐知っている　☐知らないが興味がある

☐知らない、興味がない

**52.蚊帳**

寝苦しい夏の夜　涼しい風を誘う

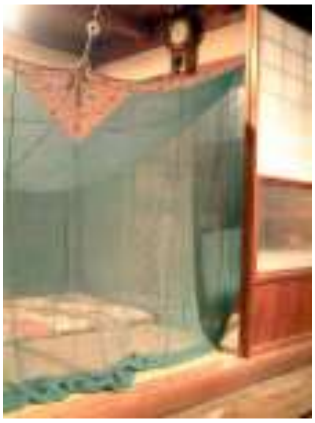

● この写真のお話しをしたいですか?

☐好き　☐感じない　☐嫌い

● この写真の内容を知っていますか?

☐知っている　☐知らないが興味がある

☐知らない、興味がない

**53.海辺のお土産**

夏休みを思い出す

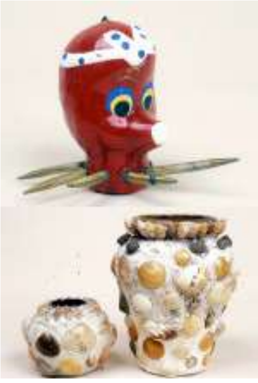

● この写真のお話しをしたいですか?

☐好き　☐感じない　☐嫌い

● この写真の内容を知っていますか?

☐知っている　☐知らないが興味がある

☐知らない、興味がない

**54.かぜ薬**

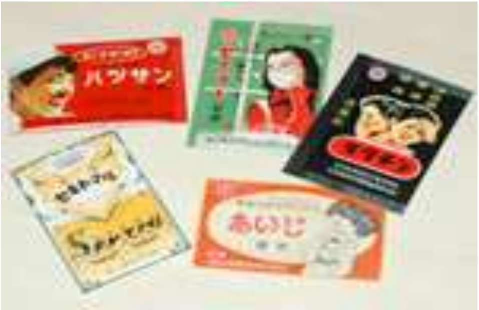


● この写真のお話しをしたいですか?

☐好き　☐感じない　☐嫌い

● この写真の内容を知っていますか?

☐知っている　☐知らないが興味がある

☐知らない、興味がない

**55.あんか**

控えめにホカホカ　朱色が郷愁を誘う

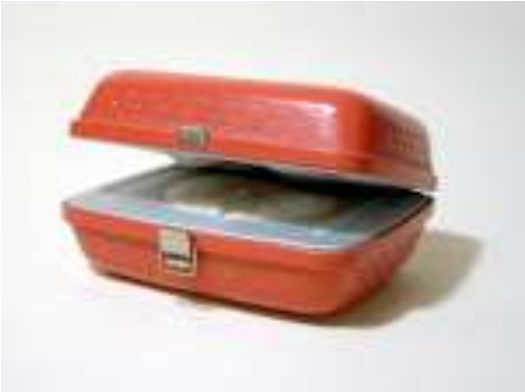

● この写真のお話しをしたいですか?

☐好き　☐感じない　☐嫌い

● この写真の内容を知っていますか?

☐知っている　☐知らないが興味がある

☐知らない、興味がない

**56.湯たんぽ**

今は『チン』するタイプ

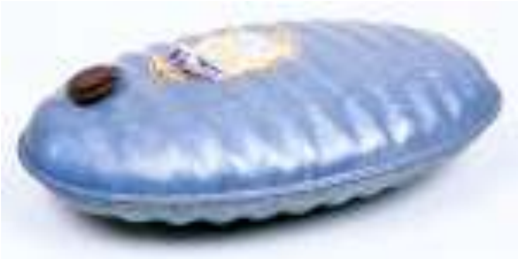

● この写真のお話しをしたいですか?

☐好き　☐感じない　☐嫌い

● この写真の内容を知っていますか?

□知っている　□知らないが興味がある
□知らない、興味がない

**57.クリスマスの包装紙**
破り捨てられるが懐かしい思い残す

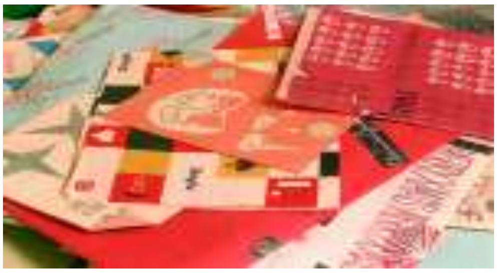

- この写真のお話しをしたいですか?

□好き　　□感じない　　□嫌い

- この写真の内容を知っていますか?

□知っている　□知らないが興味がある
□知らない、興味がない

**58.歩行器**

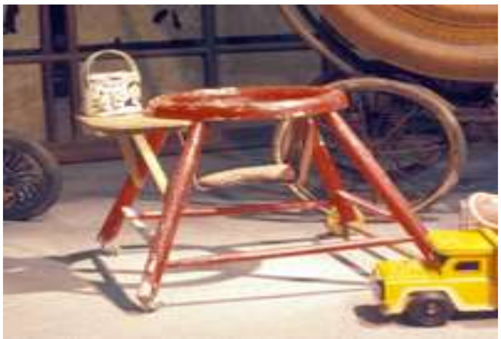

- この写真のお話しをしたいですか?

□好き　　□感じない　　□嫌い

- この写真の内容を知っていますか?

□知っている　□知らないが興味がある
□知らない、興味がない

**59.ちゃん用蚊帳（かや）**

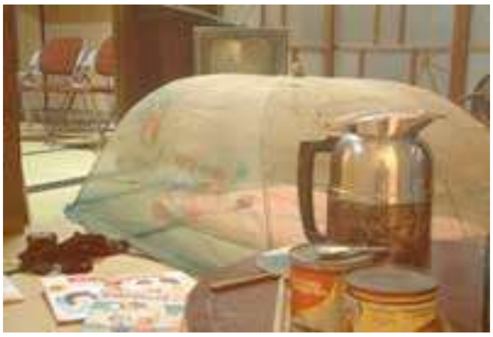

- この写真のお話しをしたいですか?

□好き　　□感じない　　□嫌い

- この写真の内容を知っていますか?

□知っている　□知らないが興味がある
□知らない、興味がない

**60.乳母車**
縁起物のデザインで大きなサイズが特徴

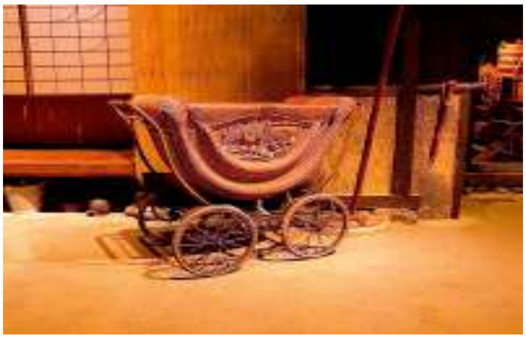

- この写真のお話しをしたいですか?

□好き　　□感じない　　□嫌い

- この写真の内容を知っていますか?

□知っている　□知らないが興味がある
□知らない、興味がない

**61.おまる**
使った本人よりも親の方が思い出に

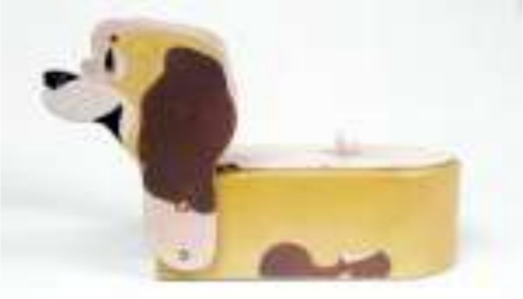

- この写真のお話しをしたいですか?

□好き　　□感じない　　□嫌い

- この写真の内容を知っていますか?

□知っている　□知らないが興味がある
□知らない、興味がない

**62.カタカタ**
一緒に歩き始めた品子にも親にも思い出

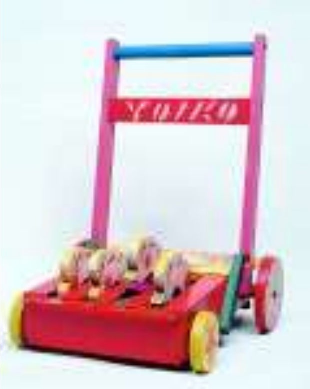

● この写真のお話しをしたいですか?
☐好き　　☐感じない　　☐嫌い
● この写真の内容を知っていますか?
☐知っている　☐知らないが興味がある
☐知らない、興味がない

**63.乳幼児用はかり**
ゾウの背中に乗って成長確認

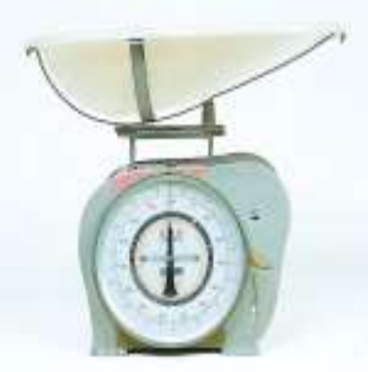

● この写真のお話しをしたいですか?
☐好き　　☐感じない　　☐嫌い
● この写真の内容を知っていますか?
☐知っている　☐知らないが興味がある
☐知らない、興味がない

**64.木馬**
親の思いやりが伝わってくる

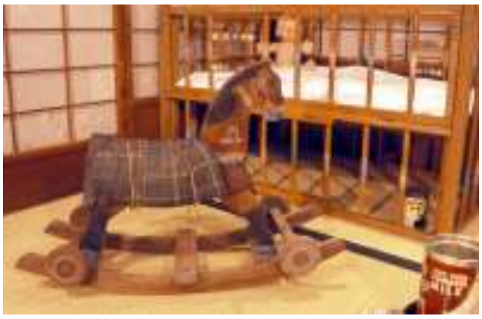

● この写真のお話しをしたいですか?
☐好き　　☐感じない　　☐嫌い
● この写真の内容を知っていますか?
☐知っている　☐知らないが興味がある
☐知らない、興味がない

**65.地球儀**

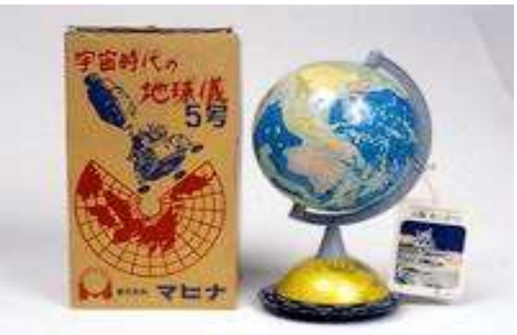


● この写真のお話しをしたいですか?
☐好き　　☐感じない　　☐嫌い
● この写真の内容を知っていますか?
☐知っている　☐知らないが興味がある
☐知らない、興味がない

**66.鉛筆削り**

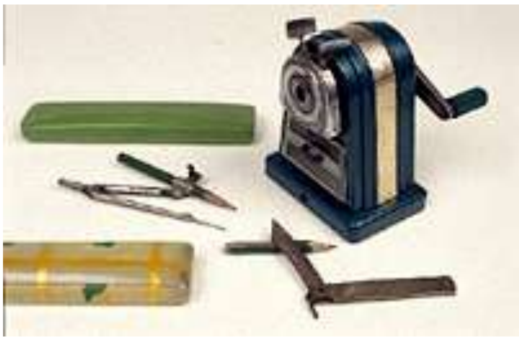

● この写真のお話しをしたいですか?
☐好き　　☐感じない　　☐嫌い
● この写真の内容を知っていますか?
☐知っている　☐知らないが興味がある
☐知らない、興味がない

**67.裁縫箱**

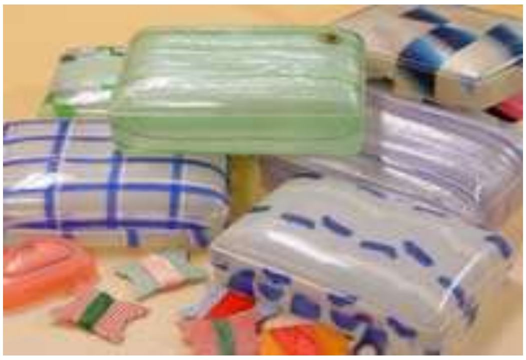

● この写真のお話しをしたいですか?
□好き　□感じない　□嫌い
● この写真の内容を知っていますか?
□知っている　□知らないが興味がある
□知らない、興味がない

**68.運動足袋**

短距離走など運動会で活躍

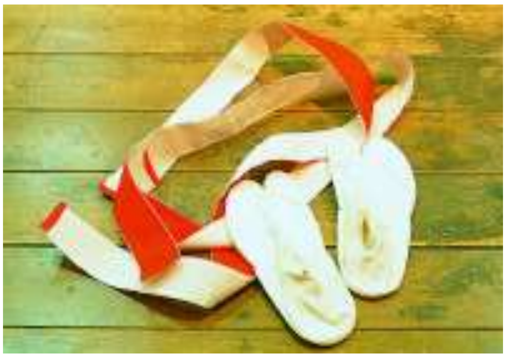

● この写真のお話しをしたいですか?
□好き　□感じない　□嫌い
● この写真の内容を知っていますか?
□知っている　□知らないが興味がある
□知らない、興味がない

**69.学生服**

かつては小学生から

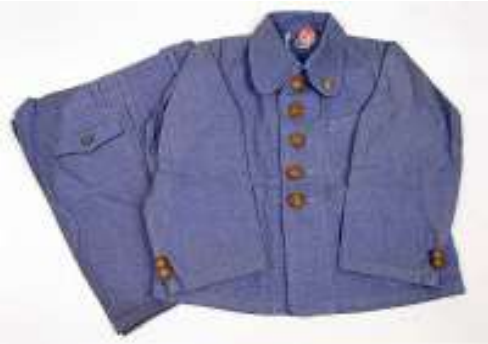

● この写真のお話しをしたいですか?
□好き　□感じない　□嫌い
● この写真の内容を知っていますか?
□知っている　□知らないが興味がある
□知らない、興味がない

**70.石盤と石筆**

書き消しが何度でもノート普及で消える

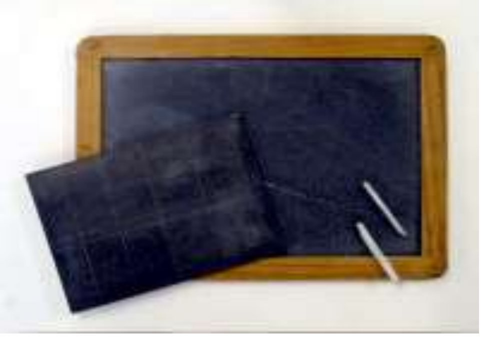

● この写真のお話しをしたいですか?
□好き　□感じない　□嫌い
● この写真の内容を知っていますか?
□知っている　□知らないが興味がある
□知らない、興味がない

**71.脱脂粉乳**

『あのにおい』思い出す容器

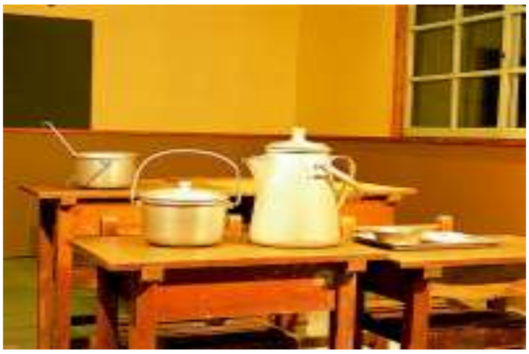

● この写真のお話しをしたいですか?
□好き　□感じない　□嫌い
● この写真の内容を知っていますか?
□知っている　□知らないが興味がある
□知らない、興味がない

**72.ランドセル**

見覚えある? “野球少年もの”

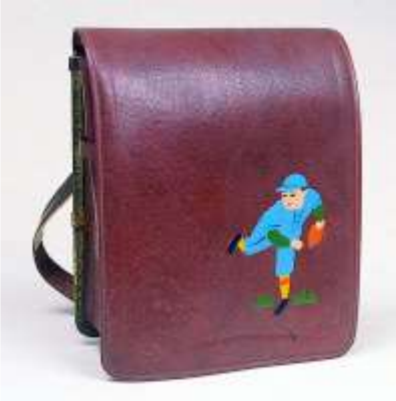

● この写真のお話しをしたいですか?
□好き　　□感じない　　□嫌い
● この写真の内容を知っていますか?
□知っている　□知らないが興味がある
□知らない、興味がない

**73.バリカン**
かつては小学生から

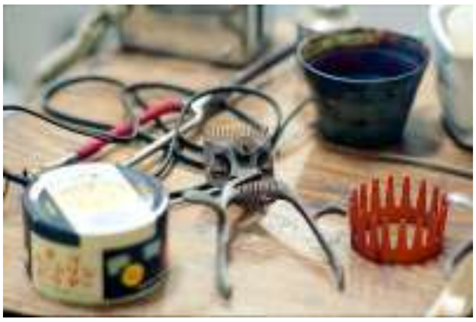

● この写真のお話しをしたいですか?
□好き　　□感じない　　□嫌い
● この写真の内容を知っていますか?
□知っている　□知らないが興味がある
□知らない、興味がない

**74.ブルマー**
運動会では大活躍　黒いちょうちん型

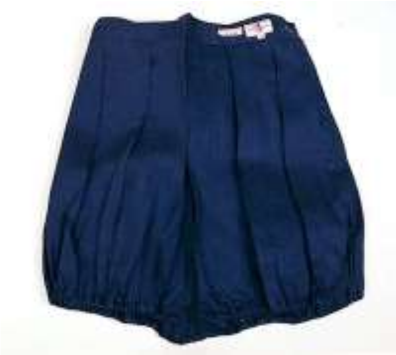

● この写真のお話しをしたいですか?
□好き　　□感じない　　□嫌い
● この写真の内容を知っていますか?
□知っている　□知らないが興味がある
□知らない、興味がない

**75.日光写真**

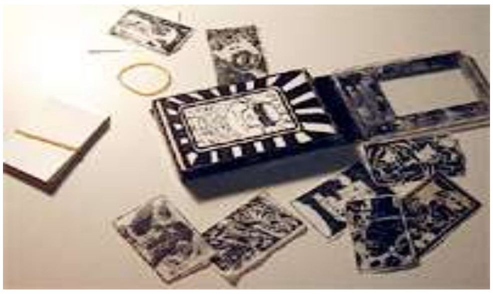

● この写真のお話しをしたいですか?
□好き　　□感じない　　□嫌い
● この写真の内容を知っていますか?
□知っている　□知らないが興味がある
□知らない、興味がない

**76.巻き玉鉄砲**

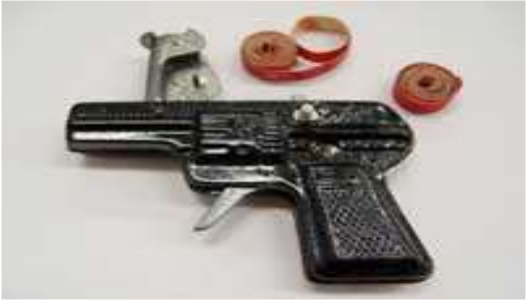

● この写真のお話しをしたいですか?
□好き　　□感じない　　□嫌い
● この写真の内容を知っていますか?
□知っている　□知らないが興味がある
□知らない、興味がない

**77.面子**

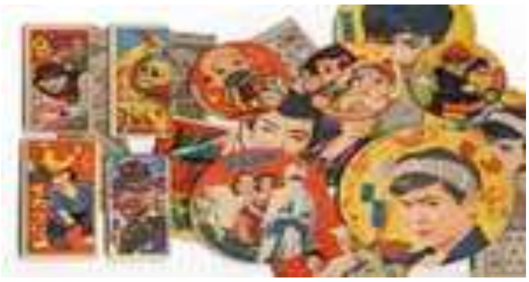

● この写真のお話しをしたいですか?
□好き　　□感じない　　□嫌い
● この写真の内容を知っていますか?
□知っている　□知らないが興味がある
□知らない、興味がない

**78.王冠**
コルクはがし“バッジ”に

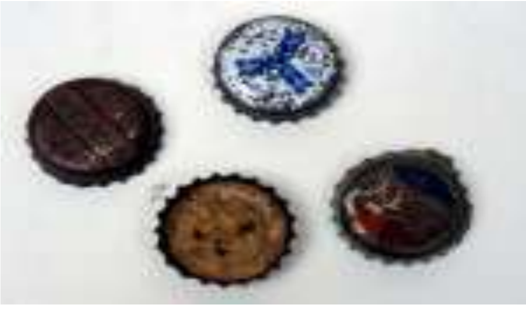

● この写真のお話しをしたいですか?
☐好き ☐感じない ☐嫌い
● この写真の内容を知っていますか?
☐知っている ☐知らないが興味がある
☐知らない、興味がない

**79.おまけ**
時代をミニチュア化商品本体より魅力的

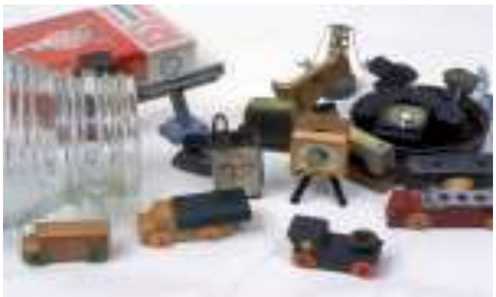

● この写真のお話しをしたいですか?
☐好き ☐感じない ☐嫌い
● この写真の内容を知っていますか?
☐知っている ☐知らないが興味がある
☐知らない、興味がない

**80.紙風船**
置き薬売りのおまけ時代を映す楽しい絵

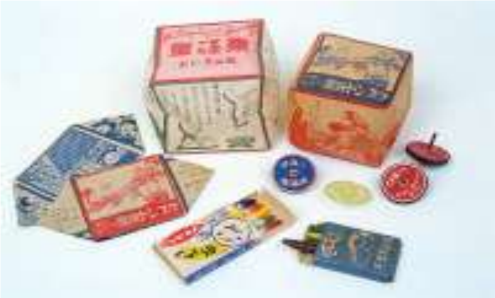

● この写真のお話しをしたいですか?
☐好き ☐感じない ☐嫌い
● この写真の内容を知っていますか?
☐知っている ☐知らないが興味がある
☐知らない、興味がない

**81.シャボン玉の道具**
液を蓄えるため パイプの先工夫

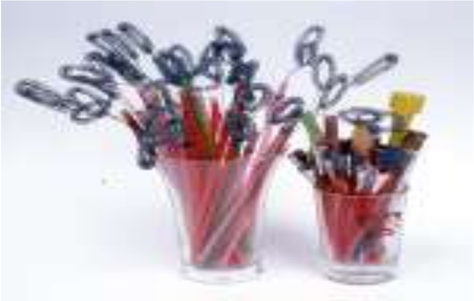

● この写真のお話しをしたいですか?
☐好き ☐感じない ☐嫌い
● この写真の内容を知っていますか?
☐知っている ☐知らないが興味がある
☐知らない、興味がない

**82.スケーター**
姿少し変え、流行繰り返す

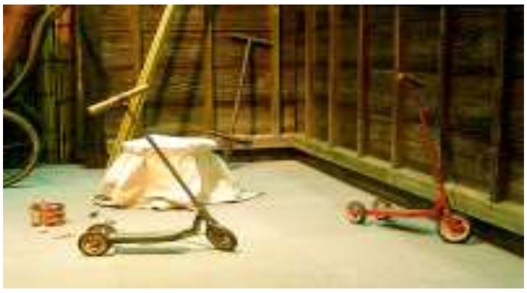

● この写真のお話しをしたいですか?
☐好き ☐感じない ☐嫌い
● この写真の内容を知っていますか?
☐知っている ☐知らないが興味がある
☐知らない、興味がない

**83.『セミカチ』**
遊び方は工夫でいくつも

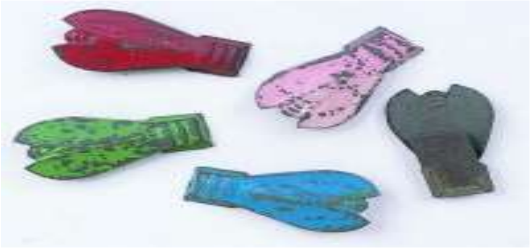

● この写真のお話しをしたいですか?
☐好き ☐感じない ☐嫌い
● この写真の内容を知っていますか?

□知っている　□知らないが興味がある
□知らない、興味がない

**84.セルロイド**
ノスタルジックな色材質には“はかなさ”

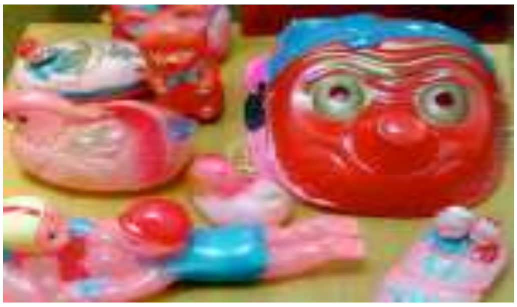

● この写真のお話しをしたいですか?
□好き　　□感じない　　□嫌い
● この写真の内容を知っていますか?
□知っている　□知らないが興味がある
□知らない、興味がない

**85.ソノシート**
雑誌がくるくる回る

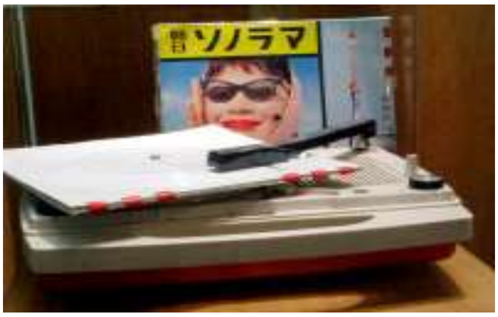


● この写真のお話しをしたいですか?
□好き　　□感じない　　□嫌い
● この写真の内容を知っていますか?
□知っている　□知らないが興味がある
□知らない、興味がない

**86.ちゃんばら**
時代劇にあこがれた少年ら

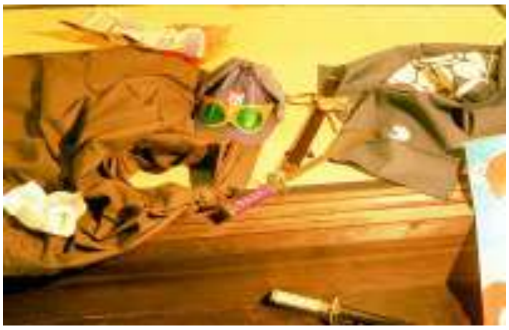

● この写真のお話しをしたいですか?
□好き　　□感じない　　□嫌い
● この写真の内容を知っていますか?
□知っている　□知らないが興味がある
□知らない、興味がない

**87.釣り具**
竹の竿や仕掛け巻き空き缶は宝箱に変身

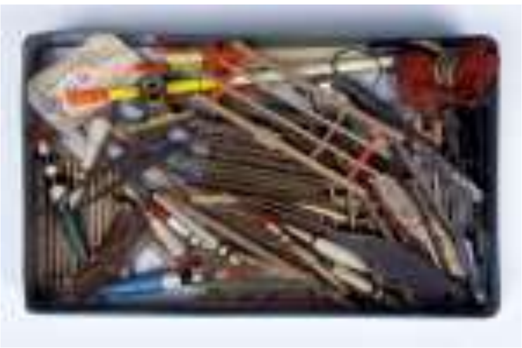

● この写真のお話しをしたいですか?
□好き　　□感じない　　□嫌い
● この写真の内容を知っていますか?
□知っている　□知らないが興味がある
□知らない、興味がない

**88. ブリキの自動車**
マイカー時代到来　波に乗るのも大変

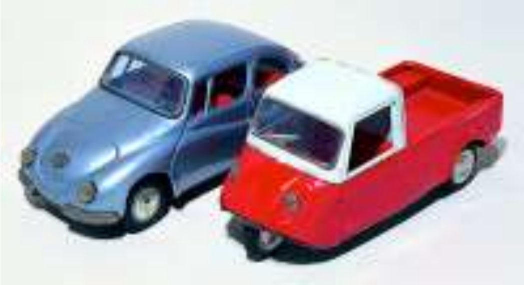

● この写真のお話しをしたいですか?
□好き　　□感じない　　□嫌い
● この写真の内容を知っていますか?

□知っている　□知らないが興味がある
□知らない、興味がない

**89.水遊び・行水**

冷たい井戸水くみブリキ玩具楽しむ

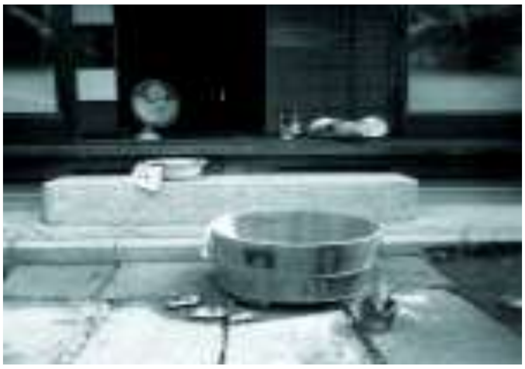

- この写真のお話しをしたいですか?

□好き　□感じない　□嫌い

- この写真の内容を知っていますか?

□知っている　□知らないが興味がある
□知らない、興味がない

**90.野球盤**

「消える魔球」など少年の心とらえる

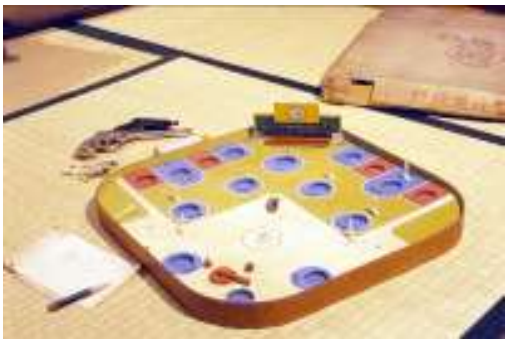

- この写真のお話しをしたいですか?

□好き　□感じない　□嫌い

- この写真の内容を知っていますか?

□知っている　□知らないが興味がある
□知らない、興味がない

**91.野球用具**

立派な市販品がなくても　手作りボールとバットで

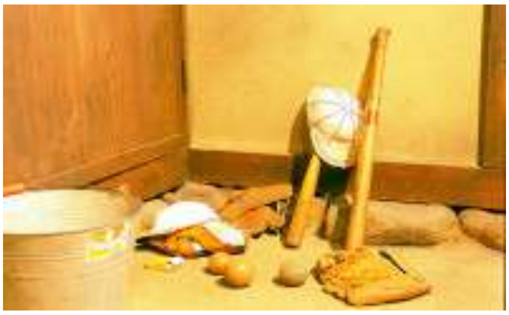

- この写真のお話しをしたいですか?

□好き　□感じない　□嫌い

- この写真の内容を知っていますか?

□知っている　□知らないが興味がある
□知らない、興味がない

**92.虫かご**

苦労して捕まえた一匹一匹に思い出

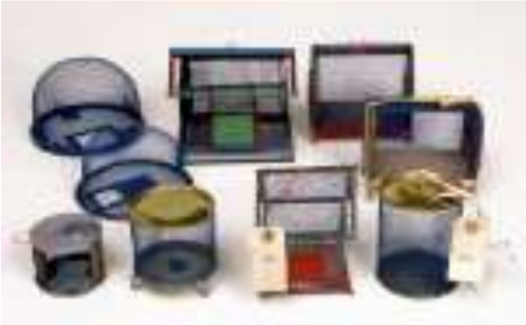

- この写真のお話しをしたいですか?

□好き　□感じない　□嫌い

- この写真の内容を知っていますか?

□知っている　□知らないが興味がある
□知らない、興味がない

**93.空箱**

子どもたちの『宝箱』ブリキ製は母も重宝

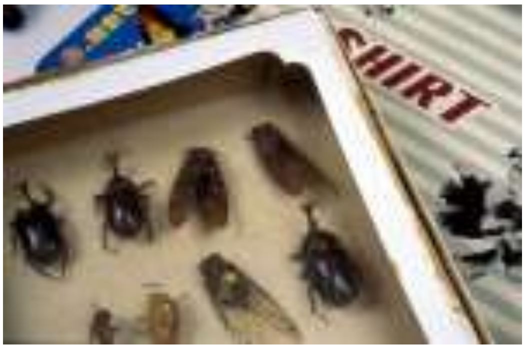

- この写真のお話しをしたいですか?

□好き　□感じない　□嫌い

- この写真の内容を知っていますか?

☐知っている　☐知らないが興味がある
☐知らない、興味がない

**94.自転車の空気入れ**

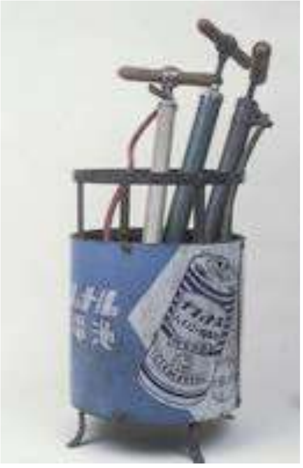

● この写真のお話しをしたいですか?
☐好き　☐感じない　☐嫌い
● この写真の内容を知っていますか?
☐知っている　☐知らないが興味がある
☐知らない、興味がない

**95.郵便ポスト**
円筒形はまだ健在　戦時中には陶器製も

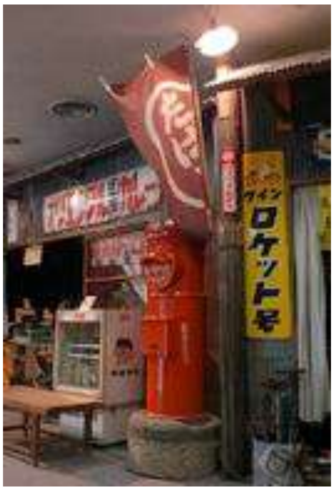

● この写真のお話しをしたいですか?
☐好き　☐感じない　☐嫌い
● この写真の内容を知っていますか?
☐知っている　☐知らないが興味がある
☐知らない、興味がない

**96.陸王**

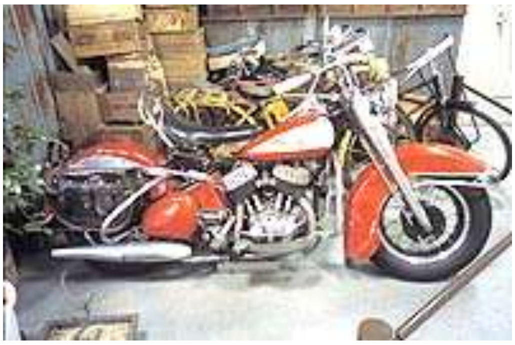

● この写真のお話しをしたいですか?
☐好き　☐感じない　☐嫌い
● この写真の内容を知っていますか?
☐知っている　☐知らないが興味がある
☐知らない、興味がない

**97.赤電話**

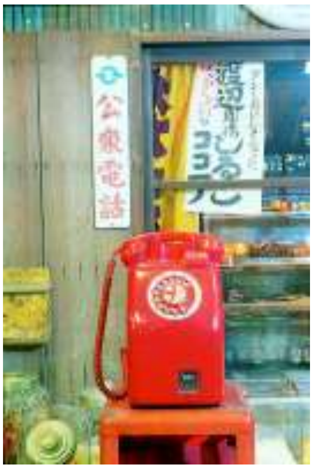


● この写真のお話しをしたいですか?
☐好き　☐感じない　☐嫌い
● この写真の内容を知っていますか?
☐知っている　☐知らないが興味がある
☐知らない、興味がない

**98.木製の縁台**
人と人とを結ぶ役割

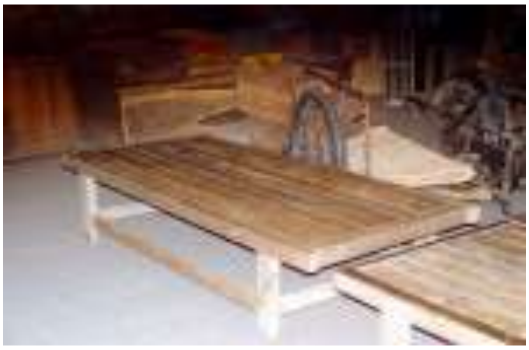

● この写真のお話しをしたいですか?
☐好き　　☐感じない　　☐嫌い
● この写真の内容を知っていますか?
☐知っている　☐知らないが興味がある
☐知らない、興味がない

**99.牛乳配達の自転車**
目を引く広告看板
荷台は大きく頑丈

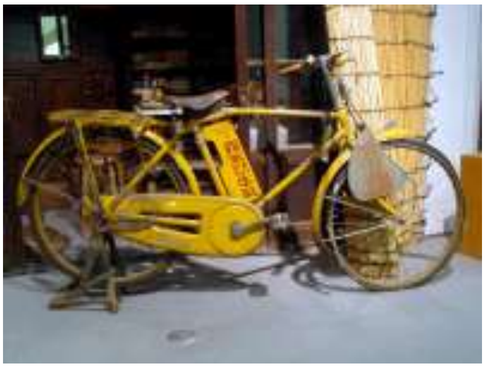

● この写真のお話しをしたいですか?
☐好き　　☐感じない　　☐嫌い
● この写真の内容を知っていますか?
☐知っている　☐知らないが興味がある
☐知らない、興味がない

**100.自転車の鑑札**
税金を支払って入手"自動車並み"の扱い

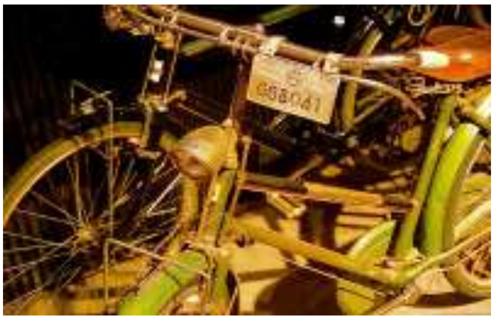

● この写真のお話しをしたいですか?
☐好き　　☐感じない　　☐嫌い
● この写真の内容を知っていますか?
☐知っている　☐知らないが興味がある
☐知らない、興味がない

**101.スクーター**
お手ごろな値段で昭和30年代に人気

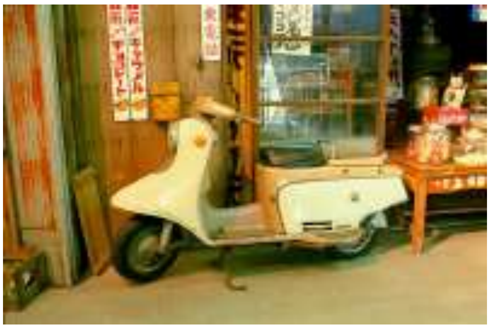

● この写真のお話しをしたいですか?
☐好き　　☐感じない　　☐嫌い
● この写真の内容を知っていますか?
☐知っている　☐知らないが興味がある
☐知らない、興味がない

**102.電信棒と街路灯スイッチ**
一本ずつ点灯して回る

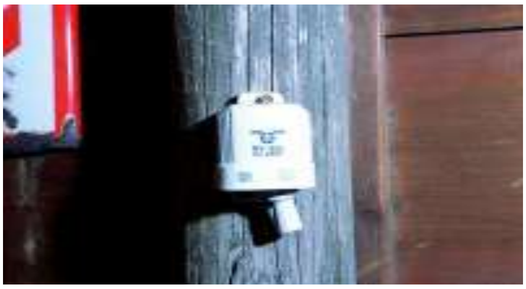

● この写真のお話しをしたいですか?
☐好き　　☐感じない　　☐嫌い
● この写真の内容を知っていますか?
☐知っている　☐知らないが興味がある
☐知らない、興味がない

**103.ほうろう看板**
昭和の風景を物語るさびに強く屋外向き

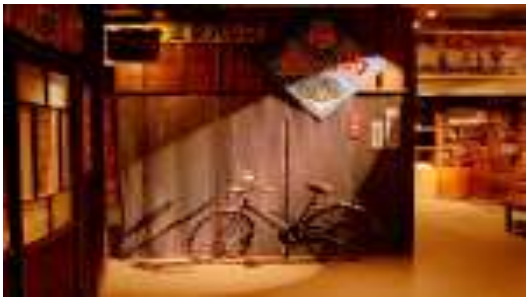

● この写真のお話しをしたいですか?
☐好き　　☐感じない　　☐嫌い
● この写真の内容を知っていますか?
☐知っている　☐知らないが興味がある
☐知らない、興味がない

**104.籾殻と卵**

産みたてをくるみ込む

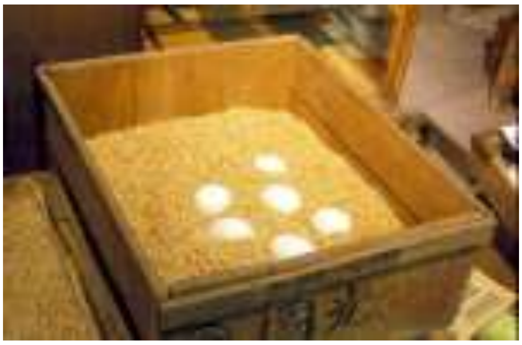

● この写真のお話しをしたいですか?

□好き　□感じない　□嫌い

● この写真の内容を知っていますか?

□知っている　□知らないが興味がある

□知らない、興味がない

**105.レジスター**

不作法さが残る計算機械

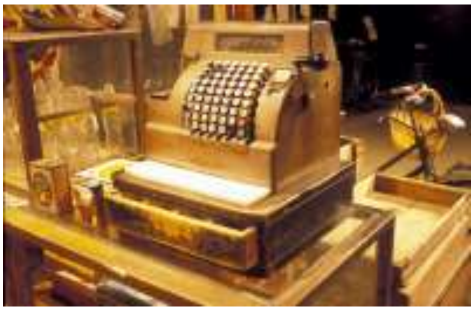

● この写真のお話しをしたいですか?

□好き　□感じない　□嫌い

● この写真の内容を知っていますか?

□知っている　□知らないが興味がある

□知らない、興味がない

**106.たばこケース**

分厚いガラス、タイル

昭和 30 年代の風物詩

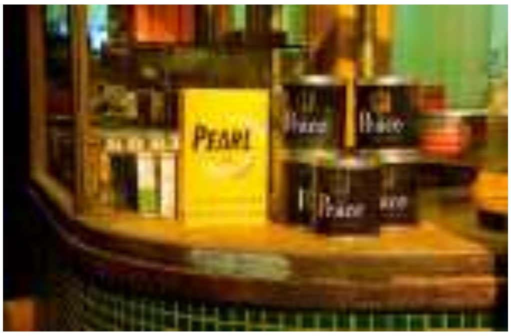


● この写真のお話しをしたいですか?

□好き　□感じない　□嫌い

● この写真の内容を知っていますか?

□知っている　□知らないが興味がある

□知らない、興味がない

**107.電気釜の内釜**

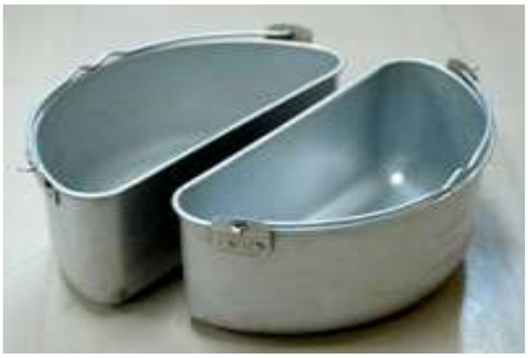

● この写真のお話しをしたいですか?

□好き　□感じない　□嫌い

● この写真の内容を知っていますか?

□知っている　□知らないが興味がある

□知らない、興味がない

**108.ヒューズ**

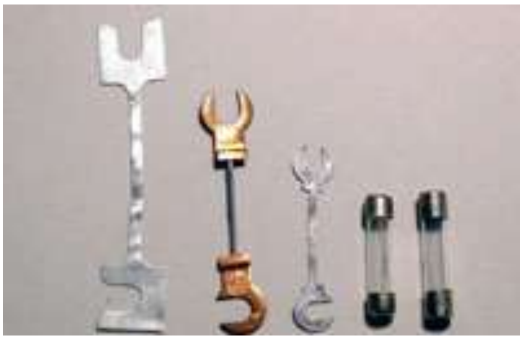

● この写真のお話しをしたいですか?

□好き　□感じない　□嫌い

● この写真の内容を知っていますか?

□知っている　□知らないが興味がある

□知らない、興味がない

**109.タイマー**

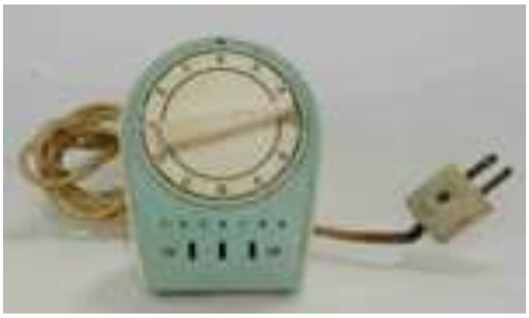

● この写真のお話しをしたいですか?

□好き　□感じない　□嫌い

● この写真の内容を知っていますか?

□知っている　□知らないが興味がある

□知らない、興味がない

**110.コンバーター**

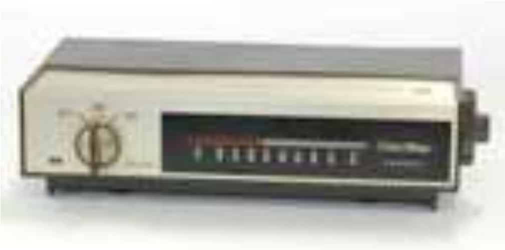

● この写真のお話しをしたいですか?

□好き　□感じない　□嫌い

● この写真の内容を知っていますか?

□知っている　□知らないが興味がある

□知らない、興味がない

**111.掃除機**

ほうきとちりとりを”一掃”

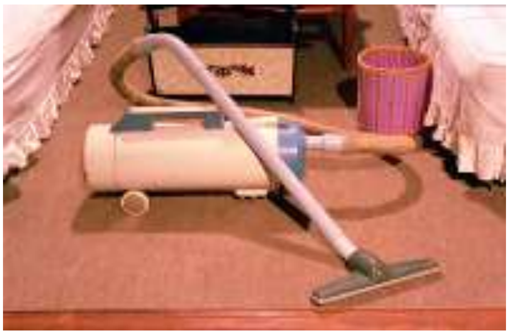

● この写真のお話しをしたいですか?

□好き　□感じない　□嫌い

● この写真の内容を知っていますか?

□知っている　□知らないが興味がある

□知らない、興味がない

**112.テレビ**

茶の間の視線くぎづけ

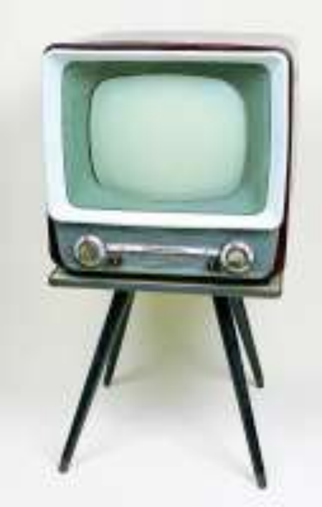

● この写真のお話しをしたいですか?

□好き　□感じない　□嫌い

● この写真の内容を知っていますか?

□知っている　□知らないが興味がある

□知らない、興味がない

**113.チャンネル**

丸くて、『回す』ものだった

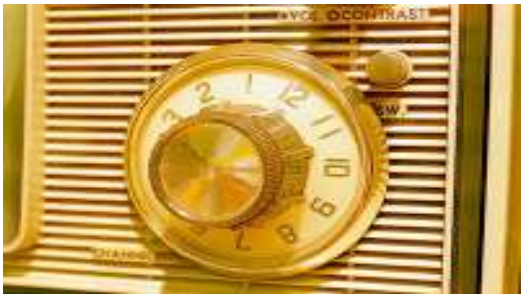

● この写真のお話しをしたいですか?

□好き　□感じない　□嫌い

● この写真の内容を知っていますか?

□知っている　□知らないが興味がある

□知らない、興味がない

**114.電気炊飯器**

時代とともに変化　振り返ろう自分史

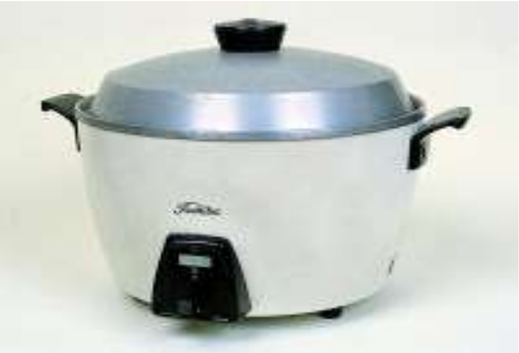

● この写真のお話しをしたいですか?
☐好き　☐感じない　☐嫌い
● この写真の内容を知っていますか?
☐知っている　☐知らないが興味がある
☐知らない、興味がない

**115.電気洗濯機**
手で回すローラー式絞り器

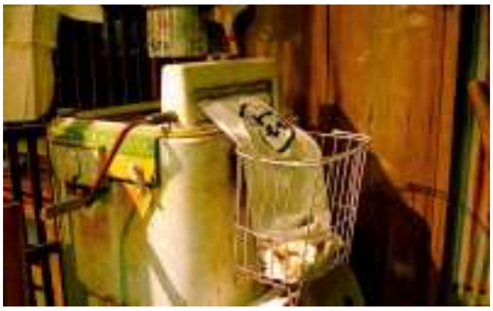

● この写真のお話しをしたいですか?
☐好き　☐感じない　☐嫌い
● この写真の内容を知っていますか?
☐知っている　☐知らないが興味がある
☐知らない、興味がない

**116.ゆで卵器**
用途と形が一致　隠れた人気商品

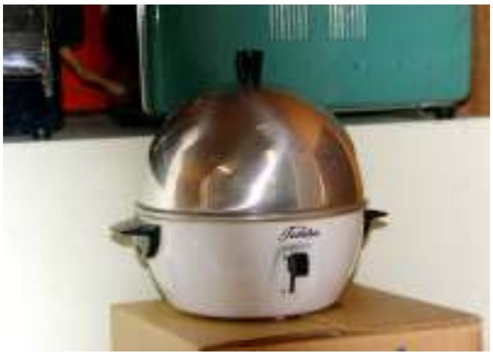

● この写真のお話しをしたいですか?
☐好き　☐感じない　☐嫌い
● この写真の内容を知っていますか?
☐知っている　☐知らないが興味がある
☐知らない、興味がない

**117.キャラメル**
100 年の歴史持つ昔のおやつの代名詞

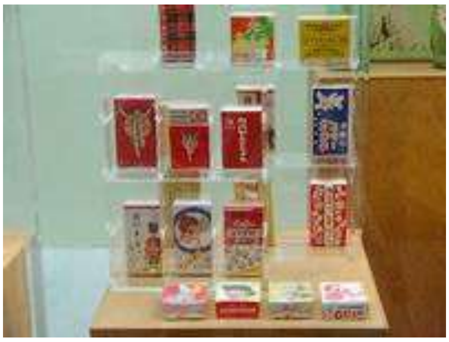

● この写真のお話しをしたいですか?
☐好き　☐感じない　☐嫌い
● この写真の内容を知っていますか?
☐知っている　☐知らないが興味がある
☐知らない、興味がない

**118.ラムネと栓抜き**

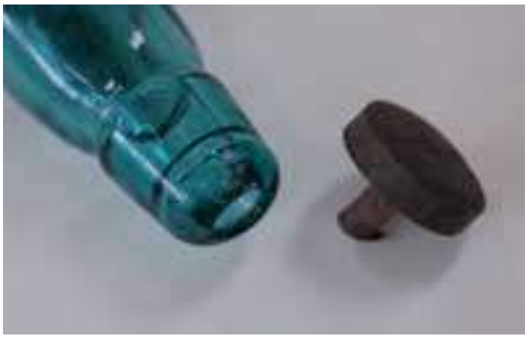

● この写真のお話しをしたいですか?
☐好き　☐感じない　☐嫌い
● この写真の内容を知っていますか?
☐知っている　☐知らないが興味がある
☐知らない、興味がない

**119.牛乳瓶のふた**
メンコやこま代わりに子供の頃の思い出

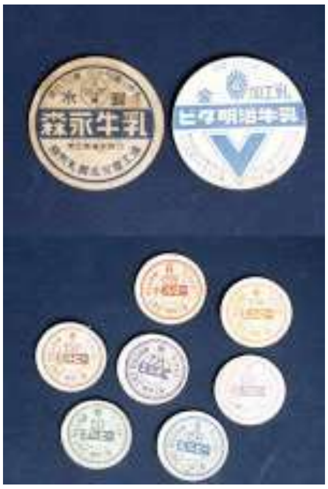


- この写真のお話しをしたいですか?

☐好き　　☐感じない　　☐嫌い

- この写真の内容を知っていますか?

☐知っている　☐知らないが興味がある

☐知らない、興味がない

**120.ドロップ**

今も子どもたちを魅了

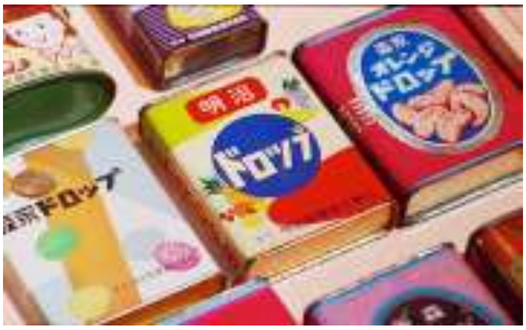


- この写真のお話しをしたいですか?

☐好き　　☐感じない　　☐嫌い

- この写真の内容を知っていますか?

☐知っている　☐知らないが興味がある

☐知らない、興味がない

**121.粉末ジュース**

“家庭の味”楽しんだ懐かしい即席飲料

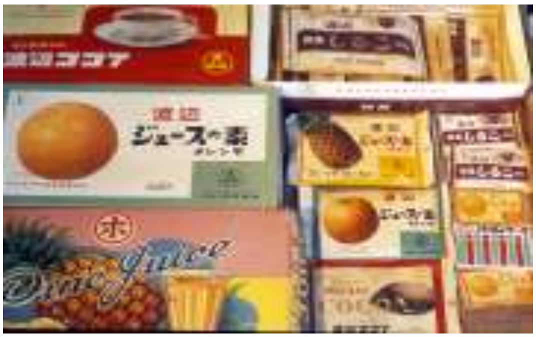

- この写真のお話しをしたいですか?

☐好き　　☐感じない　　☐嫌い

- この写真の内容を知っていますか?

☐知っている　☐知らないが興味がある

☐知らない、興味がない

**122.即席カレー**

粉末からレトルトへ作り方がより簡便に

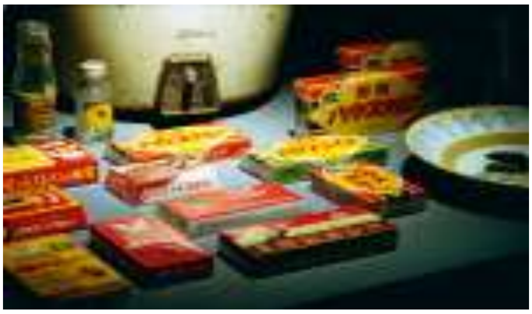

- この写真のお話しをしたいですか?

☐好き　　☐感じない　　☐嫌い

- この写真の内容を知っていますか?

☐知っている　☐知らないが興味がある

☐知らない、興味がない

**123.冷菓**

キャンデー主流から乳製品のアイスへ

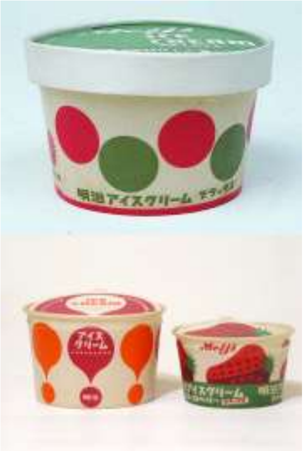

- この写真のお話しをしたいですか?

☐好き　　☐感じない　　☐嫌い

- この写真の内容を知っていますか?

☐知っている　☐知らないが興味がある

☐知らない、興味がない

**124.魔法瓶**

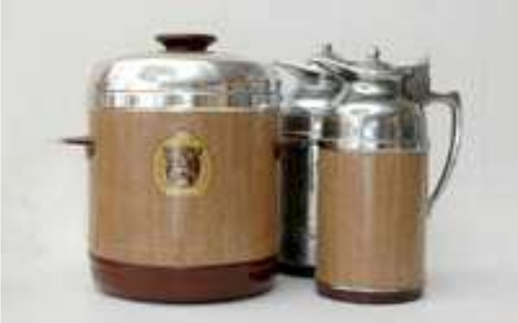

● この写真のお話しをしたいですか?
☐好き　☐感じない　☐嫌い
● この写真の内容を知っていますか?
☐知っている　☐知らないが興味がある
☐知らない、興味がない

**125.牛乳受け**

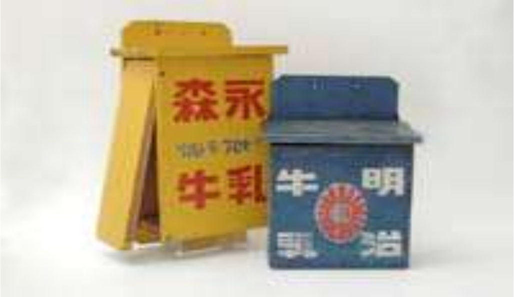


● この写真のお話しをしたいですか?
☐好き　☐感じない　☐嫌い
● この写真の内容を知っていますか?
☐知っている　☐知らないが興味がある
☐知らない、興味がない

**126.徳利**

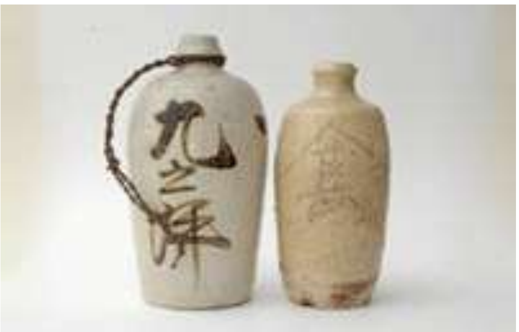

● この写真のお話しをしたいですか?
☐好き　☐感じない　☐嫌い
● この写真の内容を知っていますか?
☐知っている　☐知らないが興味がある
☐知らない、興味がない

**127.手ぬぐい**

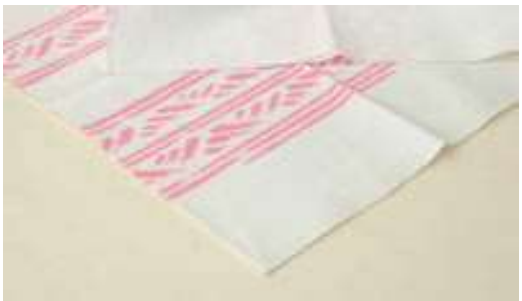

● この写真のお話しをしたいですか?
☐好き　☐感じない　☐嫌い
● この写真の内容を知っていますか?
☐知っている　☐知らないが興味がある
☐知らない、興味がない

**128.団扇・扇子**

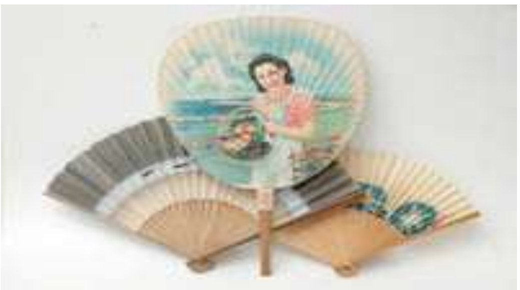

● この写真のお話しをしたいですか?
☐好き　☐感じない　☐嫌い
● この写真の内容を知っていますか?
☐知っている　☐知らないが興味がある
☐知らない、興味がない

**129.ぜんまい仕掛け**

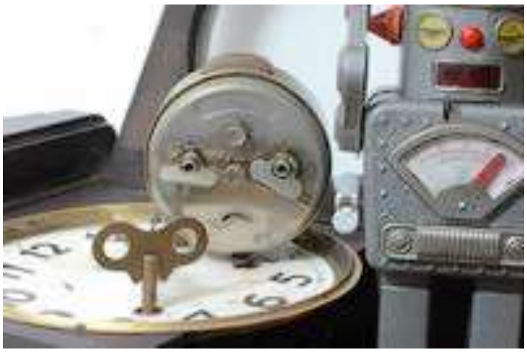

● この写真のお話しをしたいですか?
☐好き　☐感じない　☐嫌い
● この写真の内容を知っていますか?
☐知っている　☐知らないが興味がある
☐知らない、興味がない

**130.風呂敷**

キャンデー主流から乳製品のアイスへ

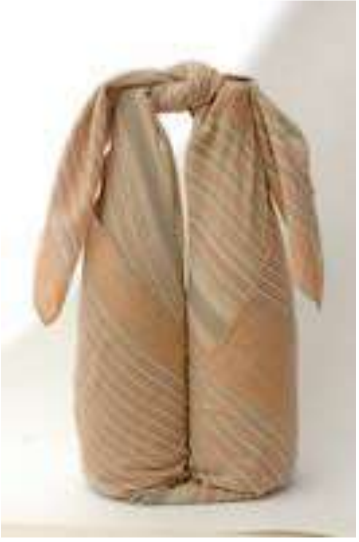

● この写真のお話しをしたいですか?

☐好き ☐感じない ☐嫌い

● この写真の内容を知っていますか?

☐知っている ☐知らないが興味がある

☐知らない、興味がない

## 景観

**1.札幌**

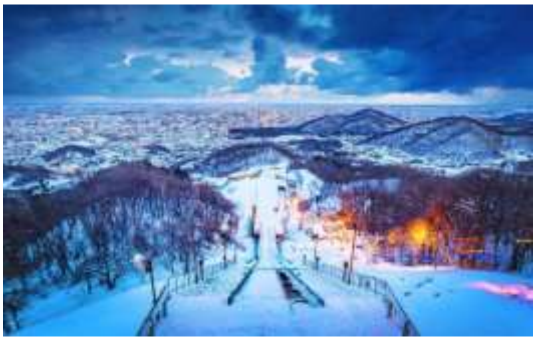

● この写真のお話しをしたいですか?

☐好き ☐感じない ☐嫌い

● この写真の内容を知っていますか?

☐知っている ☐知らないが興味がある

☐知らない、興味がない

**2.东京都**

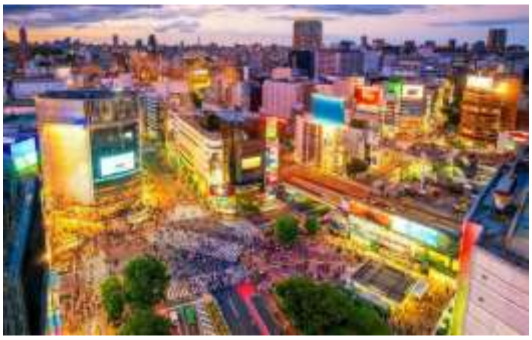

● この写真のお話しをしたいですか?

☐好き ☐感じない ☐嫌い

● この写真の内容を知っていますか?

☐知っている ☐知らないが興味がある

☐知らない、興味がない

**3.横浜**

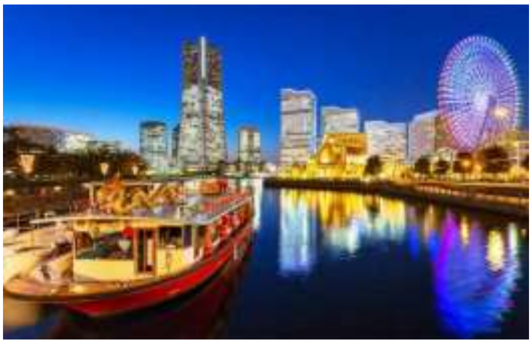

● この写真のお話しをしたいですか?

☐好き ☐感じない ☐嫌い

● この写真の内容を知っていますか?

☐知っている ☐知らないが興味がある

☐知らない、興味がない

**4.名古屋**

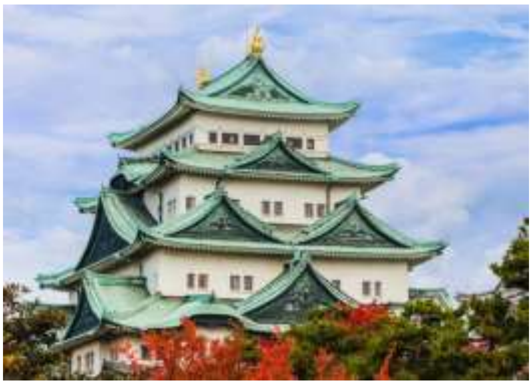

● この写真のお話しをしたいですか?

☐好き　　☐感じない　　☐嫌い

● この写真の内容を知っていますか?

☐知っている　☐知らないが興味がある

☐知らない、興味がない

**5.京都**

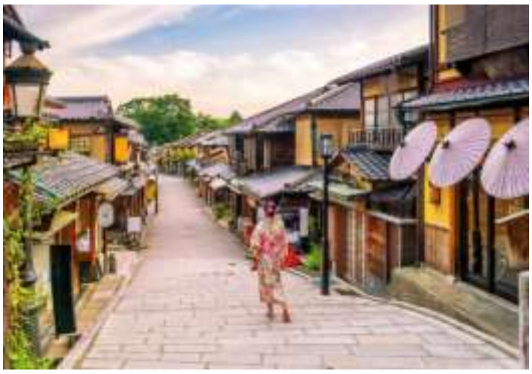

● この写真のお話しをしたいですか?

☐好き　　☐感じない　　☐嫌い

● この写真の内容を知っていますか?

☐知っている　☐知らないが興味がある

☐知らない、興味がない

**6.奈良**

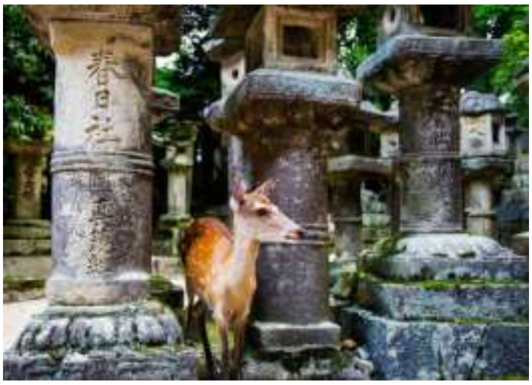

● この写真のお話しをしたいですか?

☐好き　　☐感じない　　☐嫌い

● この写真の内容を知っていますか?

☐知っている　☐知らないが興味がある

☐知らない、興味がない

**7.大阪**

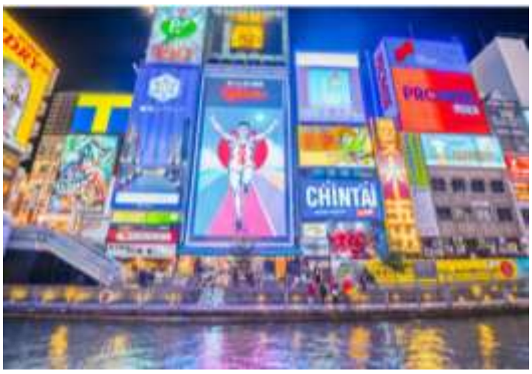


● この写真のお話しをしたいですか?

☐好き　　☐感じない　　☐嫌い

● この写真の内容を知っていますか?

☐知っている　☐知らないが興味がある

☐知らない、興味がない

**8.神戸**

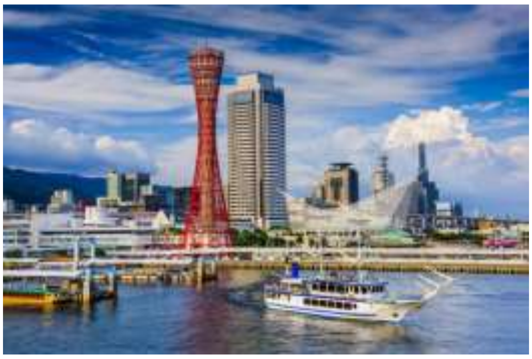

● この写真のお話しをしたいですか?

☐好き　　☐感じない　　☐嫌い

● この写真の内容を知っていますか?

☐知っている　☐知らないが興味がある

☐知らない、興味がない

**9.広島**

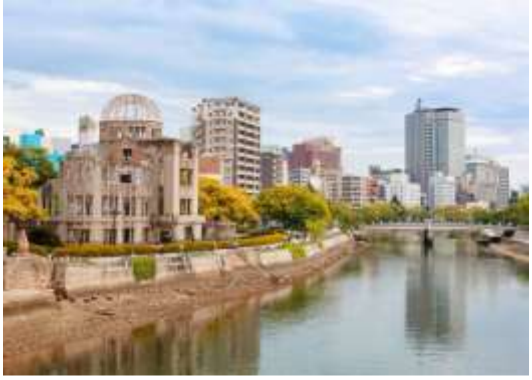

● この写真のお話しをしたいですか?
☐好き ☐感じない ☐嫌い
● この写真の内容を知っていますか?
☐知っている ☐知らないが興味がある
☐知らない、興味がない

**10.沖縄**

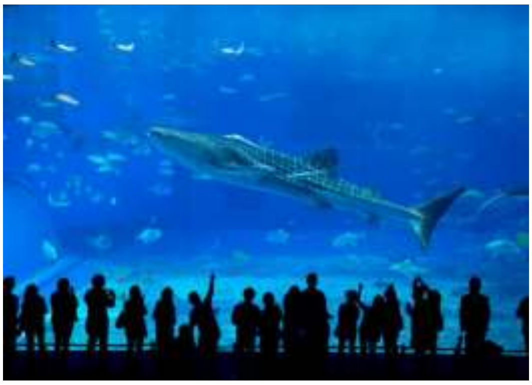

● この写真のお話しをしたいですか?
☐好き ☐感じない ☐嫌い
● この写真の内容を知っていますか?
☐知っている ☐知らないが興味がある
☐知らない、興味がない

**11.奈良 東大寺**

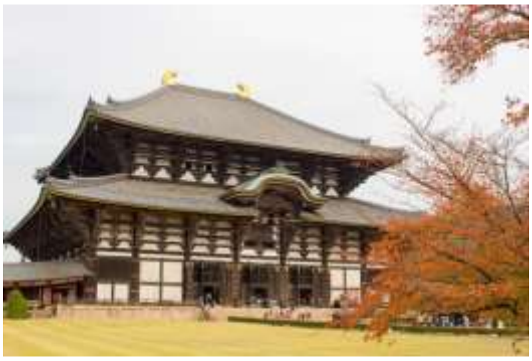

● この写真のお話しをしたいですか?
☐好き ☐感じない ☐嫌い
● この写真の内容を知っていますか?
☐知っている ☐知らないが興味がある
☐知らない、興味がない

**12.兵庫縣 姫路城**

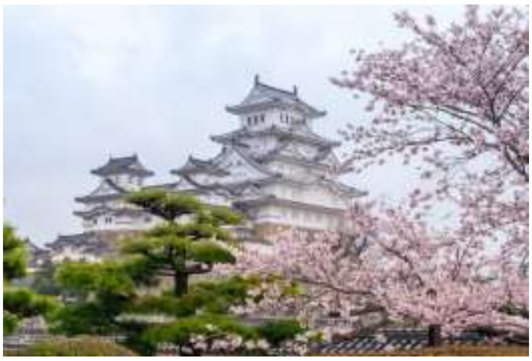

● この写真のお話しをしたいですか?
☐好き ☐感じない ☐嫌い
● この写真の内容を知っていますか?
☐知っている ☐知らないが興味がある
☐知らない、興味がない

**13.山梨縣 河口湖**

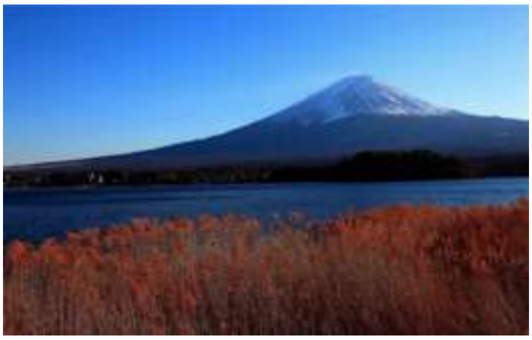

● この写真のお話しをしたいですか?
☐好き ☐感じない ☐嫌い
● この写真の内容を知っていますか?
☐知っている ☐知らないが興味がある
☐知らない、興味がない

**14.三重縣桑名市 なばなの里**

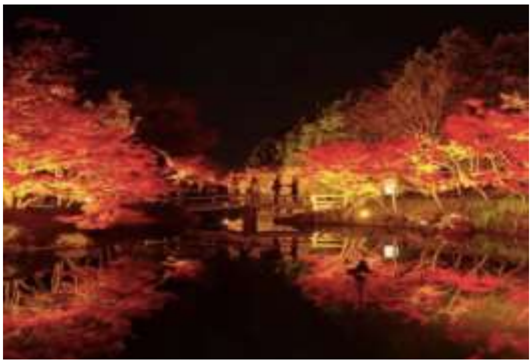

● この写真のお話しをしたいですか?
□好き　□感じない　□嫌い
● この写真の内容を知っていますか?
□知っている　□知らないが興味がある
□知らない、興味がない

**15.千葉縣成田市 新勝寺**

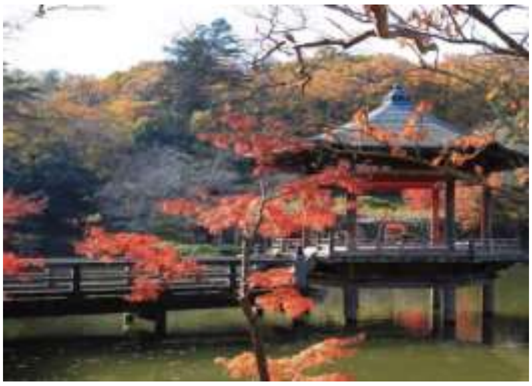

● この写真のお話しをしたいですか?
□好き　□感じない　□嫌い
● この写真の内容を知っていますか?
□知っている　□知らないが興味がある
□知らない、興味がない

**16.神奈川縣 鎌倉市 長谷寺**

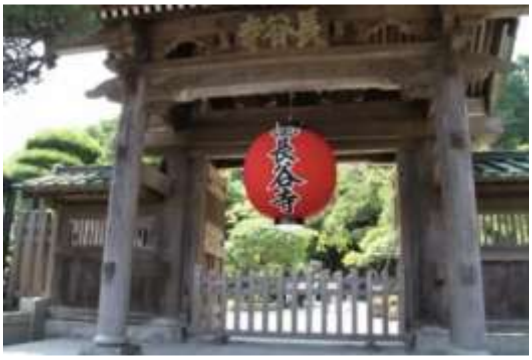

● この写真のお話しをしたいですか?
□好き　□感じない　□嫌い
● この写真の内容を知っていますか?
□知っている　□知らないが興味がある
□知らない、興味がない

**17.廣島 厳島神社**

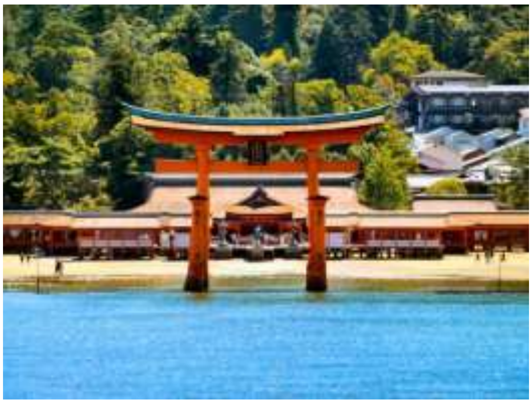

● この写真のお話しをしたいですか?
□好き　□感じない　□嫌い
● この写真の内容を知っていますか?
□知っている　□知らないが興味がある
□知らない、興味がない

**18.廣島 廣島和平紀念資料館**

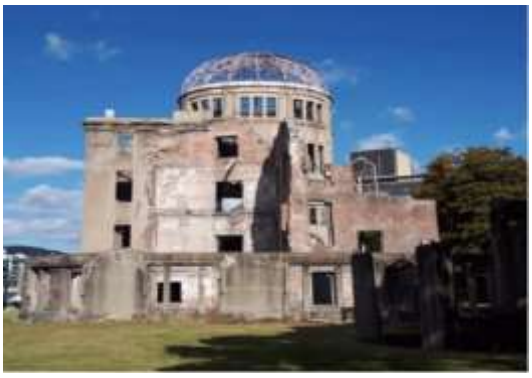

● この写真のお話しをしたいですか?
□好き　□感じない　□嫌い
● この写真の内容を知っていますか?
□知っている　□知らないが興味がある
□知らない、興味がない

**19.神奈川 箱根雕刻の森美術館**

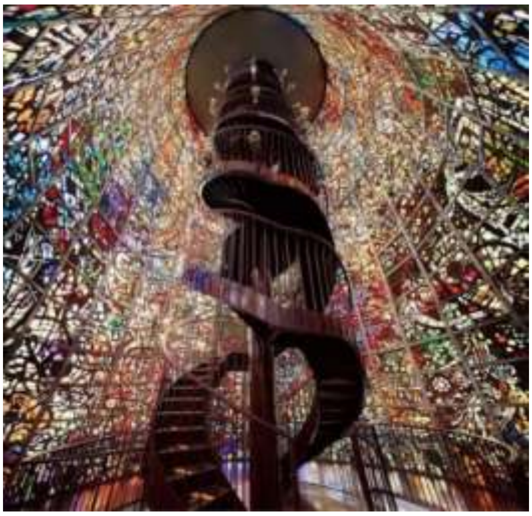

● この写真のお話しをしたいですか?
□好き　□感じない　□嫌い
● この写真の内容を知っていますか?
□知っている　□知らないが興味がある
□知らない、興味がない

**20.兵庫縣 姫路城**

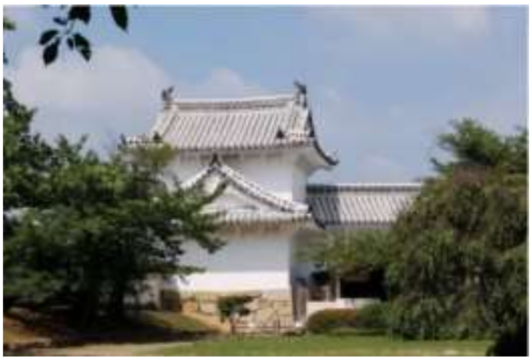

● この写真のお話しをしたいですか?
□好き　□感じない　□嫌い
● この写真の内容を知っていますか?
□知っている　□知らないが興味がある
□知らない、興味がない

**21.東京晴空塔**

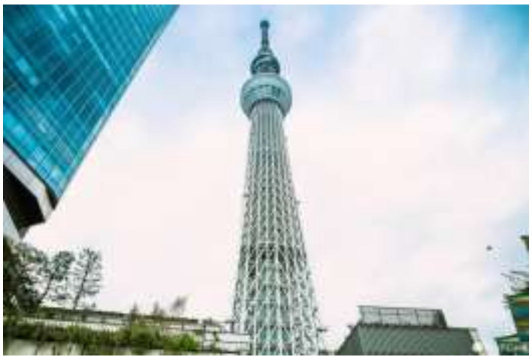

● この写真のお話しをしたいですか?
□好き　□感じない　□嫌い
● この写真の内容を知っていますか?
□知っている　□知らないが興味がある
□知らない、興味がない

**22.北海道 富良野**

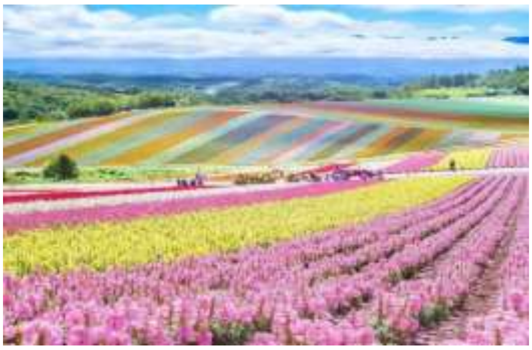

● この写真のお話しをしたいですか?
□好き　□感じない　□嫌い
● この写真の内容を知っていますか?
□知っている　□知らないが興味がある
□知らない、興味がない

**23.北海道 札幌　小樽運河**

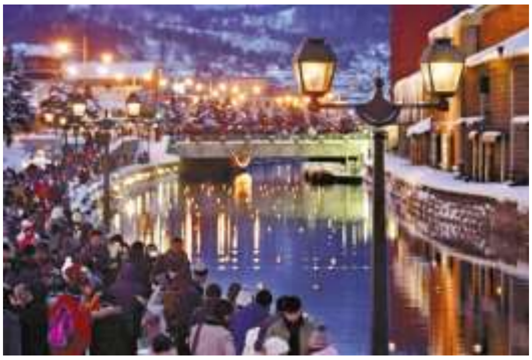

● この写真のお話しをしたいですか?
□好き　□感じない　□嫌い
● この写真の内容を知っていますか?
□知っている　□知らないが興味がある
□知らない、興味がない

**24.京都三十三間堂**

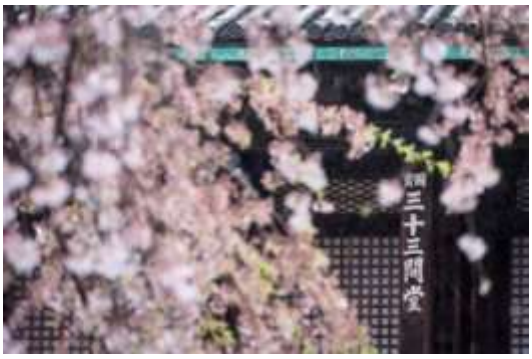


● この写真のお話しをしたいですか?
☐好き ☐感じない ☐嫌い
● この写真の内容を知っていますか?
☐知っている ☐知らないが興味がある
☐知らない、興味がない

**25.京都駅ビル**

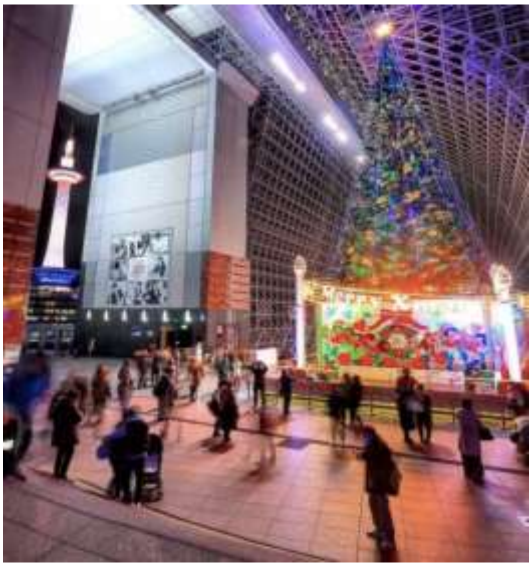

● この写真のお話しをしたいですか?
☐好き ☐感じない ☐嫌い
● この写真の内容を知っていますか?
☐知っている ☐知らないが興味がある
☐知らない、興味がない

**26.京都 金閣寺**

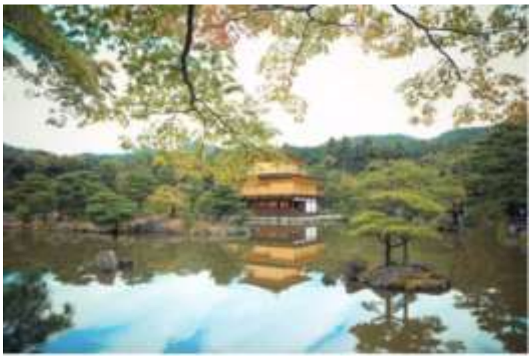

● この写真のお話しをしたいですか?
☐好き ☐感じない ☐嫌い
● この写真の内容を知っていますか?
☐知っている ☐知らないが興味がある
☐知らない、興味がない

**27.京都嵐山電車**

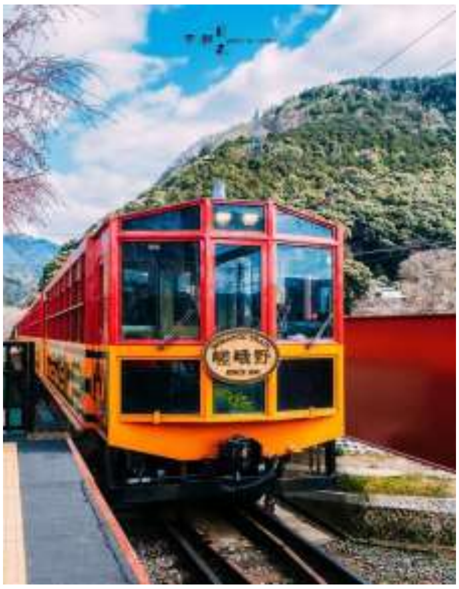


● この写真のお話しをしたいですか?
☐好き ☐感じない ☐嫌い
● この写真の内容を知っていますか?
☐知っている ☐知らないが興味がある
☐知らない、興味がない

**28.京都 伏見稲荷大社**

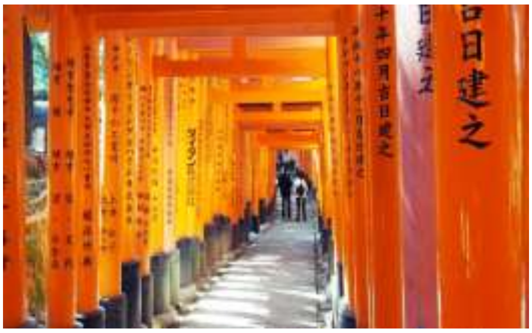

● この写真のお話しをしたいですか?
☐好き ☐感じない ☐嫌い
● この写真の内容を知っていますか?
☐知っている ☐知らないが興味がある
☐知らない、興味がない

**29.京都 渡月橋**

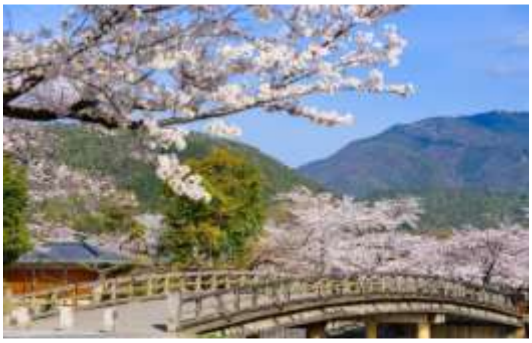

● この写真のお話しをしたいですか?
☐好き ☐感じない ☐嫌い
● この写真の内容を知っていますか?
☐知っている ☐知らないが興味がある
☐知らない、興味がない

**30.京都 清水寺**

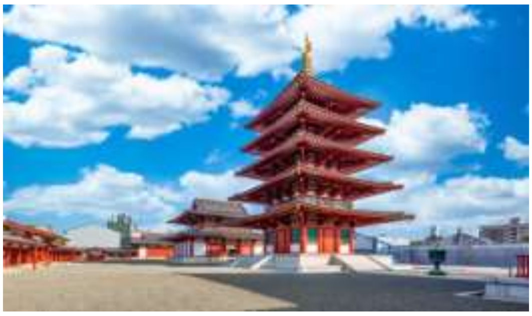

● この写真のお話しをしたいですか?
☐好き ☐感じない ☐嫌い
● この写真の内容を知っていますか?
☐知っている ☐知らないが興味がある
☐知らない、興味がない

**31.大阪城公園**

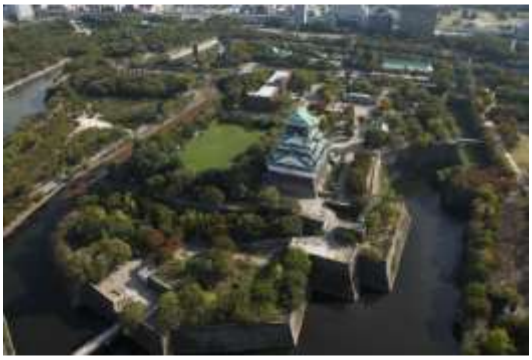

● この写真のお話しをしたいですか?
☐好き ☐感じない ☐嫌い
● この写真の内容を知っていますか?
☐知っている ☐知らないが興味がある
☐知らない、興味がない

**32.大阪 阿倍野展望台 HARUKAS**

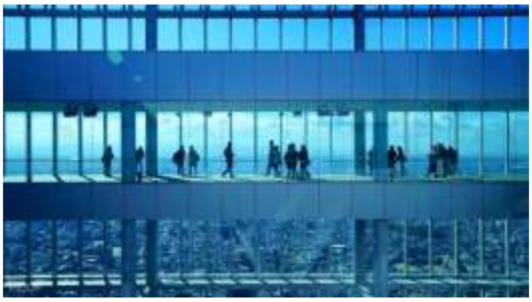

● この写真のお話しをしたいですか?
☐好き ☐感じない ☐嫌い
● この写真の内容を知っていますか?
☐知っている ☐知らないが興味がある
☐知らない、興味がない

**33.大阪 黑門市場**

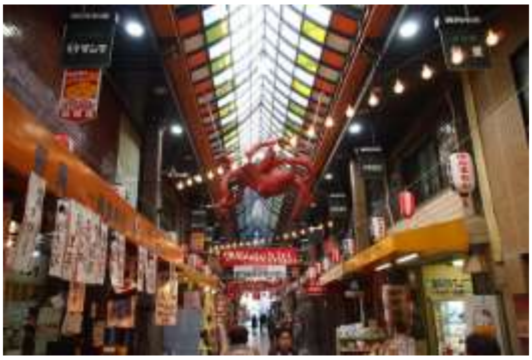

● この写真のお話しをしたいですか?
☐好き ☐感じない ☐嫌い
● この写真の内容を知っていますか?
☐知っている ☐知らないが興味がある
☐知らない、興味がない

**34.大阪 四大天王寺**

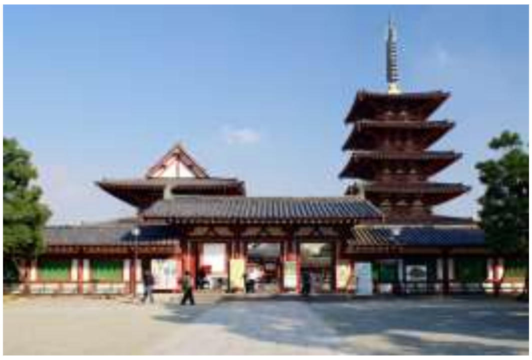

● この写真のお話しをしたいですか?

☐好き　☐感じない　☐嫌い

● この写真の内容を知っていますか?

☐知っている　☐知らないが興味がある

☐知らない、興味がない

**35.大阪 大阪生活今昔館**

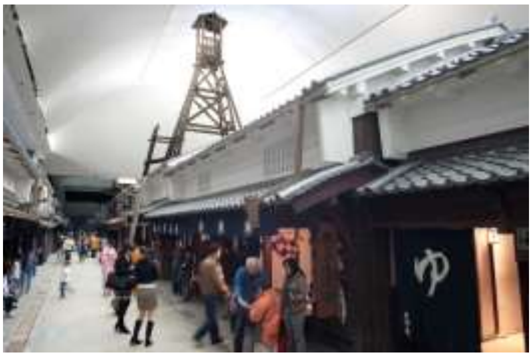


● この写真のお話しをしたいですか?

☐好き　☐感じない　☐嫌い

● この写真の内容を知っていますか?

☐知っている　☐知らないが興味がある

☐知らない、興味がない

# 食べ物

## 1.だし

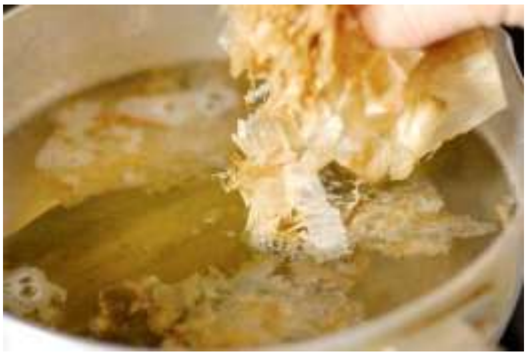

- この写真のお話しをしたいですか?

☐好き　☐感じない　☐嫌い

- この写真の内容を知っていますか?

☐知っている　☐知らないが興味がある
☐知らない、興味がない

## 2.唐菓子

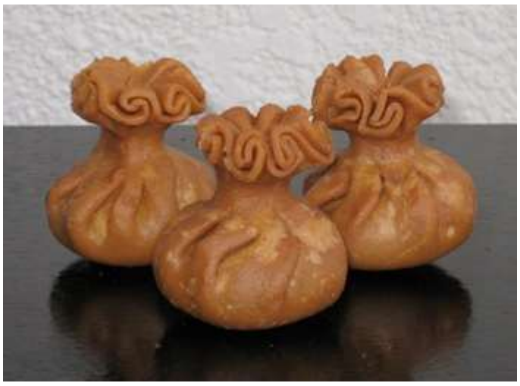

- この写真のお話しをしたいですか?

☐好き　☐感じない　☐嫌い

- この写真の内容を知っていますか?

☐知っている　☐知らないが興味がある
☐知らない、興味がない

## 3.がんもどき

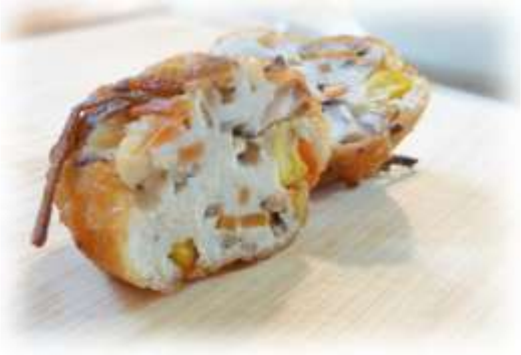

- この写真のお話しをしたいですか?

☐好き　☐感じない　☐嫌い

- この写真の内容を知っていますか?

☐知っている　☐知らないが興味がある
☐知らない、興味がない

## 4.豆腐的田乐

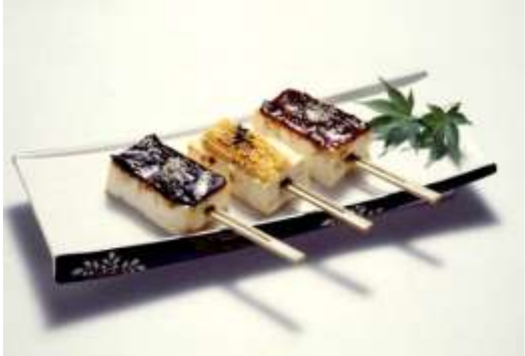

- この写真のお話しをしたいですか?

☐好き　☐感じない　☐嫌い

- この写真の内容を知っていますか?

☐知っている　☐知らないが興味がある
☐知らない、興味がない

## 5.本膳料理

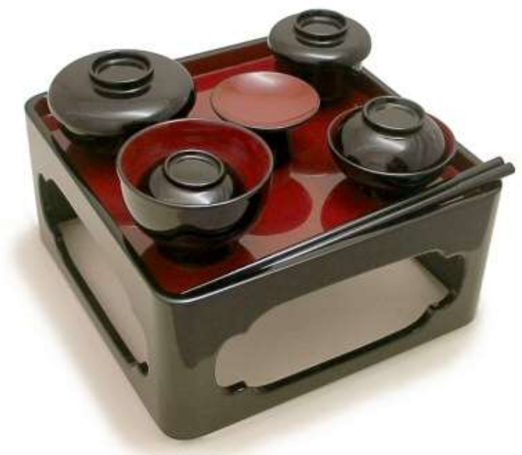

- この写真のお話しをしたいですか?

☐好き　☐感じない　☐嫌い

- この写真の内容を知っていますか?

☐知っている　☐知らないが興味がある
☐知らない、興味がない

## 6.会席料理

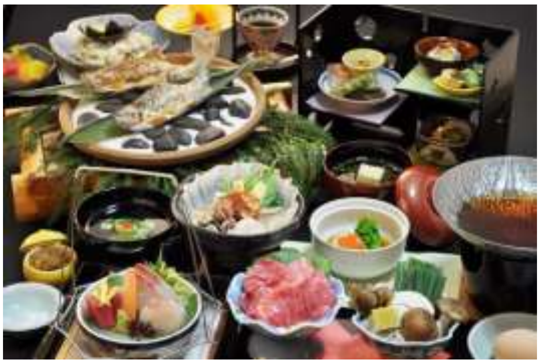

● この写真のお話しをしたいですか?
☐好き　☐感じない　☐嫌い
● この写真の内容を知っていますか?
☐知っている　☐知らないが興味がある
☐知らない、興味がない

**7.京野菜**

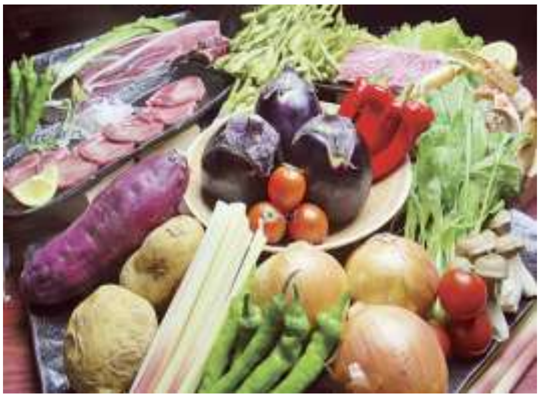

● この写真のお話しをしたいですか?
☐好き　☐感じない　☐嫌い
● この写真の内容を知っていますか?
☐知っている　☐知らないが興味がある
☐知らない、興味がない

**8.すきやき**

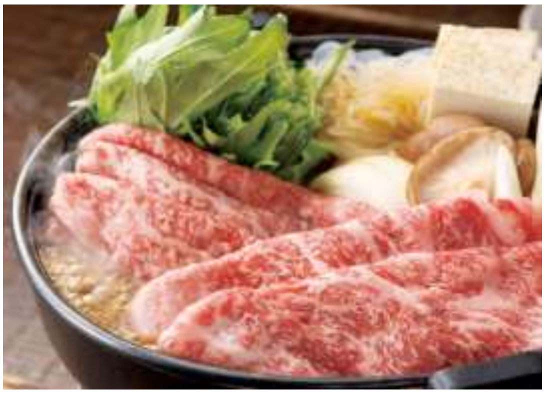

● この写真のお話しをしたいですか?
☐好き　☐感じない　☐嫌い
● この写真の内容を知っていますか?
☐知っている　☐知らないが興味がある
☐知らない、興味がない

**9.肉じゃが**

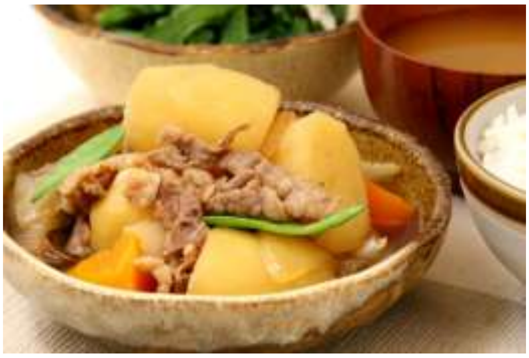

● この写真のお話しをしたいですか?
☐好き　☐感じない　☐嫌い
● この写真の内容を知っていますか?
☐知っている　☐知らないが興味がある
☐知らない、興味がない

**10.カツ**

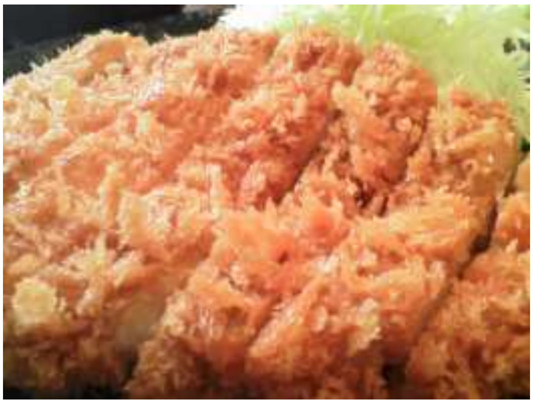

● この写真のお話しをしたいですか?
☐好き　☐感じない　☐嫌い
● この写真の内容を知っていますか?
☐知っている　☐知らないが興味がある
☐知らない、興味がない

**11. フライ**

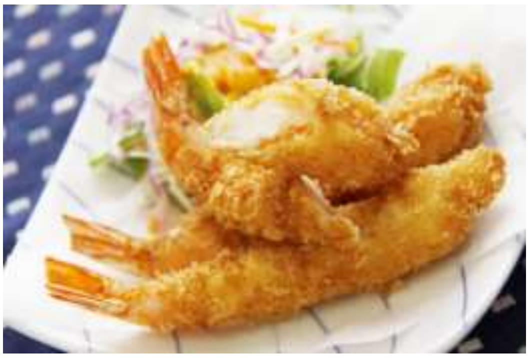

● この写真のお話しをしたいですか?
□好き　　□感じない　　□嫌い
● この写真の内容を知っていますか?
□知っている　□知らないが興味がある
□知らない、興味がない

**12.らーめん**

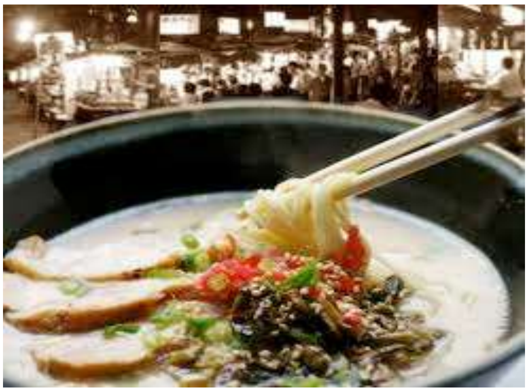

● この写真のお話しをしたいですか?
□好き　　□感じない　　□嫌い
● この写真の内容を知っていますか?
□知っている　□知らないが興味がある
□知らない、興味がない

**13.御田（おでん）**

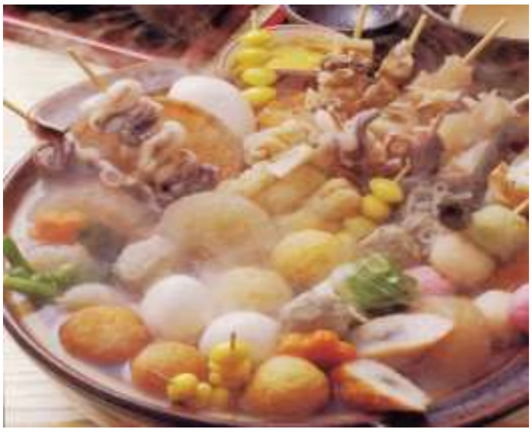

● この写真のお話しをしたいですか?
□好き　　□感じない　　□嫌い
● この写真の内容を知っていますか?
□知っている　□知らないが興味がある
□知らない、興味がない

**14.佃煮（つくだに）**

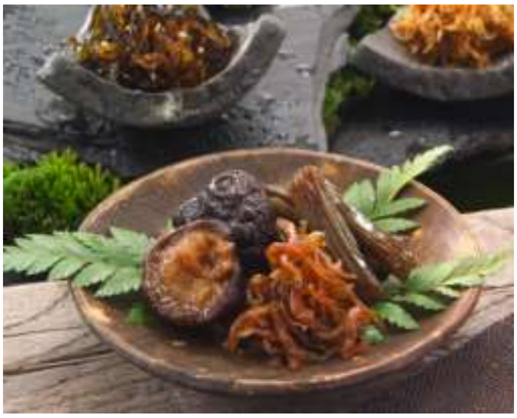

● この写真のお話しをしたいですか?
□好き　　□感じない　　□嫌い
● この写真の内容を知っていますか?
□知っている　□知らないが興味がある
□知らない、興味がない

**15.时雨煮（しぐれに）**

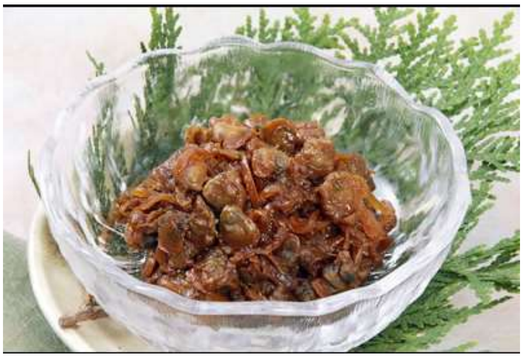

● この写真のお話しをしたいですか?
□好き　　□感じない　　□嫌い
● この写真の内容を知っていますか?
□知っている　□知らないが興味がある
□知らない、興味がない

**16.蒲烧鳗鱼**

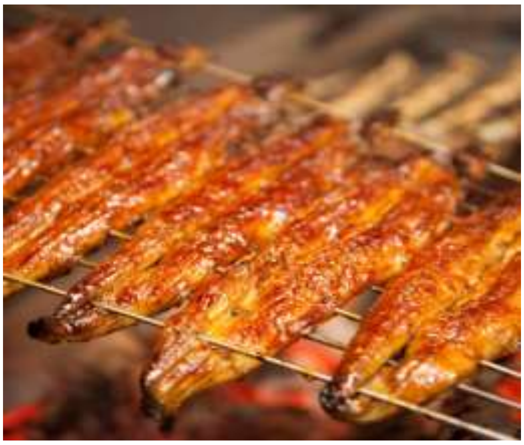

● この写真のお話しをしたいですか?
☐好き ☐感じない ☐嫌い
● この写真の内容を知っていますか?
☐知っている ☐知らないが興味がある
☐知らない、興味がない

**17.甘露煮（かんろに）**

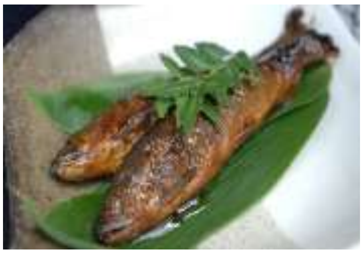

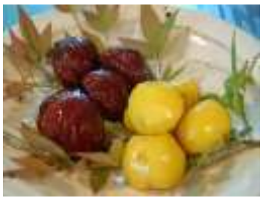

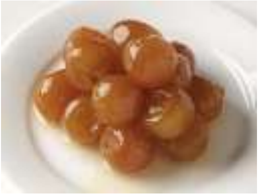

● この写真のお話しをしたいですか?
☐好き ☐感じない ☐嫌い
● この写真の内容を知っていますか?
☐知っている ☐知らないが興味がある
☐知らない、興味がない

**18.にごり酒**

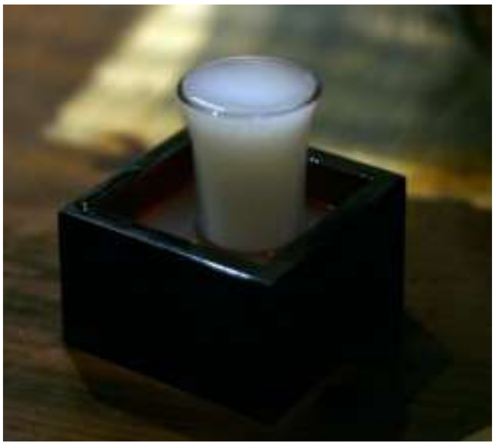

● この写真のお話しをしたいですか?
☐好き ☐感じない ☐嫌い
● この写真の内容を知っていますか?
☐知っている ☐知らないが興味がある
☐知らない、興味がない

19.清酒

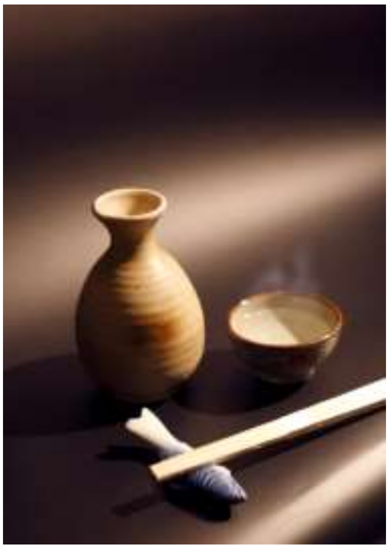

● この写真のお話しをしたいですか?
☐好き ☐感じない ☐嫌い
● この写真の内容を知っていますか?
☐知っている ☐知らないが興味がある
☐知らない、興味がない

20.樽酒（たるざけ）

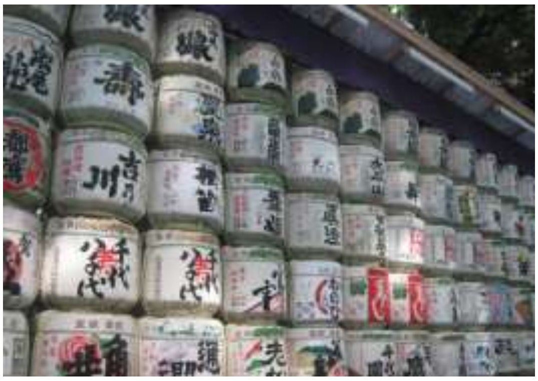

● この写真のお話しをしたいですか?

☐好き ☐感じない ☐嫌い

● この写真の内容を知っていますか?

☐知っている ☐知らないが興味がある

☐知らない、興味がない

**21.ひやおろし**

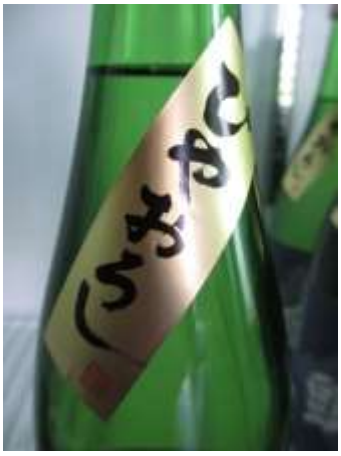


● この写真のお話しをしたいですか?

☐好き ☐感じない ☐嫌い

● この写真の内容を知っていますか?

☐知っている ☐知らないが興味がある

☐知らない、興味がない

**22.甘酒（あまざけ）**

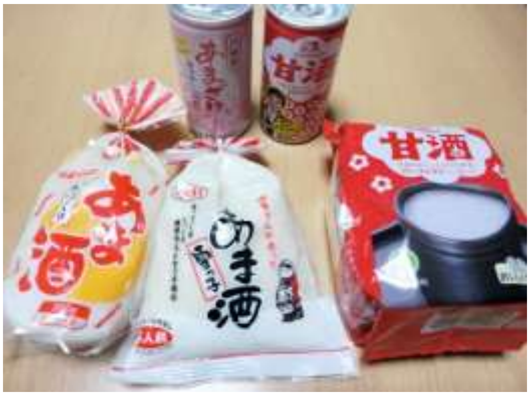


● この写真のお話しをしたいですか?

☐好き ☐感じない ☐嫌い

● この写真の内容を知っていますか?

☐知っている ☐知らないが興味がある

☐知らない、興味がない

**23. 利き酒（ききざけ）**

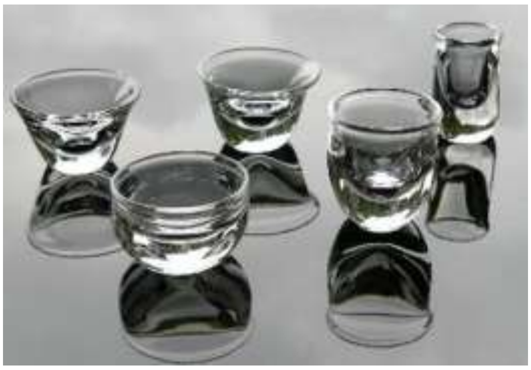

● この写真のお話しをしたいですか?

☐好き ☐感じない ☐嫌い

● この写真の内容を知っていますか?

☐知っている ☐知らないが興味がある

☐知らない、興味がない

24.

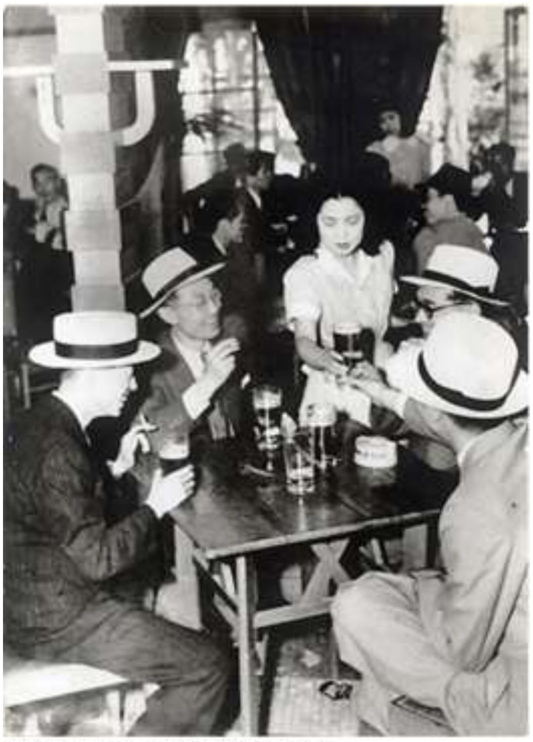

1949年、ビアホールの営業が再開された。

● この写真のお話しをしたいですか?

☐好き ☐感じない ☐嫌い

- ● この写真の内容を知っていますか?

☐知っている　☐知らないが興味がある
☐知らない、興味がない

25.

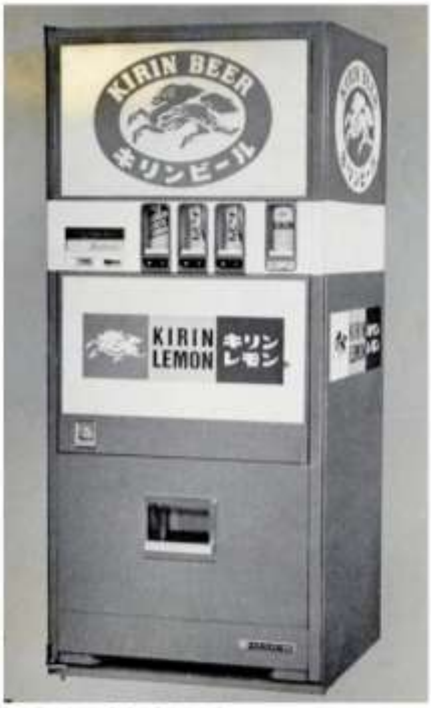

1973年のキリン製品の自動販売機

- ● この写真のお話しをしたいですか?

☐好き　☐感じない　☐嫌い

- ● この写真の内容を知っていますか?

☐知っている　☐知らないが興味がある
☐知らない、興味がない

26.

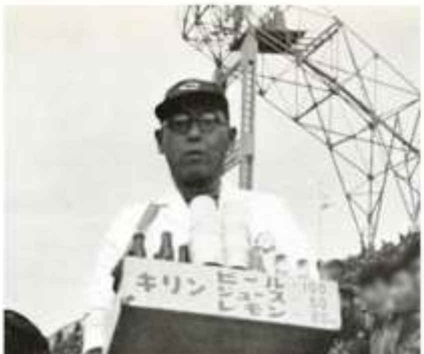

広島市民球場でのビール販売の様子

- ● この写真のお話しをしたいですか?

☐好き　☐感じない　☐嫌い

- ● この写真の内容を知っていますか?

☐知っている　☐知らないが興味がある
☐知らない、興味がない

27.

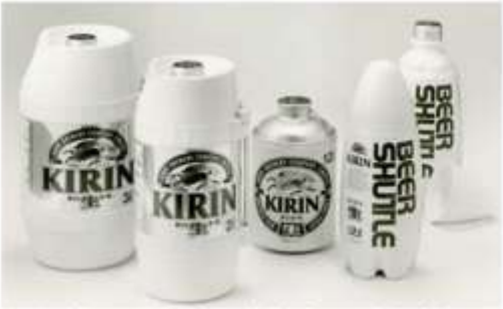

容器戦争と呼ばれていた頃の商品。「キリンのビヤ樽PET」「キリンのビヤ樽」「ビヤシャトル」

- ● この写真のお話しをしたいですか?

☐好き　☐感じない　☐嫌い

- ● この写真の内容を知っていますか?

☐知っている　☐知らないが興味がある
☐知らない、興味がない

# 行事

1.

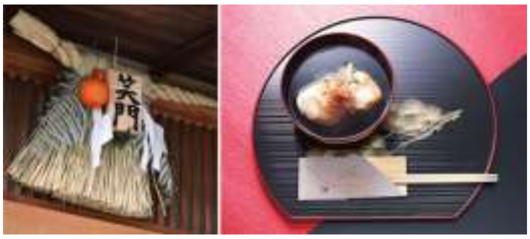

● この写真のお話しをしたいですか?

☐好き　☐感じない　☐嫌い

● この写真の内容を知っていますか?

☐知っている　☐知らないが興味がある

☐知らない、興味がない

2.

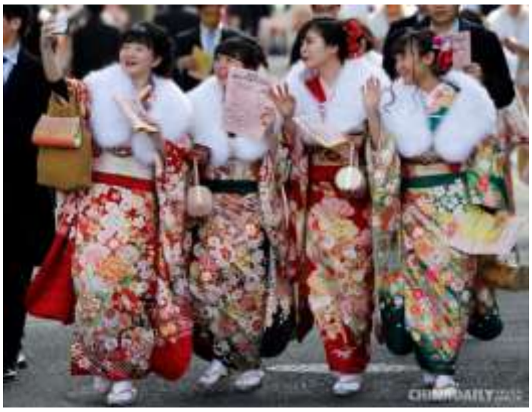

● この写真のお話しをしたいですか?

☐好き　☐感じない　☐嫌い

● この写真の内容を知っていますか?

☐知っている　☐知らないが興味がある

☐知らない、興味がない

3.

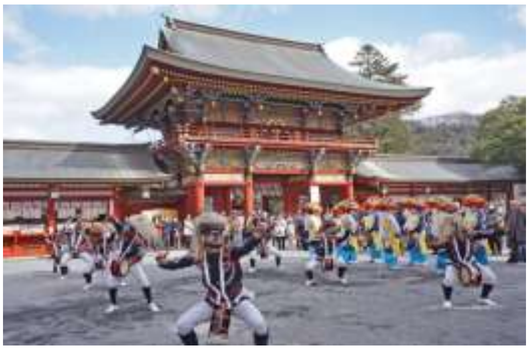

● この写真のお話しをしたいですか?

☐好き　☐感じない　☐嫌い

● この写真の内容を知っていますか?

☐知っている　☐知らないが興味がある

☐知らない、興味がない

4.

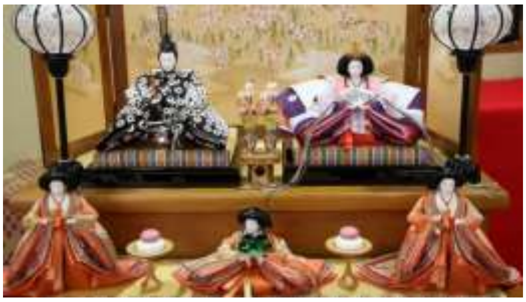

● この写真のお話しをしたいですか?

☐好き　☐感じない　☐嫌い

● この写真の内容を知っていますか?

☐知っている　☐知らないが興味がある

☐知らない、興味がない

5.

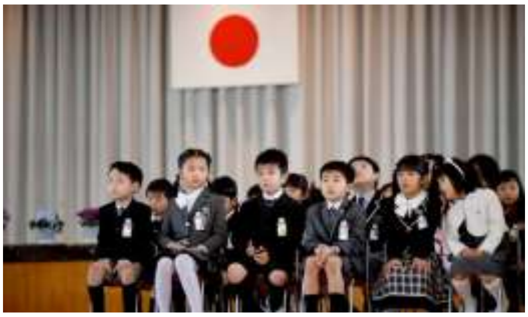

● この写真のお話しをしたいですか?

☐好き　☐感じない　☐嫌い

● この写真の内容を知っていますか?

☐知っている　☐知らないが興味がある

☐知らない、興味がない

6.

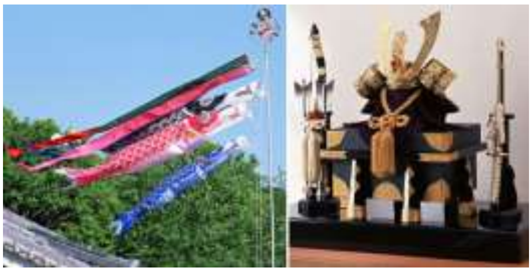

● この写真のお話しをしたいですか?

☐好き　☐感じない　☐嫌い

● この写真の内容を知っていますか?

☐知っている　☐知らないが興味がある

☐知らない、興味がない

7.

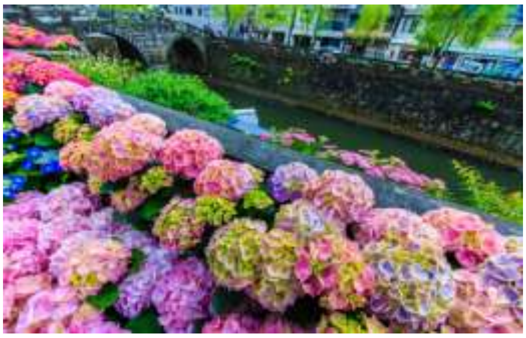

● この写真のお話しをしたいですか?

☐好き ☐感じない ☐嫌い

● この写真の内容を知っていますか?

☐知っている ☐知らないが興味がある

☐知らない、興味がない

8.

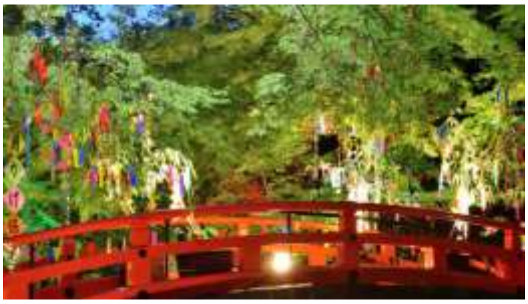

● この写真のお話しをしたいですか?

☐好き ☐感じない ☐嫌い

● この写真の内容を知っていますか?

☐知っている ☐知らないが興味がある

☐知らない、興味がない

9.

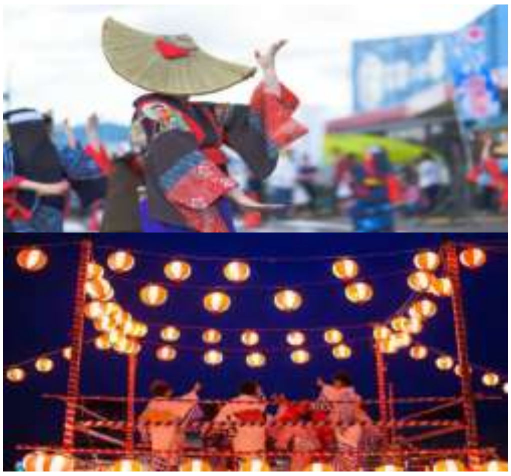

● この写真のお話しをしたいですか?

☐好き ☐感じない ☐嫌い

● この写真の内容を知っていますか?

☐知っている ☐知らないが興味がある

☐知らない、興味がない

10.

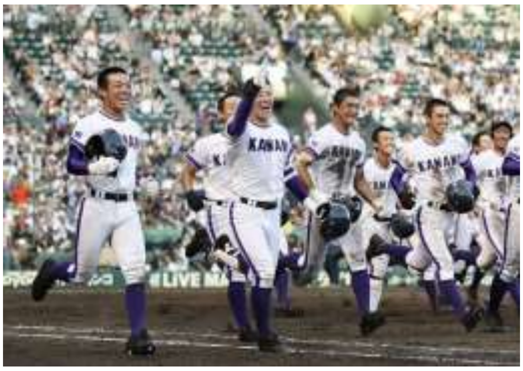

● この写真のお話しをしたいですか?

☐好き ☐感じない ☐嫌い

● この写真の内容を知っていますか?

☐知っている ☐知らないが興味がある

☐知らない、興味がない

11.

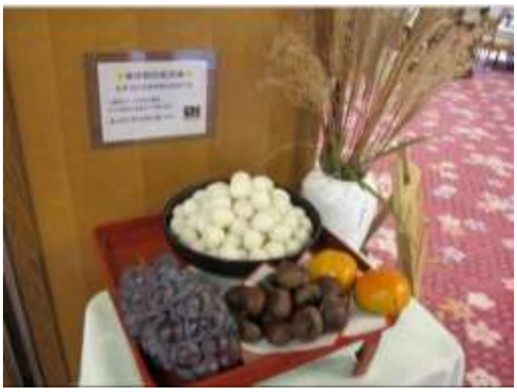

● この写真のお話しをしたいですか?

☐好き ☐感じない ☐嫌い

● この写真の内容を知っていますか?

☐知っている ☐知らないが興味がある

☐知らない、興味がない

12.

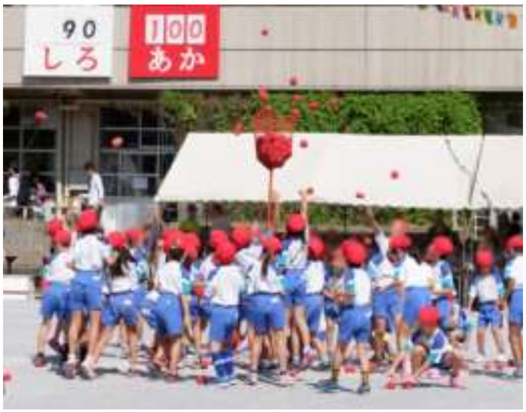

● この写真のお話しをしたいですか?
☐好き ☐感じない ☐嫌い
● この写真の内容を知っていますか?
☐知っている ☐知らないが興味がある
☐知らない、興味がない
13.
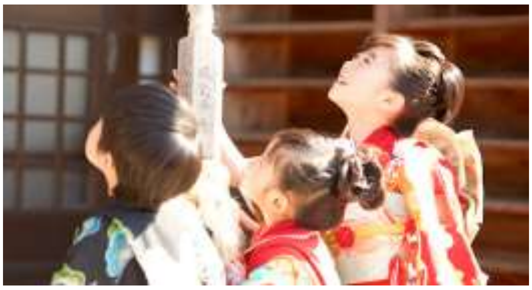
● この写真のお話しをしたいですか?
☐好き ☐感じない ☐嫌い
● この写真の内容を知っていますか?
☐知っている ☐知らないが興味がある
☐知らない、興味がない
14.
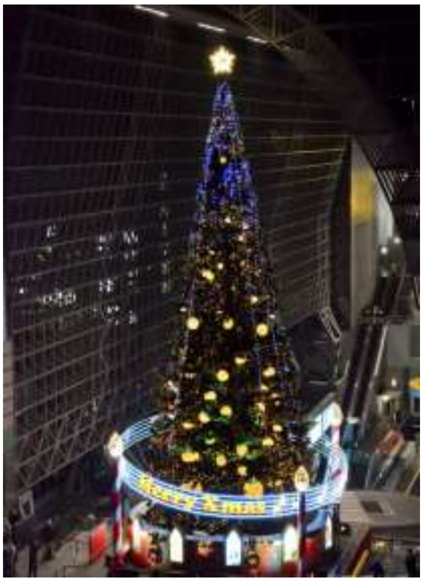
● この写真のお話しをしたいですか?
☐好き ☐感じない ☐嫌い
● この写真の内容を知っていますか?
☐知っている ☐知らないが興味がある
☐知らない、興味がない

# 写真に関する対話後の評価アンケート(Japanese version)

写真No.__________

- この写真に関する対話についてどう感じますか。下記の番号に○をつけてください。

### 対話は楽しいですか

| | 非常に | | やや | どちらともいえない | | | やや | | 非常に | |
|---|---|---|---|---|---|---|---|---|---|---|
| 不快 | -4 | -3 | -2 | -1 | 0 | 1 | 2 | 3 | 4 | 楽しい |

### 対話中に興奮していますか

| | 非常に | | やや | どちらともいえない | | | やや | | 非常に | |
|---|---|---|---|---|---|---|---|---|---|---|
| 落ち着く | -4 | -3 | -2 | -1 | 0 | 1 | 2 | 3 | 4 | わくわく |

### 対話に負担（ストレス）はありますか

| 全くない | | やや | | ある | | とてもある |
|---|---|---|---|---|---|---|
| 1 | 2 | 3 | 4 | 5 | 6 | 7 |

---

# Self-assessment Form(English version)

**Photo No.___________**

- **How do you feel about the dialogue about this photo? Please circle ○the number below.**

### Do you enjoy dialogue?

| | extremely | | slightly | neither | | | slightly | | extremely | |
|---|---|---|---|---|---|---|---|---|---|---|
| unpleasant | -4 | -3 | -2 | -1 | 0 | 1 | 2 | 3 | 4 | pleasant |

### Are you excited during the dialogue?

| | extremely | | slightly | neither | | | slightly | | extremely | |
|---|---|---|---|---|---|---|---|---|---|---|
| clam | -4 | -3 | -2 | -1 | 0 | 1 | 2 | 3 | 4 | excited |

### Do you feel burdened (stressed) by the dialogue?

| not at all | | slightly | | quite | | extremely |
|---|---|---|---|---|---|---|
| 1 | 2 | 3 | 4 | 5 | 6 | 7 |

## 脳波取得装置の快適性に関するアンケート(Japanese version)

1.電極キャップが重いと思いますか

□重くない　□やや重い　□重い　□とても重い

2.電極キャップが痛いですか

□痛くない　□やや痛い　□痛い　□とても痛い

3.電極キャップの着用はどのくらい許容できると思いますか

□10分未満　□10～30分　□31-60分　□1時間以上

4.柔らかい布製の電極がウェアラブルグラスやヘッドバンドに埋め込まれていたら、長時間着用していたいと思いますか?

□ 非常に　□やや　□ 不本意　□全く不本意

## Ultracortex Mark IV Wearable Comfort Evaluation(English version)

**1. Do you think the electrode cap is heavy?**

A. Not at all-1 B. A little-2 C. Heavy-3 D. Extremely-4

**2. At this point, do you feel any pain where your scalp touches the electrodes?**

A. Not at all-1 B. A little-2 C. Painful-3 D. Extremely-4

**3. How long do you think you can wear the Ultracortex Mark IV?**

A. No more than 10 min B. 10-30 min C. 31-60 min D. Over one hour

**4. If soft textile electrodes were embedded in wearable glasses or headbands, would you be willing to wear them for long periods of time during emotion recognition?**

A. Extremely B. A little C. Reluctant D. Totally reluctant

Options A, B, C, D are recorded as 1, 2, 3, 4 points respectively.
